# Analyzing longitudinal trait trajectories using GWAS identifies genetic variants for kidney function decline

**DOI:** 10.1038/s41467-024-54483-9

**Published:** 2024-11-20

**Authors:** Simon Wiegrebe, Mathias Gorski, Janina M. Herold, Klaus J. Stark, Barbara Thorand, Christian Gieger, Carsten A. Böger, Johannes Schödel, Florian Hartig, Han Chen, Thomas W. Winkler, Helmut Küchenhoff, Iris M. Heid

**Affiliations:** 1 Department of Genetic Epidemiology, University of Regensburg, Regensburg, Germany; 2 Statistical Consulting Unit StaBLab, Department of Statistics, LMU Munich, Munich, Germany; 3 Institute of Epidemiology, Helmholtz Zentrum München, German Research Center for Environmental Health (GmbH), Neuherberg, Germany; 4 German Center for Diabetes Research (DZD), Partner München-Neuherberg, Neuherberg, Germany; 5 Institute for Medical Information Processing, Biometry and Epidemiology (IBE), Faculty of Medicine, LMU Munich, Pettenkofer School of Public Health, Munich, Germany; 6 Research Unit of Molecular Epidemiology, Helmholtz Zentrum München, Neuherberg, Germany; 7 Department of Nephrology, University Hospital Regensburg, Regensburg, Germany; 8 Department of Nephrology, Diabetology, and Rheumatology, Traunstein Hospital, Southeast Bavarian Clinics, Traunstein, Germany; 9 KfH Kidney Centre Traunstein, Traunstein, Germany; 10 Department of Nephrology and Hypertension, Uniklinikum Erlangen and Friedrich-Alexander-Universität Erlangen-Nürnberg, Erlangen, Germany; 11 Theoretical Ecology, University of Regensburg, Regensburg, Germany; 12 Human Genetics Center, Department of Epidemiology, School of Public Health, The University of Texas Health Science Center at Houston, Houston, TX USA

**Keywords:** Genome-wide association studies, Kidney

## Abstract

Understanding the genetics of kidney function decline, or trait change in general, is hampered by scarce longitudinal data for GWAS (longGWAS) and uncertainty about how to analyze such data. We use longitudinal UK Biobank data for creatinine-based estimated glomerular filtration rate from 348,275 individuals to search for genetic variants associated with eGFR-decline. This search was performed both among 595 variants previously associated with eGFR in cross-sectional GWAS and genome-wide. We use seven statistical approaches to analyze the UK Biobank data and simulated data, finding that a linear mixed model is a powerful approach with unbiased effect estimates which is viable for longGWAS. The linear mixed model identifies 13 independent genetic variants associated with eGFR-decline, including 6 novel variants, and links them to age-dependent eGFR-genetics. We demonstrate that age-dependent and age-independent eGFR-genetics exhibit a differential pattern regarding clinical progression traits and kidney-specific gene expression regulation. Overall, our results provide insights into kidney aging and linear mixed model-based longGWAS generally.

## Abstract (web summary)

The authors use longitudinal data from the UK Biobank to search for genetic variants associated with kidney function decline. Using a linear mixed model, they identify 13 independent variants, incl. 6 novel, and link them to genetics of kidney aging.

## Introduction

Accelerated decline of kidney function is a serious health burden: it can lead to kidney failure, necessitating dialysis or kidney transplantation, with high risk of early mortality^1,2^ and otherwise limited therapeutic options. Kidney function is typically assessed by serum creatinine as estimated glomerular filtration rate (eGFR). Age-related decline of eGFR is on average −1 mL/min/1.73 m^2^/year in adult populations^3^, but exhibits a high variability due to mechanisms that are still poorly understood^4^.

**Fig. 1:**
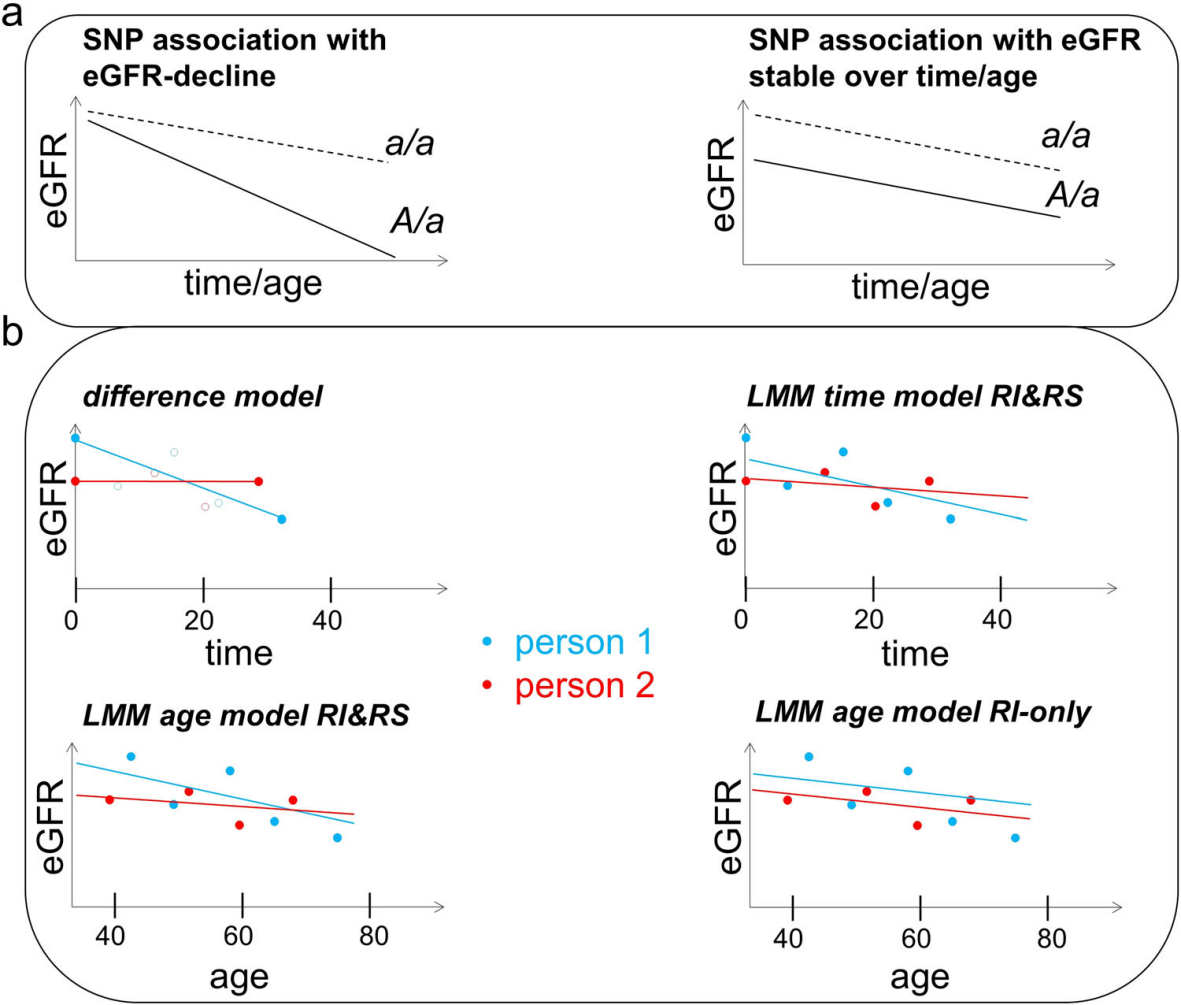
Conceptual illustration of genetic variant association with eGFR over time/age and phenotypic models. **a** Genetic variant (SNP) associations with eGFR can arise through one allele (risk allele *A*) that accelerates eGFR-decline over time/age (left) or lowers eGFR in a constant fashion over time/age (right) as compared to the other allele (*a*). This suggests that genetic variants associated with eGFR-decline are found among genetic variants associated with eGFR cross-sectionally. Shown is a schematic for persons with *A*/*a* versus *a*/*a*. **b** Temporal change of eGFR can be modeled in longitudinal data in various ways (phenotypic models): as (i) difference between last and 1st eGFR value of a person (*difference model*; assessments in-between 1st and last unused and thus depicted as circles); (ii) eGFR over time via linear mixed model (LMM) with person-specific intercepts and slopes (LMM *time model RI&RS*; time = 0 corresponds to an individual’s 1st eGFR assessment); (iii) eGFR over age (LMM *age model RI&RS*); or (iv) eGFR over age without random slopes (*LMM age model RI-only*; *time model RI-only* possible, but not applied/shown). Shown is a schematic of the phenotypic modeling for two example persons.

Deciphering the genetic make-up of kidney function decline by genome-wide association studies (GWAS) is a promising route to understand these mechanisms. Since genes in GWAS loci are candidates for drug development^5,6^, GWAS can also help identify therapeutic options. Hundreds of genetic loci have been identified for association with eGFR by large cross-sectional GWAS^7,8^. Cross-sectional associations may arise through one allele associated with steeper eGFR-decline or with lower eGFR-levels stable over time and age (Fig. 1a). Genes in decline-associated loci might lead more directly to therapeutic options to decelerate progression^9^. So far, only few genetic loci are known for genome-wide significant association with eGFR-decline: one locus (two variants in/near *UMOD*) in general populations (*n* = 343,339^10^; seven further loci among pre-selected variants at Bonferroni-corrected significance) and three loci in patients with chronic kidney disease (CKD, eGFR < 60 mL/min/1.73 m^2^, *n* = 116,870^11^).

This reflects a general imbalance between well-studied genetics of cross-sectional disease-related traits^12^ and less-studied genetics of temporal trait change using longitudinal data: there are only few robustly identified genetic variants for the temporal change of any trait^11,13,14^. This is despite the high clinical relevance, as deteriorating quantitative biomarkers are typically linked to disease onset and progression. The reason for this imbalance is arguably the scarcity of large longitudinal data, but also substantial uncertainty about the appropriate statistical approach that simultaneously achieves controlled type I error, high power, unbiased effect estimation, and computational speed.

Emerging large-scale longitudinal data from biobanks that integrate electronic health records (eHRs) set the stage for a new era of longitudinal GWAS (“longGWAS”). LongGWAS can address multiple questions, including the quest for genetics of trait variability^15^ or (here) the quest for genetics of temporal trait change.

There are various options to model temporal trait change (Fig. 1b): (i) a straightforward approach uses the difference divided by time in-between two eGFR assessments (*difference model*); linear mixed models (LMMs), a standard framework for longitudinal data^16^, can model the trait: as (ii) function of time-since-baseline (*time model*) or (iii) function of age (*age model*) with random intercepts and random slopes accounting for their correlation (*RI&RS*)^17^ or ignoring it (*RI&RS uncorrelated*; to improve identifiability^18^), or (iv) with random intercepts only (*RI-only;* computationally easier). LMMs can be applied to test genetic variants directly (one-stage LMM) or as computationally much faster two-stage approach (using LMM to generate “best linear unbiased predictors”, BLUPs, for person-specific slopes, evaluated via linear regression^11,19^; *BLUPs&LinReg*). Previous work applied the *difference model*^10,20^ or *BLUPs&LinReg*^11,21^, which are readily applicable for longGWAS by standard software, but cannot integrate individuals with = 1 trait assessment (“singletons”). One-stage LMMs can integrate singletons but are computationally challenging. So far, a systematic comparison between such approaches has been lacking.

Here, we set out to understand more about statistical approaches to test genetic association with temporal trait change, with eGFR-decline as role model, and about the genetics of eGFR-decline. We used simulated data and a UK Biobank (UKB) dataset on eGFR-trajectories combining creatinine values derived from study-center visits and eHRs^22^ (*n*~350 K; >1.5 million eGFR assessments over up to 27 years). Specifically, we (1) compared seven approaches regarding type I error, power, and bias and (2) searched the UKB eGFR-trajectories data for association with eGFR-decline. Since we hypothesized that eGFR-decline genetics was a subset of cross-sectional eGFR genetics, we searched for eGFR-decline association (2a) among 595 independent variants across 424 loci known for association with eGFR from cross-sectional GWAS^8,23^ (“595-search”), (2b) followed by longGWAS to evaluate this hypothesis.

## Results

### UKB eGFR-trajectories exhibit an approximately linear decline of −1 mL/min/1.73 m^2^/year

We analyzed unrelated European-ancestry UKB individuals without acute kidney injury (AKI) or nephrectomy, excluding eGFR assessments after onset of dialysis, kidney transplant, or end-stage kidney disease (ESKD) (“Methods” section). Our analyzed UKB data consisted of 149,263 individuals with ≥2 eGFR assessments per person (“UKB 150K”; median follow-up time = 8.4 years; *m* = 1,321,370 eGFR assessments) or 348,275 individuals with ≥1 eGFR assessment (“UKB 350K”; *m* = 1,520,382; Supplementary Fig. 1). UKB 350K was similar to 150K regarding participant characteristics: 54% women, 1.2% CKD at baseline and 4.6% at any timepoint (eGFR < 60 mL/min/1.73 m^2^), baseline age 35–78 years, median baseline eGFR = 97 mL/min/1.73 m^2^ (Table 1). We used UK10K/HRC-imputed allele dosages of 11.3 million single-nucleotide polymorphisms (SNPs) and selected 595 variants known for association with cross-sectional eGFR^23^ (“Methods” section).

**Table 1:** Participant characteristics for UKB data on eGFR-trajectories

	UKB 150K	UKB 350K
% (*n*) of women	53.7 (80,091)	53.7 (187,129)
Number of eGFR assessments per person	6 (2–289)	1 (1–289)
Follow-up time [years]	8.4 (1.0–27.1)	0.0 (0.0–27.1)
Age at 1st assessment [years]	55.9 (35.0–76.4)	57.1 (35.0–78.2)
Age at last assessment [years]	65.1 (37.0–79.7)	60.9 (36.0–79.7)
eGFR at 1st assessment [mL/min/1.73 m^2^]	98.0 (15.2–192.1)	97.4 (15.0–192.1)
eGFR at last assessment [mL/min/1.73 m^2^]	89.4 (15.0–198.6)	94.0 (15.0–198.6)
% (*n*) with CKD at 1st assessment	0.7 (1038)	1.2 (4069)
% (*n*) with CKD at any assessment	3.8 (13,116)	4.6 (16,147)

We show descriptive statistics for UKB individuals with ≥2 eGFR assessments (UKB 150K; *n* = 149,263, *m* = 1,321,370) and for the extended data adding individuals with =1 eGFR assessment (UKB 350K: *n* = 348,275; *m* = 1,520,382). The 199,012 individuals with =1 eGFR assessment have median age 58.2 (min–max 36.0–78.2) years and median eGFR 97.0 (min–max 15.0–159.6) mL/min/1.73 m^2^. CKD was defined as eGFR < 60 mL/min/1.73 m^2^. Shown is % (*n*) or median (min–max).

Before evaluating genetic variants, we explored a potentially non-linear relationship of eGFR with time and age, observing approximate linearity and negligible difference by sex (Supplementary Fig. 2a–c). This was more challenging for individuals with CKD, primarily due to regression-to-the-mean effects at the start of trajectories and sparse data at their end (Supplementary Fig. 2d). Assuming linearity, mean annual eGFR-decline was comparable across approaches (−0.88 to −1.08 mL/min/1.73 m^2^/year), with high variability of person-specific slopes (standard deviation 0.66–0.95 mL/min/1.73 m^2^/year, Supplementary Table 1 and Supplementary Note 1).

### LMM *age model RI&RS* is a powerful approach with unbiased genetic effect estimates

We considered seven approaches for genetic association analysis with eGFR-decline (Supplementary Table 2, “Methods” section Eqs. (1–4)): in data of individuals with ≥2 assessments over time, (i) *difference model*, (ii–v) four one-stage LMMs (*time model RI&RS*, *age model RI&RS*, *age model RI&RS uncorrelated*, *age model RI-only*), (vi) an LMM-based two-stage approach (*BLUPs&LinReg*); in data adding singletons (i.e., individuals with =1 assessment), (vii) *age model RI&RS*.

We compared these approaches in simulated data using various scenarios (simulation parameters corresponding to: eGFR-trajectories as in UKB 350K, ~50% singletons; eGFR-trajectories in an external cohort study, KORA-4^24^, ~20% singletons; trajectories of another trait, body mass index, BMI, in KORA-4; “Methods” section, Supplementary Table 3). We found the following (Table 2 and Supplementary Table 4): (i) type I error was inflated for *age model RI-only* and *age model RI&RS uncorrelated*, indicating insufficient accounting for person-specific slope variability. (ii) Power was better for one-stage LMMs compared to *difference model*, but *BLUPs&LinReg* was the most powerful. When adding singletons, not possible with *difference model* or *BLUPs&LinReg*, the *age model RI&RS* became nearly as powerful as *BLUPs&LinReg* in the UKB-based scenario. (iii) Biased effect estimates were observed for *BLUPs&LinReg* in all scenarios (11%–38% shrinkage), in line with the bias-variance trade-off known from regularization^25^ (Supplementary Note 2), while estimates from a*ge model RI&RS* were unbiased.

**Table 2:** Performance of seven approaches to genetic association analyses for trait change in simulated and empirical longitudinal data

	Simulated data			Empirical data			
	UKB scenario for eGFR-trajectories			UKB eGFR-trajectories			
Approaches	T1E [%] (CI)	Power [%] (CI)	Bias [%]	T1E [%] (CI)	“Power” in 9	“Bias” in 9	Identified in 595
**Without singletons**							
*Difference model*	5.1 (4.7, 5.5)	12.0 (11.4, 12.7)	0.6	4.9 (4.5; 5.3)	4/9	0.0	2 (2)
*Time model RI&RS*	4.7 (4.2, 5.1)	25.6 (24.7, 26.4)	0.0	4.8 (4.4; 5.2)	7/9	8.2	7 (2)
*Age model RI&RS*	4.8 (4.4, 5.3)	31.4 (30.5, 32.4)	0.7	5.0 (4.5; 5.4)	8/9	Reference	9 (2)
*Age model RI&RS uncorr*.	7.5 (7.0, 8.1)	38.8 (37.8, 39.8)	0.7	8.8 (8.2; 9.3)	8/9	0.7	13 (3)
*Age model RI-only*	32.4 (31.4, 33.3)	59.0 (58.1, 60.0)	0.6	43.1 (42.2; 44.1)	9/9	16.9	98 (41)
*BLUPs&LinReg*	5.3 (4.9, 5.8)	44.8 (43.9, 45.8)	−37.7	5.0 (4.6; 5.5)	8/9	−38.5	13 (3)
**Including singletons**							
*Age model RI&RS*	5.2 (4.8, 5.7)	44.1 (43.1, 45.1)	0.5	4.8 (4.3; 5.2)	8/9	0.2	12 (6)

We compared seven approaches (“Methods” section and Supplementary Table 2) regarding type I error, power, and bias: six approaches analyze individuals with ≥2 trait assessments over age/time (no singletons, i.e., individuals with =1 trait assessment), the 7th approach repeats *age model RI&RS* including singletons. Simulations were based on distributions of age, global/random trait effects, and random error as in UKB 350K for eGFR and simulated genotypes (EAF = 30%; 10,000 simulation runs; “Methods” section and Supplementary Table 3). This scenario covers a setting as in UKB (~50% singletons) for trajectories of a trait like eGFR with prounouced age effect on trait. We show estimates of type I error (T1E), power, and bias from 10,000 simulation runs. In empirical analyses using UKB 150K (no singletons) or 350K (including singletons), we show permutation-based type I error, proxies of power and bias (based on 9 SNPs known for eGFR-decline^10^), and number of SNPs identified with *P*_decline_ < 0.05/595 (*P*_decline_< 5 × 10^−8^) among the 595 SNPs known for association with cross-sectional eGFR^8^.

Simulated data: T1E = proportion of SNPs with *P*_decline_ < 0.05 across 10,000 simulated SNPs given zero true effect on decline, *β*_decline_ = 0 (95% CI using SEs from exact binomial test); Power = proportion of SNPs with *P*_decline_ < 0.05 across 10,000 simulated SNPs given true effect on decline, *β*_decline_ = −0.025 (95% CIs derived from SEs using exact binomial test); Bias = relative bias of effect estimates given true effect on decline, *β*_decline_ = −0.025, derived as average (across 10,000 simulation runs) of (${\hat{{{{\rm{\beta }}}}}}_{{{{\rm{decline}}}}}$−*β*_decline_)/*β*_decline_; Empirical data: T1E = proportion of SNPs with *P*_decline_ < 0.05 among 10,000 permutation-based “null-SNPs” using eGFR-trajectories of UKB individuals (95% CIs using SEs from exact binomial test); “Power” in 9 = proportion of SNPs directionally consistent with *P*_decline_ < 0.05 in UKB 150K (for *age model RI&RS*: additionally in UKB 350K) among the 9 SNPs known for eGFR-decline^10^; “Bias” in 9 = relative deviation of effect estimate from reference among the 9 SNPs known for eGFR-decline^10^ derived as average across the 9 SNPs of (${\hat{{{{\rm{\beta }}}}}}_{{{{\rm{decline}}}}}-{\hat{{{{\rm{\beta }}}}}}_{{{{\rm{decline}}}}({{{\rm{reference}}}})}$)/$\,{\hat{{{{\rm{\beta }}}}}}_{{{{\rm{decline}}}}({{{\rm{reference}}}})}$; Identified in 595 = number of SNPs with *P*_decline_ < 0.05/595 (in parentheses: with *P*_decline_ < 5 × 10^−8^) among 595 SNPs tested.

Empirical data (UKB 150K, or 350K when adding singletons) corroborated simulation findings regarding type I error (no control by *age model RI-only* and *RI&RS uncorrelated*, Supplementary Fig. 3), power (best for *BLUPs&LinReg* and *age model RI&RS* in UKB 350 K), and bias (*BLUPs&LinReg*: 38.5% shrinkage; Table 2, Supplementary Note 2, Supplementary Fig. 4, Supplementary Data 1).

Altogether, among approaches with type I error control, *BLUPs&LinReg* showed the best power, but biased effect estimates. When jointly aiming for good power and unbiased effect estimates, the LMM *age model RI&RS* was preferable, particularly in the UKB 350K dataset. We thus used the LMM *age model RI&RS* in UKB 350K in the following.

### Twelve genetic variants across ten loci identified for association with eGFR-decline

**Fig. 2:**
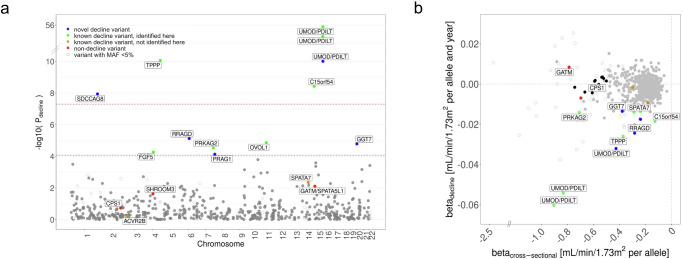
Twelve variants identified for eGFR-decline by focused search among 595 variants. We selected 595 SNPs previously reported for association with eGFR in cross-sectional data^23^ and tested them for association with eGFR-decline using the one-stage LMM *age model RI&RS 350K* (UKB 350K; *n* = 348,275, *m* = 1,520,382). **a** Shown are *P* values (*P*_decline_) versus chromosomal position. We identified 12 variants (10 loci) for eGFR-decline at Bonferroni(595)-corrected significance (*P*_decline_ < 0.05/595 = 8.4 × 10^−5^, brown dashed horizontal line; including 6 with *P*_decline_ < 5 × 10^−8^, red dashed horizontal line), consisting of 5 novel and 7 known variants for eGFR-decline^10^ (blue or green, respectively). Also color-coded are two variants known for eGFR-decline not identified here (orange) and three variants known for not being associated with eGFR-decline (red)^10^. Variants with small minor allele frequency (MAF < 5%) are shown as circles. **b** Shown are genetic effect sizes for eGFR-decline (*β*_decline_ from LMM *age model RI&RS* 350K) versus effect sizes for association with eGFR cross-sectionally (*β*_cross-sectional_: eGFR~sex, age, SNP, PCs; eGFR from UKB baseline study-center assessment, *n* = 341,073). Color and symbol codes are as in (**a**), additionally highlighting 11 stable-effect variants (black; *P*_main_ < 5 × 10^−8^, |*β*_main_| > 0.50 mL/min/1.73 m^2^/allele; *P*_decline_ ≥ 0.1; |*β*_decline_| < 0.005 and SE_decline_ < 0.005 mL/min/1.73 m^2^/allele and year) that include the *CPS1* variant (rs1047891; red in (**a**)). Effect allele was the cross-sectionally eGFR-lowering allele (unconditioned analyses in EUR^23^). The exact numerical values are provided in Supplementary Data 2.

Due to our hypothesis that genetics of eGFR-decline is a subset of genetics of cross-sectional eGFR, we first focused on the 595 variants known for cross-sectional eGFR-association^23^ and tested these for association with eGFR-decline (“595-search”, LMM *age model RI&RS* in UKB 350 K). We identified 12 variants (*P*_decline_ < 0.05/595 = 8.4 × 10^−5^, 6 with *P*_decline_ < 5 × 10^−8^, Fig. 2a and Table 3): (i) 7 variants known for eGFR-decline^10^ (near/in *UMOD*/*PDILT* (2), *TPPP*, *C15orf54, FGF5, OVOL1*, and *PRKAG2*) and (ii) 5 variants novel for eGFR-decline: 1 independent third *UMOD/PDILT* variant and 4 novel loci (near *SDCCAG8, RRAGD, GGT7, PRAG1*). We raised the number of variants with *P*_decline_ < 5 × 10^−8^ from two (*UMOD/PDILT*) to six (four loci, adding loci around *TPPP*, *C15orf54*, *SDCCAG8*; Table 3). Results were robust upon various sensitivity analyses (Supplementary Fig. 5 and “Methods” section).

**Table 3:** Twelve variants identified for association with eGFR-decline using LMM age model RI&RS in the UKB 350K dataset

			UKB 350K		UKB 150K		UKB 150K		CKDGen (Gorski et al.)	
			*age model RI&RS*		*age model RI&RS*		*difference model*		*difference model*	
SNPID	Locus	EA	*beta*_decline_	*P*_decline_	*beta*_decline_	*P*_decline_	*beta*_decline_	*P*_decline_	*beta*_decline_^a^	*P*_decline_
**Identified variants that were known for eGFR-decline (directly or per proxy with** ***r***^2^ > **0.8),** ***P***_decline_ < **0.05/595**										
rs77924615	*UMOD/PDILT*	G	−0.060	1.1E − 54	−0.059	2.2E − 32	−0.057	2.2E − 10	−0.074	5.3E − 38
rs13334589	*UMOD/PDILT*	A	−0.054	1.1E − 42	−0.055	1.0E − 26	−0.060	3.8E − 11	−0.066	2.1E − 31
rs434215	*TPPP*	A	−0.026	4.3E − 12	−0.024	1.1E − 06	−0.013	0.14	−0.020	3.7E − 04
rs28857283	*C15orf54*	G	−0.019	3.8E − 09	−0.021	3.8E − 07	−0.022	0.0026	−0.021	1.5E − 06
rs4930319	*OVOL1*	C	−0.014	1.3E − 05	−0.012	0.0032	−0.013	0.090	−0.015	9.9E − 04
rs10224002	*PRKAG2*	G	−0.014	2.9E − 05	−0.017	1.2E − 04	−0.019	0.015	−0.020	7.0E − 05
rs1458038	*FGF5*	C	−0.014	5.3E − 05	−0.015	7.5E − 04	−0.014	0.068	−0.019	3.9E − 05
**Identified variants that were novel for eGFR-decline,** ***P***_decline_ < **0.05/595**										
rs74209810	*UMOD/PDILT*	T	−0.032	6.6E − 11	−0.031	9.4E − 07	−0.035	0.0022	−0.017	0.028
rs2783971	*SDCCAG8*	A	−0.018	1.1E − 08	−0.019	2.9E − 06	−0.021	0.0035	−0.005	0.21
rs854922	*RRAGD*	A	−0.024	7.5E − 06	−0.026	2.7E − 04	−0.022	0.073	0.006	0.42
rs2076668	*GGT7*	A	−0.014	1.6E − 05	−0.014	4.0E − 04	−0.014	0.051	−0.005	0.24
rs2921093	*PRAG1*	T	−0.012	7.3E − 05	−0.012	0.0026	−0.014	0.056	0.003	0.46
**Variants identified for eGFR-decline by Gorski et al., but not identified here,** ***P***_decline_ ≥ **0.05/595**										
rs60503594	*SPATA7*	T	−0.009	0.004	−0.008	0.056	−0.003	0.69	−0.020	5.5E − 06
rs13064938	*ACVR2B*	C	−0.002	0.61	−0.001	0.78	−0.011	0.12	−0.013	3.0E − 03

In the 595-search^23^, we identified 12 variants for association with eGFR-decline at Bonferroni-corrected significance (*P*_decline_< 0.05/595 = 8.4 × 10^−5^; LMM *age model RI&RS*; UKB 350K dataset, *n* = 348,275, *m* = 1,520,382; Supplementary Data 1). Out of these 12 variants, 7 were known (identified previously for eGFR-decline^10^) and 5 were novel. Two variants identified previously were not identified here (*P*_decline_ ≥ 0.05/595). We compared the 12 + 2 variant results from *age model RI&RS* in UKB 350K with results from *age model RI&RS* in UKB 150K, *difference model* in UKB 150K, and *difference model* in CKDGen^10^ (*n*_CKDGen_ = 343,339, *m*_CKDGen_ = 686,678), which identified 5/2/0/0 novel variants (4/1/0/0 loci) for eGFR-decline, respectively. Of note, 2 out of 9 variants previously identified for eGFR-decline in CKDGen had been detected at *P*_decline_ < 5 × 10^−8^ (1 locus, *UMOD/PDILT*); the others were derived from searching a set of pre-selected variants judged at Bonferroni-corrected level (details in Supplementary Table 5). Shown are effect estimates (*beta*_decline_) and *P* values for eGFR-decline (*P*_decline_).

SNPID = variant identifier on GRCh37, Locus = nearest gene, EA = effect allele (cross-sectionally eGFR-lowering allele), *beta*_decline_ and *P*_decline_ = genetic effect and *P* value for eGFR-decline.

For the following SNPs identified for eGFR-decline by Gorski et al., a proxy variant is shown that was among the 595 SNPs: rs13334589 (proxy for rs34882080, *r*^2^ = 0.99), rs10224002 (proxy for rs10254101, *r*^2^ = 0.99), rs13064938 (proxy for rs13095391, *r*^2^ = 0.84), rs60503594 (proxy for rs1028455, *r*^2^ = 0.94), rs1145084 (proxy for rs2453533, *r*^2^ = 0.99), and rs28817415 (proxy for rs9998485, *r*^2^ = 0.49).

^a^*beta*s from the CKDGen summary statistics were multiplied by (−1) to align direction. The loci are labeled by the nearest gene of the region lead variant.

The five novel variants were detected with a similar number of individuals as in previous work^10^ (*n* ~ 350,000; CKDGen, *difference model*) due to the *age model*, not with the *difference model* in UKB or CKDGen or due to different multiple testing burdens (Table 3 and Supplementary Data 1).

Among the nine variants previously identified for eGFR-decline^10^, seven were identified here (*P*_decline_ < 0.05/595), one additional variant had *P*_decline_ = 5.1 × 10^−^^3^ (directionally consistent; Supplementary Table 5). We also confirmed variants near *CPS1*, *SHROOM3*, and *GATM* as not associated with eGFR-decline (*P*_declin__e_ ≥ 0.05, Supplementary Table 5).

### Validation in external data

We obtained support in independent longitudinal data: in three population-based cohort studies from Germany, we had previously reported an approximate linear relationship of eGFR over age^26^ (KORA-3: *n* = 2933, *m* = 3749; KORA-4: *n* = 3752, *m* = 9644; AugUR: *n* = 2397, *m* = 3442). Baseline age was 35–84, 25–74, or 70–95 years with ~20 years (KORAs) or ~9 years of follow-up (AugUR). The %CKD was higher in these studies than in UKB: %CKD at baseline (eGFR < 60 mL/min/1.73 m^2^) was 5.6%, 1.5%, and 21.5%, respectively, and %CKD at any timepoint was 6.7%, 8.2%, and 26.1%. The 12-variant polygenic score in combined KORA&AugUR data was significantly associated with eGFR-decline (*P*_decline_ = 0.013; *age model RI&RS*, “Methods” section).

### Decline-associated variants have little effect on eGFR for 40-year-old individuals and large effects on 70-year-old individuals in contrast to 11 stable-effect variants

When comparing directionality and size of variants’ effects on eGFR-decline with effects on cross-sectional eGFR (UKB study-center baseline, *n* = 341,073, aged 39–72 years), we found the 12 decline-accelerating alleles to coincide with cross-sectionally eGFR-lowering alleles (Fig. 2b, blue and green dots; Supplementary Data 2). One “bad” allele lowered average eGFR by −0.012 to −0.060 mL/min/1.73 m^2^/year compared to cross-sectional effects of −0.13 to −0.90 mL/min/1.73 m^2^ (Supplementary Data 3). We also observed variants with large cross-sectional effects that had no association with eGFR-decline (e.g., *CPS1* variant).

We extracted variants with large main effect on eGFR-levels and no association with eGFR-decline (*P*_main_ < 5 × 10^−8^, |*β*_main_| > 0.50 mL/min/1.73 m^2^ per allele, *P*_decline_ ≥ 0.1, |*β*_decline_| < 0.005 and SE_decline_ < 0.005 mL/min/1.73 m^2^ per allele and year), yielding 11 “stable-effect” variants (including *CPS1*; Supplementary Data 3). Their main effects, reflecting genetic effects on eGFR for 50-year-old individuals due to age-centering, were similar to cross-sectional effects (*β*_cross-sectional_ = −0.50 to −0.74 mL/min/1.73 m^2^; Fig. 2b, black dots).

**Fig. 3:**
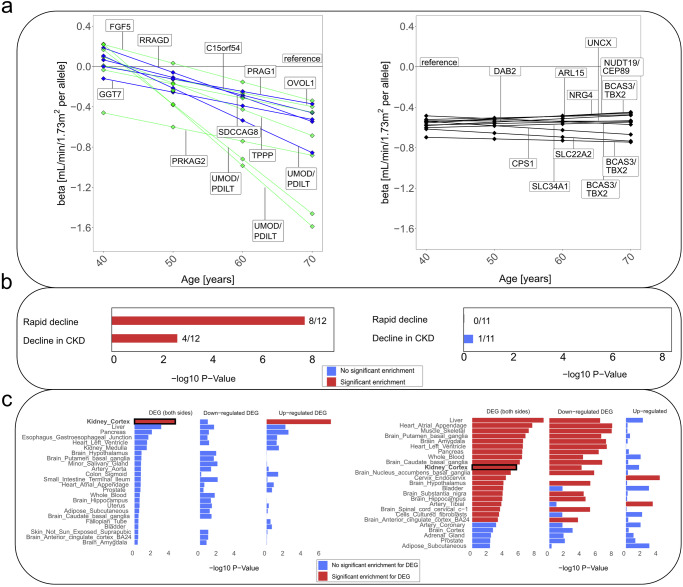
Differential pattern between decline-associated versus stable-effect loci regarding age-dependency, clinical progression traits, and tissue-specific gene expression regulation. We contrasted the 12 decline-associated variants versus 11 stable-effect variants and underlying loci. **a** Shown are genetic effects on eGFR for 40-, 50-, 60-, 70-year-old individuals using LMM *age model RI&RS* 350K (beta derived as *β*_main_ + (age-50)*β_decline_) for decline-associated variants (left; blue: novel, green: known) and stable-effect variants (right; black). Effect allele was the cross-sectionally eGFR-lowering allele^23^ (Supplementary Data 3). **b** We tested the 12 + 11 variants for association with two clinical progression traits using UKB 150K, rapid decline (*n*_cases_ = 1211, *n*_controls_ = 63,392, logistic regression) and decline in CKD (*n*_CKD_ =13,116, *m*_CKD_ = 116,944, LMM *time model RI&RS*; “Methods” section and Supplementary Table 6). Significant enrichment (*P*_enrich_ < 0.05) of directionally consistent nominally significant associations was found among the 12 (left; 8/12, 4/12), but not among the 11 SNPs (right; 0/11, 1/11). **c** We evaluated genes in loci of the 12 + 11 variants regarding tissue-specific enrichment of differentially expressed genes (DEGs): shown are enrichment *P* values in decline-associated loci (left, among 256 genes) and stable-associated loci (right, among 182 genes; using FUMA, testing 54 tissue types, showing top 25; “Methods” section). Significant enrichment for DEGs (FDR < 0.05, red) was found for decline-associated loci only in kidney cortex (upregulated) and for stable-effect loci in various tissues (mostly downregulated, e.g., in liver, heart, muscle, pancreas, kidney cortex).

We visualized the 12 + 11 SNP associations on eGFR-levels over age (*β*_main_ + (age-50)*β_decline_): the 12 decline-associated variants showed age-dependent effects on eGFR, while the 11 stable-effect variants showed age-independent effects (Fig. 3a). The large extent of age-dependency for decline-associated variants was remarkable: near-zero effects on eGFR-levels among 40-year-old (even *UMOD*/*PDILT*; except *PRAKG2*), but large effects for 70-year-old individuals, much larger than cross-sectionally (e.g., for *UMOD/PDILT* rs77924615: −1.59 versus −0.90 mL/min/1.73 m^2^ per “bad” allele, respectively; for rs854922 near *RRAGD*: −0.55 versus −0.28; Supplementary Data 3). This suggests that age-dependent associations with eGFR become effective mainly around the age of 40 years, while stable associations are already effective before the age of 40 years and age-independent thereafter.

### Robustness of findings regarding non-linear age effects and eGFR-variability

The approaches applied here and by others^10,11,20,21^ assume linearity in the global age effect on eGFR, the person-specific age effects on eGFR, and the age effect on the SNP-association with eGFR (i.e., modeling SNP-association with linear eGFR-decline). Allowing for non-linear relationships (adding quadratic terms; “Methods” section) did not alter results for the 12 + 11 SNP associations with linear eGFR-decline (Supplementary Fig. 6). Two variants, rs77924615 and rs13334589 in/around *UMOD/PDILT*, showed a small, but significant association with over-linear eGFR-decline (Supplementary Fig. 7; *P*_SNPxage²_ < 0.05/23 = 2.2 × 10^−^^3^; Supplementary Data 4). Further analyses for these two variants pointed to 50 years as breakpoint for accelerated decline (*P*_breakpoint50_ = 6.3 × 10^−^^56^ and 1.7 × 10^−5^, respectively; *P*_breakpoint40_ = 0.45 and 0.50, *P*_breakpoint60_ = 0.04 and 0.04; “Methods” section).

Longitudinal data have also been used to test for SNP associations with trait variability^15^. When applying the model implemented in *TrajGWAS*^15^ (“Methods” section), all 12 decline-associated variants, but also 7 stable-effect variants were associated with eGFR-variability (*P* < 0.05/23 = 2.2 × 10^−3^; Supplementary Fig. 8). Thus, association with eGFR-variability answers a different question than association with eGFR-decline.

### Decline-associated variants show SNP-by-age interaction in cross-sectional data

Decline-associated SNPs should show SNP-by-age interaction in cross-sectional data (UKB study-center baseline, *n* = 341,073; linear regression adjusted for sex, 20 principal components (PCs)): 10 of 12 showed *P*_SNPxage_ < 0.05; when compared to effects on eGFR-decline in longitudinal data, interaction effects were similar (−0.010 to −0.048 mL/min/1.73 m^2^ per allele and year) and *P* values were larger, attributable to reduced power (Supplementary Data 5). None of the 11 stable-effect variants had *P*_SNPxage_ < 0.05 with negative effect.

The cross-sectional data also gave us the opportunity to explore whether the age-dependency of the 12 SNP associations with eGFR was explained by their interaction with diabetes, HbA1c, hypertension, or systolic blood pressure (SBP). The SNP-by-age interaction effects remained the same when including SNP-by-diabetes, SNP-by-HbA1c, SNP-by-hypertension, or SNP-by-SBP interaction terms (Supplementary Fig. 9 and Supplementary Data 5).

### Differential pattern of association with clinical progression traits between decline-associated versus stable-effect loci

From a clinical perspective, rapid eGFR-decline or eGFR-decline in CKD are of particular interest as surrogate for CKD progression^3,27^. Previous work on the genetics of these progression traits identified SNPs around *UMOD/PDILT, PRKAG2*, and *TPPP*^11,20,21,28^, suggesting an overlap with genetics of eGFR-decline in general population. We tested the 12 + 11 SNPs for association with rapid decline (*n*_cases_ =1211, *n*_controls_ = 63,392; “Methods” section) and with eGFR-decline in the subset of individuals with CKD (eGFR < 60 mL/min/1.73 m^2^, *n*_ckd_ = 13,116, *m*_CKD_ = 116,944; “Methods” section). The 12 decline-associated variants were enriched for directionally consistent nominally significant association with rapid decline and eGFR-decline in CKD (*P*_enrich_ =1.6 × 10^−^^8^ or 2.2 × 10^−3^, respectively), but the 11 stable-effect variants were not (*P*_enrich_ = 1.0 or 0.43, respectively; Fig. 3b and Supplementary Table 6). Decline-associated variants contributing to these enrichments were near *UMOD*/*PDILT* (3)*, PRKAG2*, and *TPPP* (confirmed for clinical progression traits), *RRAGD*, *OVOL1*, and *C15orf54* (novel).

Both the 12 and 11 variants were enriched for association with the odds of having CKD (*n*_cases_ =16,147, *n*_controls_ = 332,128; *P*_enrich_ = 2.4 × 10^−16^ and 9.8 × 10^−11^, respectively). Thus, decline-associated versus stable-effect variants showed a similar relevance for having/developing CKD, but a differential pattern for clinical progression traits.

### Differential pattern of tissue-specific gene expression regulation in decline-associated versus stable-effect loci

We were interested in likely causal genes and potentially differential mechanisms implicated by the 12 decline-associated variants (10 loci) versus the 11 stable-effect variants (9 loci).

We annotated biological and statistical features to 256 and 182 genes in these loci (“Methods” section; Supplementary Data 6). We found accumulated evidence with ≥3 features for six genes to be likely causal for decline-associated loci (*UMOD*, *PRKAG2*, *SDCCAG8*, *RRAGD, TPPP*, *FGF5*) and for four genes for stable-effect loci (*CPS1, SLC22A2, SLC34A1*, *UNCX*; Table 4 and Supplementary Note 3). For the highlighted 6 + 4 = 10 genes, the locus index variant was in or very near (<25 kb) to the mapped gene and statistically highly likely the association-driving variant (22%–100% probability). Common-variant effects for Mendelian disease genes were found for both decline-associated and stable-effect variants; two genes known for a role in creatinine metabolism (creatinine production or tubular reuptake^29,30^) mapped to stable-effect loci.

**Table 4:** Genes supported as likely causal genes in decline-associated or stable-effect loci

Gene	Index variant: location (probability to be causal)	Index variant: functional consequence	Known phenotype	Locus type and novelty
**Genes mapped to decline-associated loci**				
*UMOD*	Near/in gene (100%, 13%, 6%)^a^	eQTL(+)	Mendelian^b^	Novel 3rd signal
*TPPP*	Nearest (90%)	eQTL(+)		Known decline
*FGF5*	Nearest (49%)	eQTL(−)		Known decline
*PRKAG2*	In gene (80%)		Mendelian^c^	Known decline
*SDCCAG8*	In gene (22%)		Mendelian^d^	Novel decline
*RRAGD*	In gene (93%)	5′ UTR	Mendelian^e^	Novel decline
**Genes mapped to stable-effect loci**				
*CPS1*	In gene (100%)	Missense	Creatinine	Stable effect
*SLC22A2*	In gene (43%)		Creatinine	Stable effect
*SLC34A1*	in gene (100%)		Mendelian^f^	Stable effect
*UNCX*	Nearest (61%)	eQTL(+)		Stable effect

We annotated 256 and 182 genes in 10 decline-associated and 9 stable-effect loci, respectively, for statistical and biological features: known human kidney disease (OMIM^59^ and other^39,60^); drug target^61^; gene nearest to index variant^62,67^; mapped by variant >10% statistically likely causal^23^ that altered protein, protein abundancy, or gene expression in kidney tissue^37,63,64^ (eQTL, ± indicating up/downregulation by eGFR-lowering allele; “Methods” section, Supplementary Data 6 and Supplementary Note 3). Shown are the 10 genes with ≥3 features that supported the gene as likely causal for the association, indicating key information on index variant (location, probability to be causal, functional consequence), gene (human kidney phenotype or role in creatinine metabolism), and locus (known or novel for decline association, stable-effect locus).

*eQTL* expression quantitative trait locus in kidney tissue, *5*′ *UTR* 5′ untranslated region.

^a^Three independent index variants in *UMOD*/*PDILT* locus.

^b^ADTKD (autosomal dominant tubulo-interstitial kidney disease).

^c^Glycogen storage disease of heart with kidney involvement (renomegaly).

^d^Bardet-Biedl syndrome 16 (retina-renal ciliopathy).

^e^Renal hypomagnesemia 7.

^f^Fanconi Renotubular Syndrome 2.

While pathway-enrichment analyses were inconclusive (using Panther^31,32^, “Methods” section and Supplementary Note 3), analysis of tissue-specific enrichment for differentially expressed genes (DEGs) showed a strikingly differential pattern (using FUMA^33^, “Methods” section): significant enrichment for DEGs (false discovery rate, FDR < 0.05) was found only in kidney cortex for decline-associated loci (upregulated), yet in various tissues for stable-effect loci (mostly downregulated; e.g., in heart, liver, muscle, pancreas, kidney cortex; Fig. 3c). This suggests that decline-associated versus stable-effect loci differentiate kidney-specific versus cross-organ regulation of gene expression.

### LMM-based longGWAS identifies five loci with genome-wide significance highlighting *MUC1* for eGFR-decline

We now applied the LMM *age model RI&RS* in UKB 350K using the GMMAT/MAGEE^34,35^ implementation, which implements this model in a more efficient way than lme4 (“Methods” section). We tested the 595 variants and corroborated that association statistics for both implementations, GMMAT/MAGEE versus lme4, were identical (Supplementary Fig. 10 and Supplementary Data 7).

We used GMMAT/MAGEE to conduct a longGWAS, testing ~11 million autosomal variants (UK10K/HRC-imputed^36^, “Methods” section). We obtained results within 5 days (256 cores, 1 TB RAM) with little evidence for population stratification (lambda = 1.06).

**Fig. 4:**
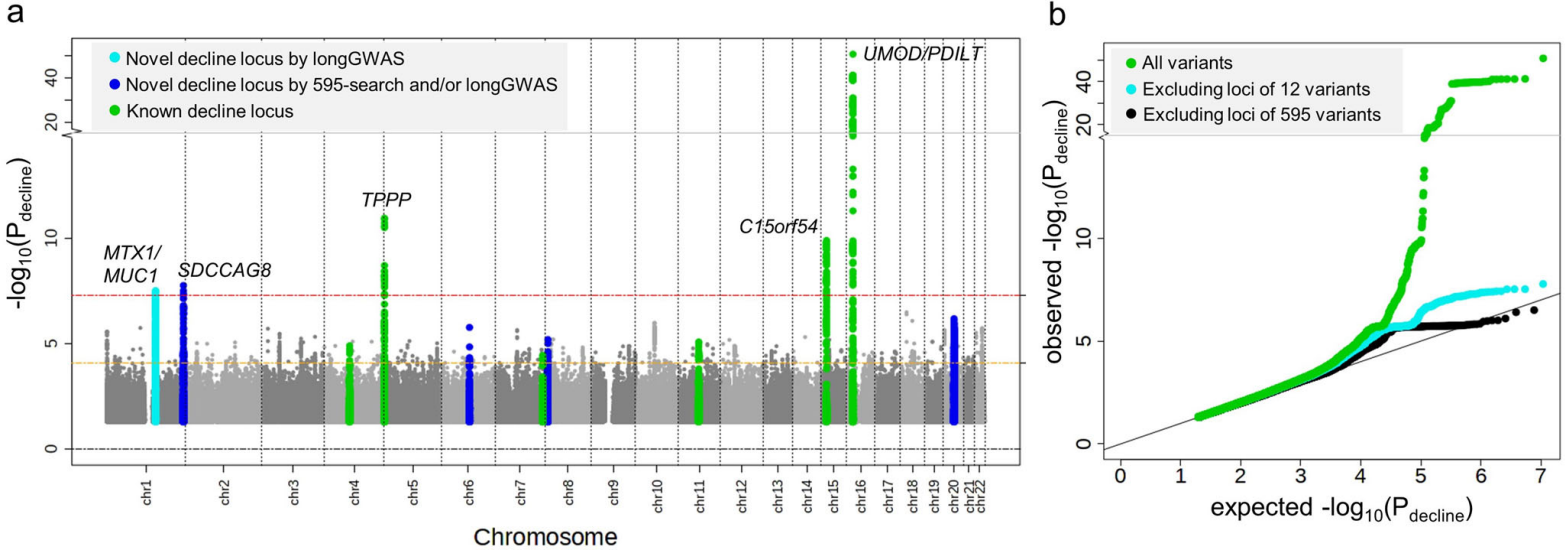
LongGWAS is viable with GMMAT/MAGEE and identifies five loci with genome-wide significance for eGFR-decline. We conducted a genome-wide search for genetic variant association with eGFR-decline (*P*_decline_, GC-corrected, lambda = 1.06) using the LMM *age model RI&RS 350K* implemented in GMMAT/MAGEE^34,35^ (UKB 350K; *n* = 348,275, *m* = 1,520,382; testing 11 million SNPs with MAF ≥ 0.5%, imputation quality INFO ≥ 0.6). **a** Shown are association *P* values versus chromosomal position. We identified five loci at genome-wide significance (*P*_decline_ < 5 × 10^−8^; red dashed horizontal line). Coloring highlights the overall 11 loci identified for eGFR-decline: 10 loci around the 12 variants identified by 595-search (*P*_decline_ < 0.05/595 = 8.4 × 10^−5^, brown dashed horizontal line; 4 novel and 6 known for eGFR-decline in blue or green, respectively), and one novel locus for eGFR-decline now identified by longGWAS (cyan; lead variant rs2075570 in the 424 loci, but not among the 595 variants). Loci were derived by clumping based on variant position (*d* > 500kB between loci, “Methods” section). **b** Shown is the Quantile–Quantile (QQ) plot comparing the distribution of observed *P*_decline_ with the distribution of *P*_decline_ expected under the null hypothesis of “no association with eGFR-decline” (green: all variants; cyan: excluding the 10 loci around the 12 decline-associated variants; black: excluding the 424 loci around the 595 variants).

We identified five loci associated with eGFR-decline at genome-wide significance (GC-corrected *P*_decline_ < 5 × 10^−8^, “Methods” section, Fig. 4): the four loci already identified with *P*_decline_ < 5 × 10^−8^ by the 595-search and one additional locus (*MTX1/MUC1*, novel for eGFR-decline compared to previous work^10^).

The lead variant of the *MTX1/MUC1* locus, rs2075570 (*P*_decline_ = 1.1 × 10^−8^), resided in the 424 loci known for cross-sectional eGFR, but was not among or correlated to the 595 variants (*P*_cross-sectional_ = 0.01 in Stanzick et al.^23^; *P*_cross-sectional_ = 0.80 in UKB; Supplementary Fig. 11a, b). Breakpoint analyses suggest a complex age-dependency of the rs2075570-association on eGFR (Supplementary Fig. 11c). rs2075570 modifies expression for *MUC1* in tubolo-interstitial tissue^37^, (FDR < 5%), which suggests *MUC1*, a well-known gene for rare autosomal dominant tubulo-interstitial kidney disease^38,39^, as likely causal gene.

In total, we identified 13 independent variants (11 loci) for eGFR-decline: 7 variants (5 loci) with *P*_decline_ < 5 × 10^−8^ by longGWAS and/or the 595-search and 6 variants (6 loci) by the 595-search (*P*_decline_ < 0.05/595; Supplementary Table 7). LongGWAS results also enabled us to show full regional association signals for decline-associated loci, which align well with respective signals from cross-sectional analyses (Supplementary Fig. 12), except for the *MTX/MUC1* signal (Supplementary Fig. 11a).

## Discussion

Based on UKB data on eGFR trajectories with >1.5 million datapoints and the one-stage LMM *age model RI&RS*, we identified known and novel SNP associations with eGFR-decline. Our results support the hypothesis that decline-associated variants reside in loci known for cross-sectional eGFR, but also that eGFR-decline associations can be masked in cross-sectional data by age effects. Methodologically, we showed that the one-stage LMM *age model RI&RS* was statistically advantageous for this task and, implemented in GMMAT/MAGEE, computationally viable for longGWAS. Importantly, it enabled the link of genetics of eGFR-decline to age-dependent genetics of eGFR with clinical and biological implications. Our work provides important insights into the genetics of kidney function decline and into pros and cons of statistical approaches for longGWAS.

With our results, we substantially raised the number of identified loci for eGFR-decline in general population, from 8^10^ to 11 (6 confirmed, 5 novel), and the number of genome-wide significant loci, from 1 (*UMOD/PDILT*) to 5. Biological annotation found evidence for three novel decline-associated loci to capture common-variant-effects for genes of rare Mendelian kidney diseases (*SDCCAG8*, *RRAGD*, and *MUC1*), additional to the two such genes in known eGFR-decline loci (*UMOD*, *PRKAG2*). The *TPPP* locus (known) was found to include a gene encoding an approved drug against CKD progression^40^ (*SLC9A3*), but *TPPP* was the statistically more likely causal gene^21^.

Our analyses also provide important insights into age-dependent versus age-independent genetics of eGFR: previously, one *UMOD* variant had been reported for age-dependent association with eGFR in cross-sectional data (*n* = 24,635^41^). We found all but one decline-associated variants with near-zero effects on eGFR for 40-year-old (even for *UMOD*) and large effects in 70-year-old individuals with up to twice the size of cross-sectional effects (e.g., near *RRAGD*). The mechanisms underlying decline-associated variants thus appear to become effective mainly from the age of 40 years onwards, in line with physiological kidney aging^42^. In contrast, mechanisms underlying the 11 stable-effect variants apparently become effective before the age of 40 years and remain age-independent thereafter. This underscored the advantage of the LMM *age model*, which enables the generation of age-appropriate genetic effects on eGFR that is not possible with *difference model*, *time model*, or *BLUPs&LinReg*.

Age-dependent versus age-independent genetics of eGFR differentiate biological processes and clinical implications: age-independent eGFR genetics identified here imply pathological or physiological processes affecting one’s predisposition to lower/higher eGFR at early adulthood that are stable over time. Stable-effect variants were associated with increased risk of CKD, but not with CKD progression. The underlying genes showed differential expression in numerous tissues including heart, liver, muscle, pancreas, and kidney, suggesting mechanisms that affect multiple organs. Stable-effect variants mapped to Mendelian kidney disease genes (*SLC34A1*), but also to creatinine metabolism (*CPS1, SLC22A2*^29,30^*)* in line with differential expression in muscle.

Age-dependent eGFR genetics imply processes that are dynamic over age, which can be mechanisms of kidney aging^43–45^ or age-accumulating pathological events. In a dataset where individuals are rather healthy and individuals with AKI excluded, like here in UKB^46^, such pathological events could stem from age-accumulating external stressors that are common on population-scale (such as diabetes and hypertension^34^, (poly-)medication intake, infections, or age-related decreased immune defense). However, in this UKB data, the age-dependency of genetic effects on eGFR was independent of interaction with diabetes or hypertension, which does not support a primary role of diabetes or hypertension. The observed kidney-specificity of gene expression regulation in decline-associated loci suggests kidney-inherent mechanisms. Causal genes in decline-associated loci might be compelling targets for the study of kidney aging mechanisms, like physiological aging by nephron loss^44^, or subsequent remodeling of remaining nephrons to compensate function^4,45^. Our results suggest an overlap of eGFR-decline genetics in general population with genetics of CKD progression, as many decline-associated variants were associated with rapid decline or decline in CKD. However, challenges in these analyses include potential index event bias^47^ when restricting to CKD, bias in *BLUPs* used to define rapid decline, and limited sample size for both. Future larger datasets may help understand the overlapping or discriminating processes of physiological kidney aging versus processes that lead to progressive disease, which is considered a promising route to identify therapeutic targets^45^.

Methodologically, we provide important insights into the conduct of longGWAS for eGFR-decline in adult population that are generalizable to other datasets and traits in various ways. Our simulations revealed that *BLUPs&LinReg* had excellent power and calibrated type I error, but exhibited bias in effect estimates due to regularization^25,48^. This may be acceptable for locus identification, but it is disadvantageous when the study aim is to interpret effect sizes or to use them in meta-analyses. When looking for an unbiased estimator with calibrated type I error, the LMM *age model RI&RS* is preferable. The computational burden of this model is relatively high, but its implementation in GMMAT/MAGEE makes it viable for longGWAS in large data, filling an important gap and complementing other longGWAS software targeting trait variability (e.g., *TrajGWAS*^15^).

A further methodological aspect of our study that is generalizable is modeling the longitudinal trait over age: it avoids the *time model*’s differentiation between temporal effects before and after baseline, which is unnecessary when baseline is a random timepoint that does not mark an intervention. We recommend the *age model* for longGWAS on trait change when the trajectory start is random and the *time model* when the trajectory start is informative, e.g., when analyzing trait change in patients.

We acknowledge that we analyzed only individuals of European ancestry and thus missed the *APOL1* locus, identified by others including African Ancestry^11^. Also, we relied on serum creatinine as biomarker to assess kidney function, which depends on muscle mass, and muscle mass declines by age^49^; this might have masked some of the age-related eGFR-decline. Genes with a role in creatinine metabolism were captured by stable-effect loci (CPS1^29^, *SLC22A2*^30^). We did not account for informative loss-to-follow-up or competing death; previous work using bivariate analyses found no impact of death as a second outcome^17^. Our primary LMM assumed a linear change in eGFR over age or time and derived SNP associations with linear eGFR-decline, which we found reasonable in our data, but requires evaluation in each setting.

Overall, our results provide important insights into age-dependent genetics of kidney function, which can help understand processes in kidney aging. Our methodological considerations, with kidney function decline as role model, inform future longGWAS regarding pros and cons of statistical approaches. Computationally efficient longGWAS along with the emerging large-scale longitudinal data from biobanks offer a promising route to understand the dynamics of genetic associations for disease markers and underlying mechanism.

## Methods

### Ethics

This UKB project was conducted under the application number 20272. The AugUR study was approved by the Ethics Committee of the University of Regensburg, Germany (vote 12-101-0258). The KORA-S3 study was approved by the local authorities and conducted in accordance with the data protection regulations as part of the World Health Organization Monitoring Trends and Determinants in Cardiovascular Disease (MONICA) Project. All other KORA studies were approved by the Ethics Committee of the Bavarian Chamber of Physicians (KORA-F3 EC Number 03097, KORA-S4 EC Number 99186, KORA-F4/FF4 EC Number 06068, KORA-Fit EC Number 17040). All studies comply with the 1964 Declaration of Helsinki and its later amendments, and all participants provided written informed consent.

### UKB eGFR-trajectories data

In UKB, an observational study of ~500,000 participants, we used serum creatinine measurements from blood drawn at study-center visits (centralized measurements, Enzymatic Beckman Coulter AU5800). We obtained further serum creatinine values and information on AKI, nephrectomy, dialysis, transplantation, and ESKD from general practitioner eHRs^22^ (GP CTV3 and read V2 codes). We combined eHR and study-center data and computed eGFR (ancestry-term-free CKD-EPI 2021^50^).

We included unrelated UKB participants of European ancestry^51^ without any eHR-record of AKI or nephrectomy and without eHR-record of dialysis, kidney transplant, or ESKD prior to their first eGFR assessment. We excluded eGFR assessments (i) before age of 35 years or January 1st, 1990, (ii) at or after eHR-record of dialysis, (iii) <6 months prior to, at or after eHR-record of kidney transplant or ESKD, (iv) after prior eGFR<15 mL/min/1.73 m^2^, and (v) extreme values (excluding absolute value > 10 residual SDs using LMM *age model RI&RS* in UKB 350K; winsorizing remaining eGFR values <15 and >200 mL/min/1.73 m^2^). We analyzed individuals with ≥2 eGFR assessments ≥1 year apart (UKB 150K), and, where applicable, added individuals with =1 eGFR assessment (UKB 350K).

Data processing and statistical analyses were performed using R-Software v4.0.4^52^. All statistical tests applied were two-sided.

### Genetic UKB data and pre-selection of genetic variants known for cross-sectional association with eGFR

We used UKB genomic data imputed to HRC^53,54^ and UK10K haplotype reference panels^55^ and 20 genetic PCs from Pan-UKB project^51^. We excluded variants with low imputation quality (Info < 0.6) or MAF < 0.5%, yielding allele dosages of 11,321,495 genetic variants. We selected 595 SNPs with genome-wide significant association with cross-sectional eGFR (CKDGen&UKB, *n* = 1,201,929^23^): (i) 594 independent index variants across 424 loci, (ii) one additional variant (rs28857283 near *C15orf54;* *P*_cross-sectional_ = 1.9 × 10^−8^) capturing a narrowly missed second signal in one of the 424 loci. The 595 SNPs included the 9 SNPs (directly or proxy by *r*^2^ ≥ 0.8) previously identified for association with eGFR-decline (*n* = 343,339^10^). Effect allele was the cross-sectionally eGFR-lowering allele (unconditioned analyses in EUR^23^).

### Seven approaches to identify SNP associations with temporal trait change

The following is stated for eGFR, but generalizes to any quantitative trait. For all approaches, $i$ denotes individuals ($i=1,\ldots,n$), ${n}_{i}$ the corresponding number of eGFR assessments ($t=1,\ldots,{n}_{i}$), ${ag}{e}_{i,t}$ and ${{eGFR}}_{i,t}$ the age and eGFR at the *t*th timepoint, and ${{SNP}}_{i}$ the allele dosage for a genetic variant (omitting indexing for the different SNPs). All SNP-association models were adjusted for 20 PCs (${{PC}}_{1,i},\,\ldots,\,{{PC}}_{20,i}$) (omitted in the following equations). Error terms ${\epsilon }_{i}$ or ${\varepsilon }_{i,t}\sim N\left(0,{\sigma }^{2}\right)$ are i.i.d. (and independent of RI&RS). We tested the SNPs for association with eGFR-decline by the following *six approaches* in data of individuals with ≥2 eGFR assessments:

(i) *difference model*^10,20^,
  1 $$\frac{{{eGFR}}_{i,{n}_{i}}-{{eGFR}}_{i,1}}{{ag}{e}_{i,{n}_{i}}-{ag}{e}_{i,1}}={\beta }_{0}+{\beta }_{1}*{SN}{P}_{i}+{\epsilon }_{i}$$
(ii) LMM *time model RI&RS* (with RI ${\gamma }_{0i}$ and RS ${\gamma }_{1i}$ from bivariate normal distribution, allowing for correlation) that models eGFR-levels as function of time-since-baseline (${tim}{e}_{i,t}$) and SNP-association with eGFR-decline as ${{time}}_{i,t}*{SN}{P}_{i}$ interaction, adjusting for age-at-baseline (${ag}{e}_{i,1}$),
  2 $${eGF}{R}_{i,t}= 	 \, {\beta }_{0}+{\beta }_{1} * {se}{x}_{i}+{\beta }_{2} * {ag}{e}_{i,1}+{\beta }_{3} * {{time}}_{i,t}+{\beta }_{4} * {SN}{P}_{i} \\ 	+{\beta }_{5} * {{time}}_{i,t} * {SN}{P}_{i}+{\gamma }_{0i}+{\gamma }_{1i} * {tim}{e}_{i,t}+{\varepsilon }_{i,t}$$
(iii) LMM *age model RI&RS*, equivalent to (2) but now modeling eGFR as function of age-at-exam (*age*_*i,t*_) and SNP-association with eGFR-decline as *age*_*i,t*_ ∗ *SNP*_*i*_ interaction:
  3 $${eGF}{R}_{i,t}= 	 {\beta }_{0}+{\beta }_{1} * {se}{x}_{i}+{\beta }_{2} * {ag}{e}_{i,t}+{\beta }_{3} * {SN}{P}_{i}+{\beta }_{4} * {{age}}_{i,t} * {SN}{P}_{i} \\ 	+{\gamma }_{0i}+{\gamma }_{1i} * {ag}{e}_{i,t}+{\varepsilon }_{i,t}$$
(iv) LMM *age model RI&RS uncorrelated*, where ${\gamma }_{0i}$ and ${\gamma }_{1i}$ are from independent univariate normal distributions,
(v) LMM *age model RI-only*, without RS term:
  4 $${eGF}{R}_{i,t}= 	 {\beta }_{0}+{\beta }_{1} * {se}{x}_{i}+{\beta }_{2} * {ag}{e}_{i,t}+{\beta }_{3} * {SN}{P}_{i}+{\beta }_{4} * {{age}}_{i,t} \\ 	 * {SN}{P}_{i}+{\gamma }_{0i}+{\varepsilon }_{i,t}$$
(vi) *BLUPs&LinReg*^11,21^, a two-stage approach (a) estimating RS terms, ${\hat{\gamma }}_{1i}$, via BLUPs based on LMM *age model RI&RS* (as in (3) without SNP as covariate) and (b) using ${\hat{\gamma }}_{1i}$ as outcome for SNP-association via linear regression (as in (1)).

In a *seventh* approach, we repeated the *age model RI&RS* in extended data adding individuals with =1 eGFR assessment (*age model RI&RS* including singletons).

All approaches make use of the entire trajectories (*n*_*i*_ ≥ 2; *n*_*i*_ ≥ 1 for the 7th approach), except the *difference model* which utilizes only two values over time (e.g., 1st and last). For analyses, we divided age and time by 10 and centered age at 50 years, ensuring appropriate scaling for optimization of LMMs (re-scaling results for all presentations). LMMs were fitted using *lmer()* (R-package *lme4*^56^ v1.1.34; Powell’s BOBYQA optimizer^57^).

### Evaluating type I error, power, bias in effect sizes, and detectability of eGFR-decline variants for the seven approaches

We simulated datasets for three phenotypic scenarios: (i) we used observed age-at-exam for randomly sampled UKB 350K individuals and simulation parameters (derived from UKB 350K, ~50% singletons); (ii + iii) we simulated a cohort study scenario (~20% attrition between baseline and follow-up, 20% singletons) with simulation parameters from the independent KORA-4 study^26^ for eGFR or BMI, respectively (details on simulation parameters in Supplementary Table 3). For each scenario, genotypes, random effects, and residual errors were simulated (10,000 times), then phenotypes were generated according to Eq. (3) without sex effects, with true SNP-association *β*_change_. For each approach, we computed type I error rates (proportion of nominally significant SNPs, *P*_change_ < 0.05, *β*_change_ = 0), power (proportion of nominally significant SNPs, *P*_change_ < 0.05, *β*_change_ ≠ 0), and bias (estimated genetic effect relative to *β*_change_ ≠ 0).

To evaluate empirical type I error, we generated 10,000 “null-SNPs” for UKB individuals (permutation of allele dosage of 500 out of the 595 SNPs, 20 times) and derived, for each approach, the proportion of SNPs with *P*_change_ < 0.05 as type I error estimate. We computed empirical power and bias based on the nine SNPs known for eGFR-decline^10^ as proportion of SNPs directionally consistent (*P*_change_ < 0.05; power) and mean relative difference of observed genetic effects compared to reference (bias). Finally, we derived detectability by testing 595 SNPs for association with eGFR-decline (judged at *P*_change_ < 0.05/595 = 8.4 × 10^−5^).

### Validation in external data

We used independent population-based longitudinal data from three studies, KORA-3, KORA-4, and AugUR from Germany^26^. Recruitment was via population registry, inviting randomly selected inhabitants of Augsburg (KORAs) or Regensburg (AugUR) of specific age range to participate. We tested the joint effect of identified decline-associated variants as PGS (sum of eGFR-decline-accelerating alleles weighted by *β*_decline_) for association with eGFR-decline (*age model RI&RS* including singletons; adjusting for study membership).

### Allowing for non-linear age effects

The LMM framework enables alleviating the linearity assumptions by, e.g., fitting 2nd degree polynomials for the relationships of age with (i) global eGFR (adding age^2^), (ii) person-specific eGFR-trajectories (adding age^2^ to the random effect), or (iii) SNP associations with eGFR (adding SNP*age^2^). We added these quadratic terms to the original model (LMM *age model RI&RS* in UKB 350K; eGFR~SNP, age, SNPxage, sex, PCs, RI, RS) and explored their impact on the SNP-by-age effect (i.e., SNP-association with linear eGFR-decline). For SNPs with *P*_SNPxage²_ < 0.05, we additionally conducted breakpoint analyses (allowing for interval-wise linear relationships at 40, 50, and 60 years of age).

For eGFR-variability analyses, we used a generalized additive model for location, scale and shape (GAMLSS)^58^ with *µ*(eGFR)~sex, age, SNP, PCs and log(*σ*(eGFR))~sex, age, SNP, PCs.

### Follow-up of identified variants regarding association with clinical traits

Rapid decline cases and controls were defined as annual decline < −3 or −1 to +1 mL/min/1.73 m^2^, respectively (based on estimated person-specific slopes via BLUPs, Eq. (3) without SNP as covariate); SNPs were tested for association with rapid decline via logistic regression (adjusted for age-at-baseline, sex, PCs). For eGFR-decline in CKD, we selected individuals with CKD (eGFR < 60 mL/min/1.73 m^2^) for at least one timepoint, removing the eGFR-trajectory before the first such timepoint; SNPs were tested for association with eGFR-decline in these CKD individuals (LMM *time model RI&RS*, since now the first timepoint is informative; Eq. (2)). UKB 150K was used, since these analyses required ≥2 eGFR values over time.

We also tested SNPs for association with being in the CKD subset (cases = CKD at any timepoint, controls = no CKD at any timepoint; using UKB 350K) via logistic regression (adjusted for age-at-CKD-onset or age-at-baseline, sex, PCs).

### Follow-up of identified variants regarding biological relevance

Using KidneyGPS^23^, we annotated genes in identified loci for features that supported them as likely causal: (i) Mendelian human kidney disease (OMIM^59^ and other^39,60^), (ii) drug target for registered clinical trials on kidney disease (Therapeutic Target Database^61^), (iii) nearest gene to index variant^62^, (iv) gene mapped to variant statistically likely to be causal (posterior probability of association ≥10%) which alters protein (e.g., “missense”), protein abundance (e.g., 5′ UTR), or gene expression in kidney tissue (eQTL, Neptune^63^, Susztak Lab^37^, GTExv8^64^; FDR < 5%). Notably, we used fine-mapping cross-sectionally assuming association signals for eGFR-decline to coincide with cross-sectional association signals as indicated previously^10^.

We searched genes in identified loci for enrichment of pathways (Reactome version-85, Released 2023-05-25, using PANTHER 18.0^31,32^) or tissue-specific enrichment of DEGs (MAGMA^65^ as GENE2FUNC in FUMA 1.5.2^33^ with default parameters, which evaluates 54 different tissue types).

### LongGWAS on eGFR-decline in UKB

We tested 11,321,495 autosomal variants from UK10K/HRC-imputed UKB data^36^ using LMM *age model RI&RS* in UKB 350K via GMMAT (v1.4.2)^34^ and MAGEE (v1.4.1)^35^. GMMAT/MAGEE provides an efficient implementation of an LMM RI&RS. The computational efficiency is obtained by estimating the LMM-based phenotypic variance-covariance only once (GMMAT), which is then used by MAGEE to efficiently test SNP associations. Analyses were adjusted for 20 PCs; results were corrected for GC lambda^66^. We selected genetic variants associated with eGFR-decline with GC-corrected *P*_decline_ < 5 × 10^−8^. Independent locus regions were defined by the variant with the smallest *P*_decline_ (lead variant) and variants nearby ±250 kb (overlapping loci merged).

### Reporting summary

Further information on research design is available in the Nature Portfolio Reporting Summary linked to this article.

## Supplementary information

Supplementary Information

Description of Additional Supplementary Files

Supplementary Data 1

Supplementary Data 2

Supplementary Data 3

Supplementary Data 4

Supplementary Data 5

Supplementary Data 6

Supplementary Data 7

Reporting Summary

## Source data

Source Data

## Data Availability

This UK Biobank project was conducted under the application number 20272. UK Biobank is a publicly accessible database. Individual participant data from UKB are available via the UK Biobank resource. Individual participant data from KORA-3, KORA-4, and AugUR are not publicly available due to data protection regulations and restrictions imposed by the Ethics Committee of the Bavarian Chamber of Physicians to protect participant privacy. However, data can be accessed upon request through project agreements with KORA (https://helmholtz-muenchen.managed-otrs.com/external) or AugUR (augur@ukr.de). For the reproducibility of our results, we provide the source code for the various statistical approaches applied here (see “Code availability” section). We also provide the source code for the simulation studies and for the real data analysis with GMMAT/MAGEE. We provide genetic variant association summary statistics (see Supplementary Data). Source data are provided with this paper.

## Code availability

The code to run the seven approaches, the GMMAT/MAGEE analysis, and the simulations is available on GitHub (www.github.com/genepi-regensburg/UKB_KidneyFunctionDecline; 10.5281/zenodo.13879592).
