# Supplementary material for: Analyzing longitudinal trait trajectories using GWAS identifies genetic variants for kidney function decline
Source: Nat Commun. 2024 Nov 20;15:10061. doi: 10.1038/s41467-024-54483-9 (PMC11579025; doi:10.1038/s41467-024-54483-9)
Supplement: Supplementary file 1 — Supplementary Information [file 41467_2024_54483_MOESM1_ESM.pdf]

Supplementary Information

Analyzing longitudinal trait trajectories using GWAS identifies genetic variants for kidney function decline

Supplementary Notes... 3

Supplementary Note 1: Some aspects about modeling the eGFR relationship over age or time... 3

Supplementary Note 2: Comparison of the seven approaches – theory and observations in simulated and empirical data 4

Supplementary Note 3: Annotation of genes in decline-associated and stable-effect loci... 6

Supplementary Figures... 8

Supplementary Figure 1: Inclusion and exclusion of UKB individuals for analyses............ 8

Supplementary Figure 2: Relationship of eGFR with age and time allowing for non-linearity. 9

Supplementary Figure 3: Visualization of permutation-based type I error via QQ plot across all seven approaches. 10

Supplementary Figure 4: Comparison of genetic effect estimates and standard errors for eGFR-decline between approaches. 11

Supplementary Figure 5: Sensitivity analyses for the LMM age model RI&RS 350K.........14

Supplementary Figure 6: SNP-association for eGFR-decline under non-linear modeling of age effects. 17

Supplementary Figure 7: The age-dependency of SNP effects on eGFR is approximately linear even under non-linear modeling. 19

Supplementary Figure 8: SNP effects on eGFR-decline versus SNP effects on eGFR-variability for the 12 decline-associated and 11 stable-effect variants. 20

Supplementary Figure 9: Independence of SNP-by-age interaction on eGFR in cross-sectional data upon adjusting for SNP-interaction with diabetes or hypertension. 21

Supplementary Figure 10: Comparison of GMMAT/MAGEE and lme4 implementation of LMM *age model RI&RS 350K* for the 595 SNP-associations with eGFR-decline. 22

Supplementary Figure 11: Summary of results for the *MTX1/MUC1* locus. 23

Supplementary Figure 12: Regional association for loci of 12 decline-associated variants. 24

Supplementary Tables 27

Supplementary Table 1: Annual eGFR-decline estimates across approaches without genetics. 27

Supplementary Table 2: Model specifications of seven approaches for genetic association analysis with trait trajectories. 28

Supplementary Table 3: Parameter specifications for simulation scenarios. 29

Supplementary Table 4: Performance of seven approaches to genetic association analyses for trait change in independent simulated longitudinal data. 30

Supplementary Table 5: Replication of eight out of nine known variants for eGFR-decline. 31

Supplementary Table 6: Association of decline-associated and stable-effect variants with clinical traits 32
Supplementary Table 7: 13 variants across 11 loci identified for association with eGFR-decline via longGWAS and/or 595-search....................33
Supplementary References 34

Supplementary Notes

Supplementary Note 1: Some aspects about modeling the eGFR relationship over age or time

When the timepoint of the 1st assessment ("baseline") does not mark the start of an intervention, but rather a random timepoint, the *time model* is an over-parametrization for the trait change – separating the trait change before and after baseline (covariates age-at-baseline, time-since-baseline). The *age model* uses one parameter (covariate age-at-exam) based on the notion that the time-since-baseline implies nothing but the aging since baseline. This "aging" includes everything that is accumulated or more likely with older age: e.g., higher probability of (multi-)medication intake, weight gain, hypertension, type 2 diabetes, cardiovascular disease, or reduced infection defense. The time-since-baseline effect is then equivalent to the ageing effect after baseline. Our results from the *time model* (with RI&RS) showed that the annual eGFR-decline estimates before and after baseline were slightly different (-0.71 [-0.72, -0.70] and -1.08 [-1.08, -1.07] mL/min/1.73m^2 per year, respectively); **Supplementary Table 1**). The annual eGFR-decline estimate using the *age model* (with RI&RS, same data) was -0.97 [-0.97, -0.96] mL/min/1.73m^2 per year, supporting the notion that the *age model* estimate for annual decline is an average of the decline before and after baseline. The confidence intervals for the age and time coefficient estimates were very narrow in all models, underscoring the high power in this large data.

The high variability of person-specific slopes (given by the standard deviation of the random slopes) is noteworthy: standard deviations ≥0.7 mL/min/1.73m^2 indicate that, while individuals have an average annual decline of about -1.0 mL/min/1.73m^2 per year, 68% of individuals vary between having -0.3 and -1.7 mL/min/1.73m^2 per year.

Of note, our main aim was not to derive the best model for the age or time association with eGFR, but to provide a reasonable framework for testing SNP-associations with eGFR-decline. Given the genotype is determined by conception, the genetic variant association is best estimated as average effect on eGFR before and after baseline (i.e., using the *age model*).

Supplementary Note 2: Comparison of the seven approaches – theory and observations in simulated and empirical data

Here, we describe our observations of type I error, power and bias in simulated data and empirically (UKB 150K unless stated otherwise, testing 595 variants known for cross-sectional eGFR association[1]) in more detail and complement them with expectations from theory.

(i) We found substantial type I error inflation for *age model RI-only* in simulations (**Table 2**, **Supplementary Table 4**) and empirically (**Supplementary Figure 3**), as well as strongly deflated SEs in empirical data as compared to *age model RI&RS* (**Supplementary Figure 4C**). This was expected from theory: RI-only models are known to produce deflated standard errors (SEs) and increased type I errors when there is between-individual variability in the age (or time) effect[2]. Therefore, the large number of variants identified by *age model RI-only* are mostly false positives. We also found some degree of type I error inflation for the *age model RI&RS uncorrelated* in UKB-based simulations and empirically (**Table 2**, **Supplementary Figure 3**) and deflated SEs in empirical data (**Supplementary Figure 4D**). This suggests that the random effects structure with this model has not been flexibly enough. Using a permutation-based approach in the empirical UKB data, we also found type I error inflation very similar to the one found in simulations for both *age model RI-only* and *age model RI&RS uncorrelated*.

(ii) We observed low power for the *difference model* across all simulation settings and, empirically, much higher SEs as compared to *age model RI&RS* (**Supplementary Figure 4A**). The loss of power was more pronounced in the UKB-based simulation setting where the average number of eGFR assessments per individual was larger than in the cohort study simulation settings. From theory, we expected large SEs and, consequently, a considerable power disadvantage for the *difference model* (as compared to the other models in data of individuals with ≥2 eGFR assessments) due to its inefficient use of data: it uses only the first and last eGFR assessment of each individual, discarding all assessments in between. Moreover, this power disadvantage was also expected to be more pronounced for datasets with longer trajectories and thus more assessments in between the first and the last one.

(iii) *Age model RI&RS* had slightly higher power than *time model RI&RS* across simulations and in empirical data, as well as smaller SEs empirically (**Supplementary Figure 4B**). In general, *age model RI&RS* is more parsimonious: it estimates the temporal effect via a single age parameter, as opposed to *time model RI&RS*, which separates the temporal effect before and after baseline. This power gain was less pronounced in the cohort study settings.

(iv) The two-stage *BLUPs&LinReg* showed strong power across all simulation scenarios. A bias-variance trade-off was observed: effect estimates were biased towards the null and empirical SEs were substantially reduced compared to *age model RI&RS* (**Supplementary Figure 4E**). Due to their (multivariate) normal distribution, RI and RS terms are shrunk towards zero (L2-regularization), inducing bias in BLUPs towards zero[3,4]. Theory of

regularization suggests that accepting some degree of bias (here: towards the null) in exchange for substantially reduced variance can lead to overall higher power (as compared to the other models in data of individuals with ≥2 eGFR assessments)[5]. Still, the extent of bias in effect estimates (>38% in UKB-based simulations and empirically) was surprising.

(v) We observed slight type I error inflation for *BLUPs&LinReg* in simulations. This is likely due to too low SEs as a result of not carrying over estimation uncertainty of BLUPs into the second-stage linear regression.

(vi) By including individuals with =1 eGFR assessment ("singletons"), *age model RI&RS* achieved a power gain in simulations and has smaller SEs in empirical data (as compared to *age model RI&RS* on data of individuals with ≥2 eGFR assessments; **Supplementary Figure 4F**). The power gain in simulations was substantial in the UKB-based setting (with >50% singletons) and less pronounced in the cohort study settings (with 20% singletons). Thus, as expected, an increase in sample size led to increased power, the extent of which depended on the proportion of singletons.

In summary, we conclude that if we are jointly interested in type I error control, power and unbiased effect estimation, *age model RI&RS* including individuals with =1 eGFR assessment is more suitable than the alternative statistical approaches in all settings we considered.

Supplementary Note 3: Annotation of genes in decline-associated and stable-effect loci

We annotated biological features for the 256 and 182 genes in the 10 decline-associated and 9 stable-associated loci (12 and 11 identified variants), using KidneyGPS[1] (**Methods**; **Supplementary Data 2**). For each of six decline-associated loci, we found a gene supported by ≥3 features as likely causal (*UMOD, PRKAG2, SDCCAG8, RRAGD, TPPP, FGF5*; **Supplementary Data 2**): (i) for each of the respective six loci, we found at least one variant (index variant or variant around it as part of 95% credible set) that was statistically highly likely to drive the association (i.e., posterior probability of association ≥10%); in fact, the index variant was the most likely causal variant for these six loci (posterior probability=100%, 80%, 22%, 93%, 90%, 49%, respectively); (ii) for all six genes, the gene contained the index variant or was the nearest gene to the index variant, which was shown to increase the probability of a gene to be causal[6]; (iii) four of the six genes were known for Mendelian kidney disease by rare variants (*UMOD, PRKAG2, SDCCAG8, RRAGD:* autosomal dominant tubulo-interstitial kidney disease (ADTKD), renomegaly, retina-renal ciliopathy, or renal hypomagnesemia, respectively); (iv) two of these four genes were further supported by the index variant modifying gene expression in kidney tissue (*UMOD)* or potentially modifying protein abundance (5’ UTR of *RRAGD*); (v) further two index variants modified gene expression in kidney tissue, but without a known human kidney phenotype *(TPPP, FGF5).*

SLC9A3 was an alternative gene in the *TPPP* locus, encoding the sodium/hydrogen exporter isoform 3 (NHE3) expressed in kidney and intestine and an FDA approved drug for CKD patients. Thus, this would be a compelling proof-of-concept that decline-associated loci capture drug targets for CKD patients. However, *SLC9A3* was not supported by any further features, and TPPP was the more likely the causal gene of that signal (see above).

For four of the 9 stable-effect loci, we found again a gene supported as likely causal (*CPS1, SLC22A2, SLC34A1*, and *UNCX*): (i) for the four loci, the index variant was the most likely causal variant for the association and had a high probability (posterior probability=100%, 43%, 100%, 61%, respectively), (ii) the index variant resided in the gene, (iii) the gene had a role in creatinine production[7,8] (*CPS1*) and the index variant was protein-altering (“missense”) or the gene had a known role for tubular reuptake of creatinine[7] (*SLC22A2*) or the gene was known for Mendelian kidney disease (*SLC34A1*), or the index variant modulated gene expression in kidney tissue (*UNCX*).

We also hypothesized that the 10 decline-associated loci differentiated pathways compared to the 9 stable-effect loci. We thus analyzed the genes in the 10 versus the 9 loci separately for enriched pathway (Reactome pathways and Protein classes with PANTHER 18.0[9,10]). We found “antimicrobial peptides” enriched among the genes in decline-associated loci (corrected $P_{pathway}$=1.28x10^{-3}, 9.65-fold enrichment; *DEFB130A, DEFB136, BPIFA1,*

DEFB135, BPIFA2, BPIFB4, DEFB134, DEFB109B, BPIFB1). However, the genes in this pathway were located only in two loci (near *PRAG1* and *GGT7*) and thus not considered as informative.

Supplementary Figures

Supplementary Figure 1: Inclusion and exclusion of UKB individuals for analyses.
We started with the UKB data for eGFR trajectories integrating creatinine values from study center and eHR from "GP-clinical" as described previously[11] (2,102,174 serum creatinine measurements from 454,907 individuals after exclusion of implausible values and duplicates). We included unrelated UKB individuals of European ancestry without recorded acute kidney injury (AKI) or nephrectomy at any timepoint. We excluded eGFR-values at and after onset of renal replacement therapy (dialysis, kidney transplantation) or otherwise recorded end-stage kidney disease (ESKD), or after an observed eGFR-value <15 mL/min/1.73m². Our final UKB 350K data yielded n=348,275 individuals and m=1,520,382 eGFR assessments (199,012 individuals with =1 eGFR assessment; 149,263 individuals with ≥2eGFR assessment). Since some statistical approaches require ≥2 assessments, we also derived the UKB 150K data (n=149,263, m=1,321,370).

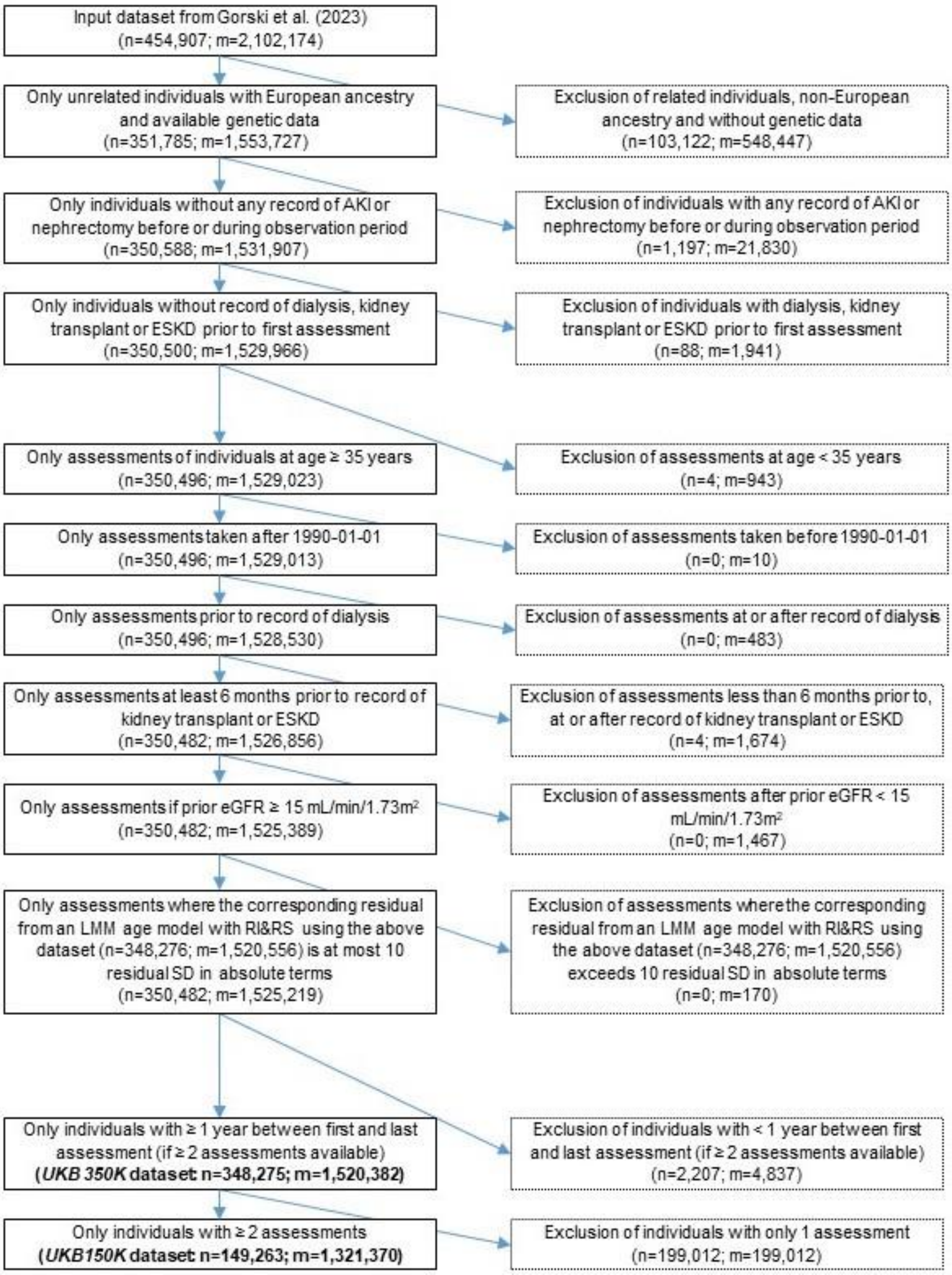

Supplementary Figure 2: Relationship of eGFR with age and time allowing for non-linearity.

We show eGFR modelled over time or age by sex allowing for non-linear relationship (penalized splines by sex, using *gam()*, **R** package *mgcv*[12]). **a** Modeling eGFR as function of time adjusting for age at 1st eGFR assessment, with random intercepts (RI) and random slopes (RS), using the UKB 150K data (n=149,263, m=1,321,370). **b** Equivalent to **a**, but modeling eGFR as function of age-at-exam. **c** Equivalent to **b**, but using UKB 350K (i.e. adding individuals with =1 eGFR assessment; n=348,275, m=1,520,382). **d** Equivalent to **a**, but restricted to individuals with CKD (i.e. individuals with eGFR<60 mL/min/1.73m² for at least one timepoint, excluding eGFR-values before this timepoint; n=13,116, m=116,944); first 3 years are shaded in grey due to likely regression-to-the-mean at the 2nd timepoint in these trajectories (median time difference between 1st and 2nd eGFR assessment ~3 years).

a Effect of time on eGFR (UKB 150K)

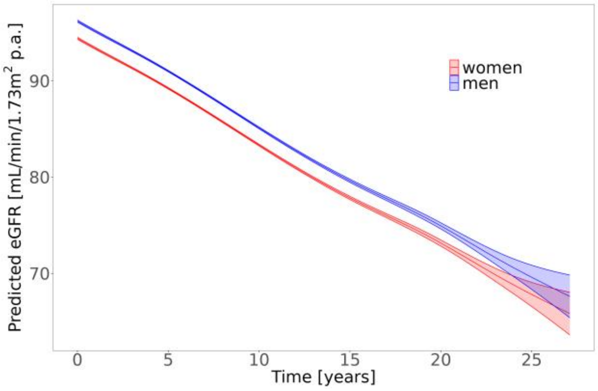

b Effect of age on eGFR (UKB 150K)

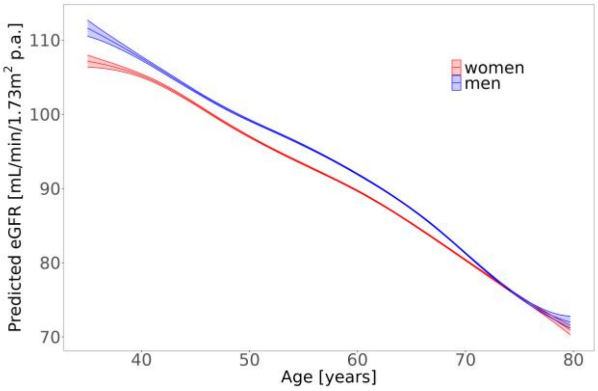

c Effect of age on eGFR (UKB 350K)

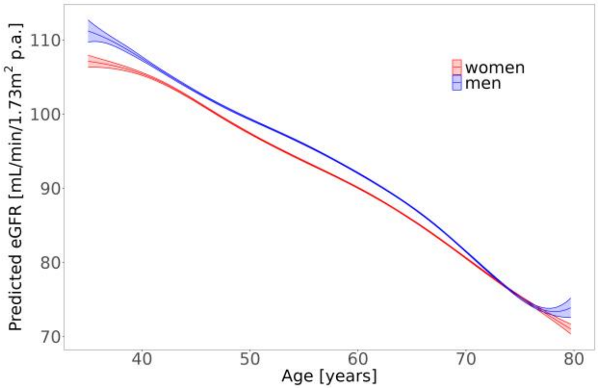

d Effect of time on eGFR in CKD (UKB 150K)

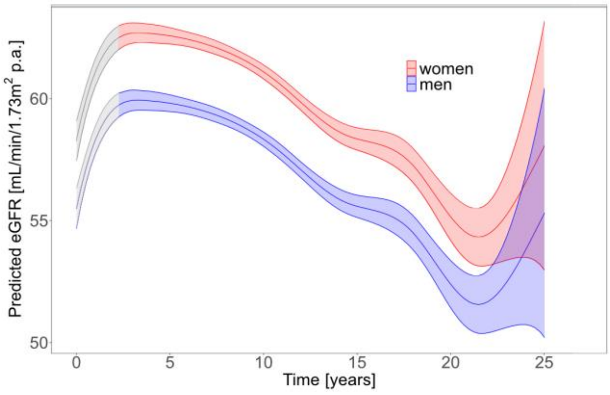

Supplementary Figure 3: Visualization of permutation-based type I error via QQ plot across all seven approaches.

We show observed versus expected P-values of association with eGFR-decline across all seven approaches, for 10,000 randomly permuted genotypes across UKB eGFR trajectories. The seven approaches are color coded: *difference model* (black), *time model RI&RS* (purple), *age model RI&RS* (magenta), *age model RI&RS uncorrelated* (green), *age model RI-only* (blue), *BLUPs&LinReg* (red), *age model RI&RS 350K* (orange). Accumulated observed P-values above the identity line indicate type I error inflation (observed for blue and green). Source data are provided as a Source Data file.

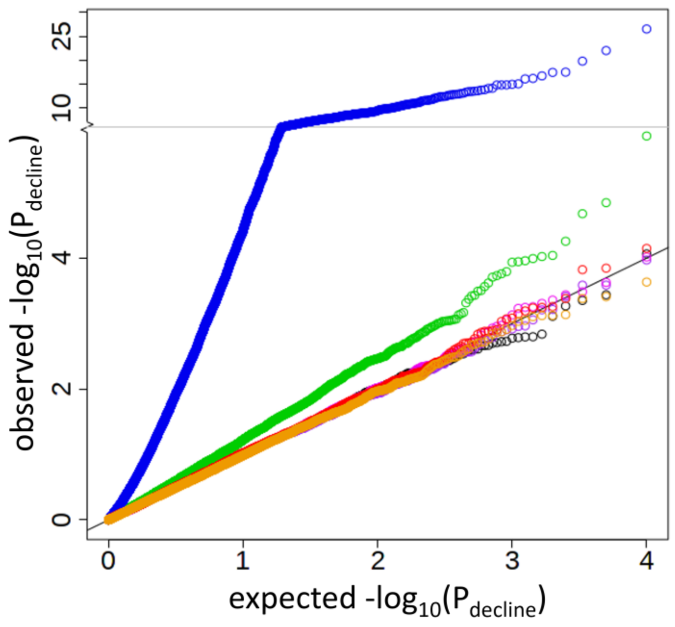

Supplementary Figure 4: Comparison of genetic effect estimates and standard errors for eGFR-decline between approaches.

We analyzed 595 SNPs for association with eGFR-decline using the seven approaches. Each row shows pairwise comparisons of effect estimates (left panels) and standard errors (SEs; right panels) for six alternative approaches (y-axis) against the *age model RI&RS* applied to UKB 150K: **a** *difference model*, **b** *time model RI&RS*, **c** *age model RI&RS uncorrelated*, **d** *age model RI-only*, **e** *BLUPs&LinReg*, all using UKB 150K; **f** *age model RI&RS 350K* using UKB 350K. The units for effect estimates and SEs are mL/min/1.73m^2 per allele and year. The 12 variants identified at Bonferroni(595)-corrected significance using the *age model RI&RS 350K* ($P_{decline}$ <0.05/595=8.4x10^{-5}) are color-coded (green: known variant for eGFR-decline; blue: novel variant); also color-coded are two known variants for eGFR-decline not identified here (orange) and three variants known for not being associated with eGFR-decline (red)[13]. Source data are provided as part of **Supplementary Data 1**.

a Difference model

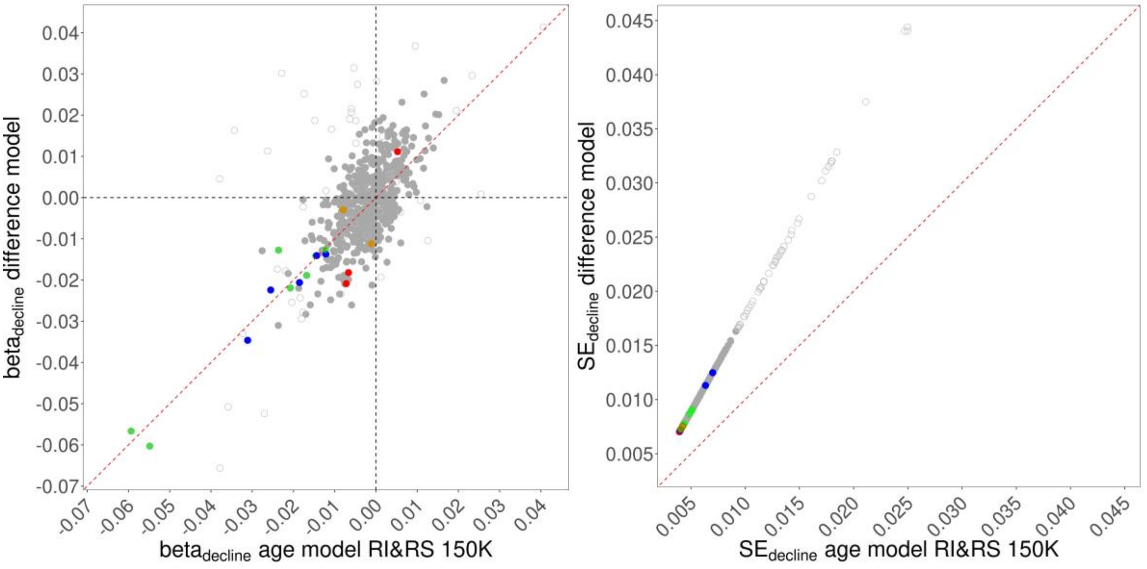

b Time model RI&RS

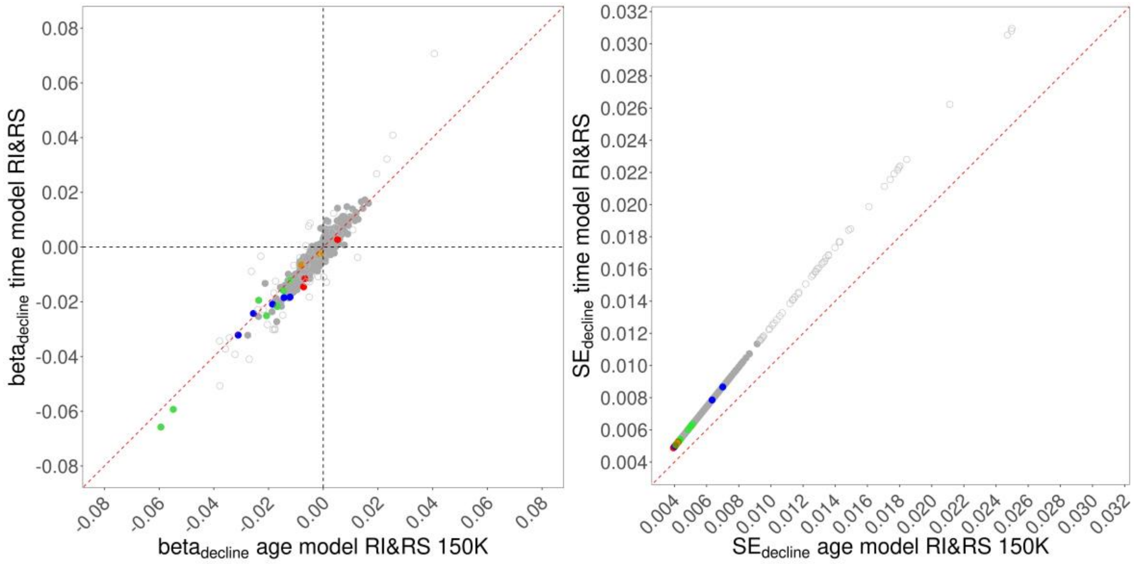

c Age model RI&RS uncorrelated

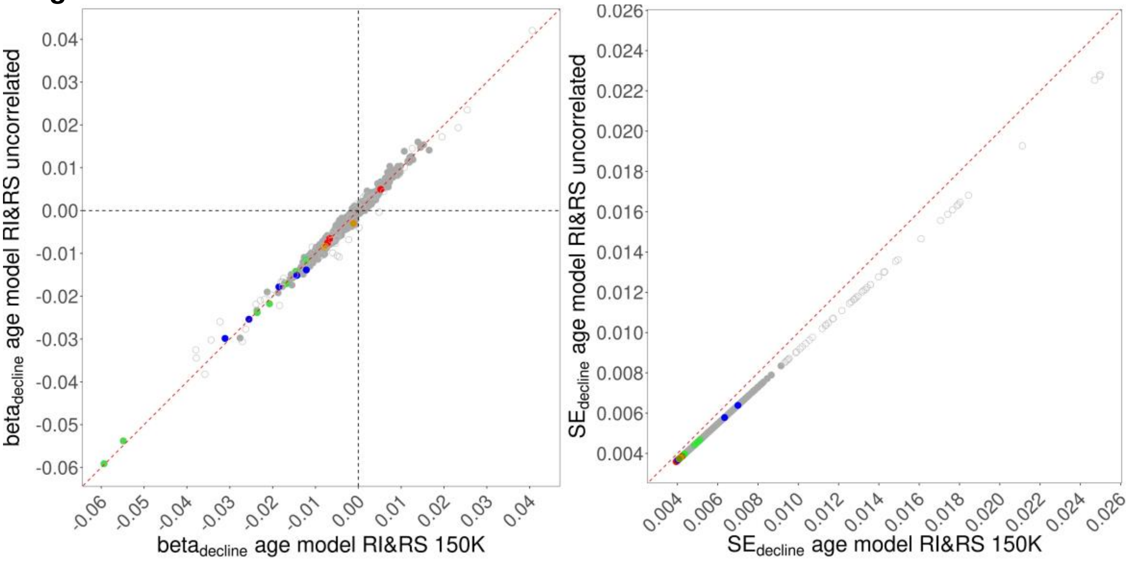

d Age model RI-only

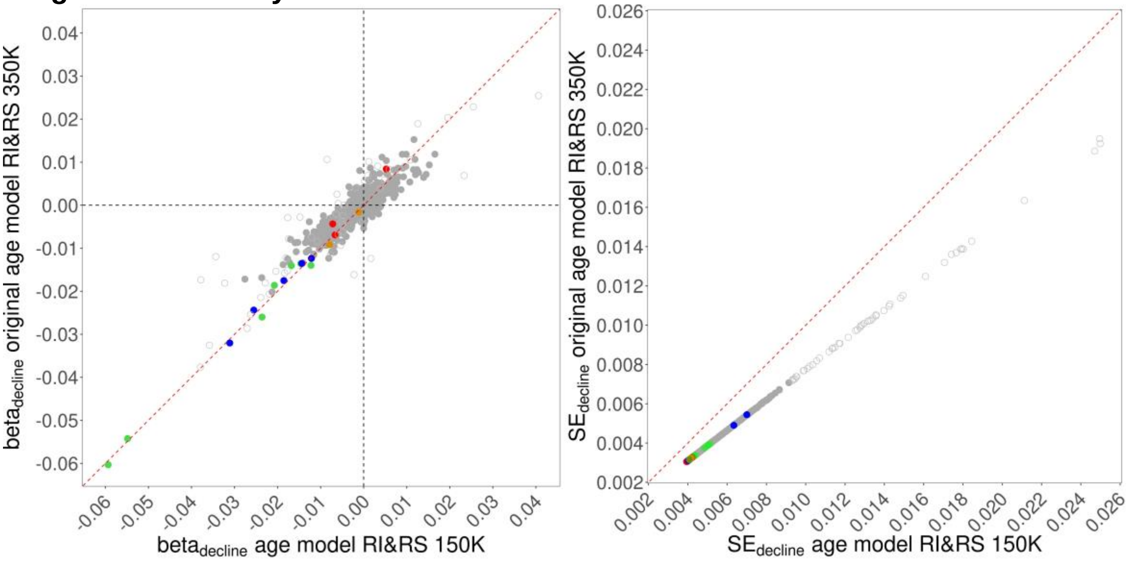

e BLUPs&LinReg

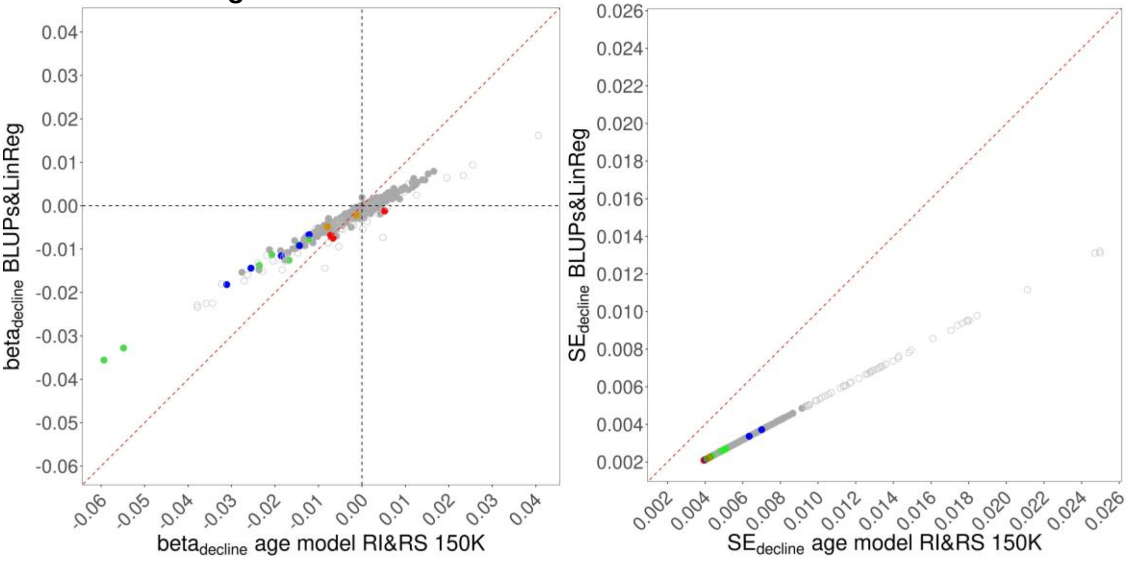

f Age model RI&RS 350K

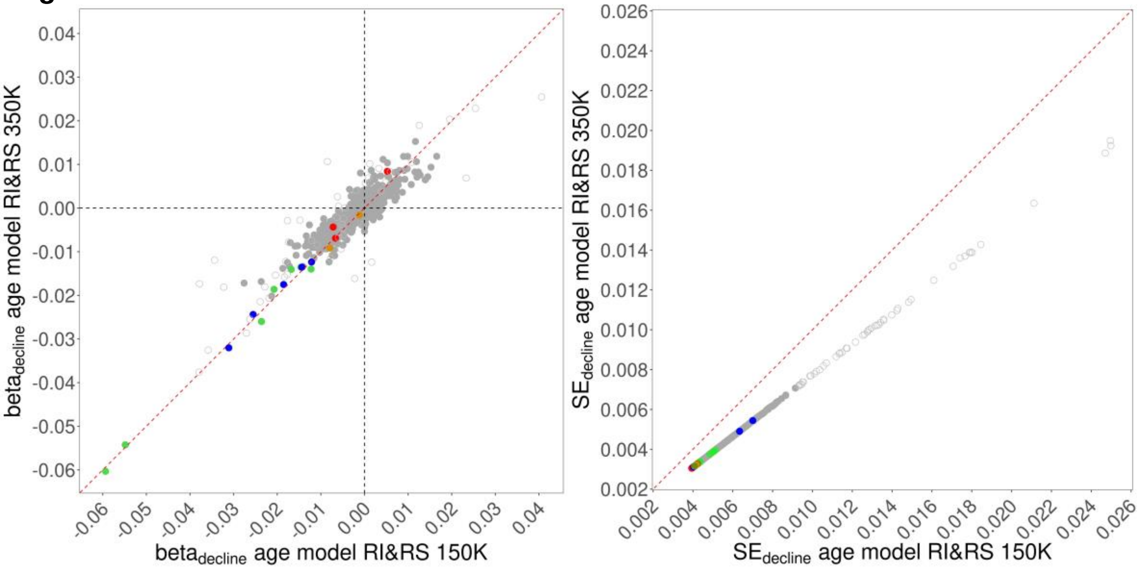

Supplementary Figure 5: Sensitivity analyses for the LMM age model RI&RS 350K.
For the analyses of the 595 SNPs based on the LMM *age model RI&RS* 350K (UKB 350K; n=348,275, m=1,520,382), we performed seven sensitivity analyses. Each row shows pairwise comparisons of effect estimates for eGFR-decline (left panels) and standard errors (SEs; right panels) from the original analysis (i.e., adjusted for sex and 20 PCs) against sensitivity analyses. **a** Adding age-by-sex interaction, **b** adding age-by-birthyear interaction, **c** adjusting for source of serum creatinine value as study center or eHR, **d** excluding extreme observations, potentially due to technical artefacts (|residual Z-score|>4 from model without SNP as covariate), **e** excluding individuals with extreme random slopes, potentially due to technical artefacts or extreme disease course (|RS|>4 RS SD from model without SNP as covariate), **f** excluding individuals with long trajectories (>50 eGFR assessments) potentially indicative of severe disease course resulting in abundance of eHR entries, or **g** excluding individuals with any record of dialysis, kidney transplant or ESKD. The units for effect estimates and SEs are mL/min/1.73m^2 p.a. The color-code is identical to **Supplementary Figure 4**. Source data are provided as a Source Data file.

a Age model RI&RS 350K adding age-by-sex interaction

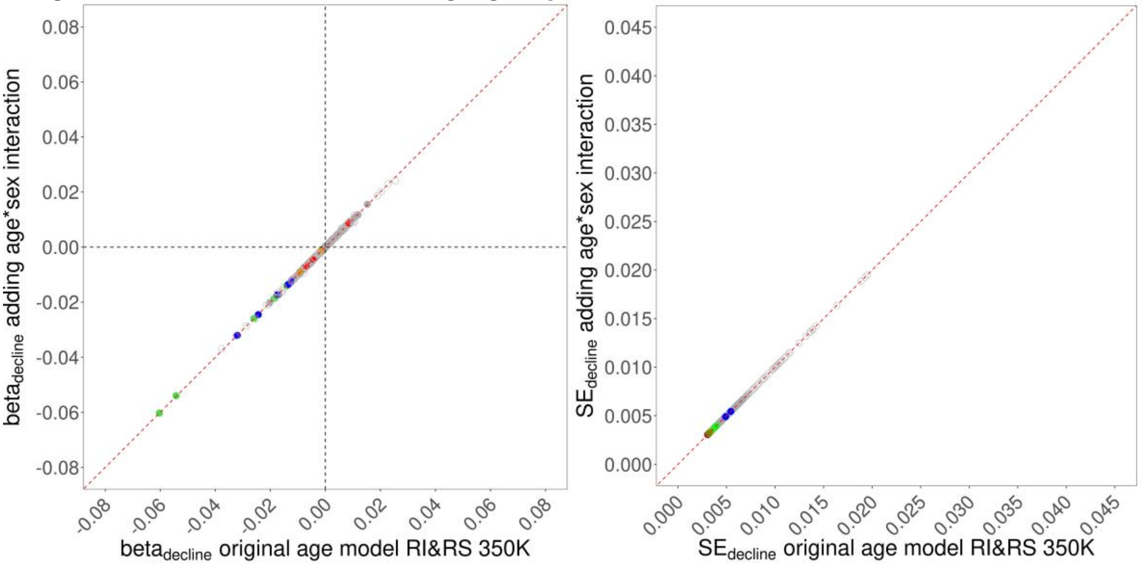

b Age model RI&RS 350K adding age-by-birthyear interaction

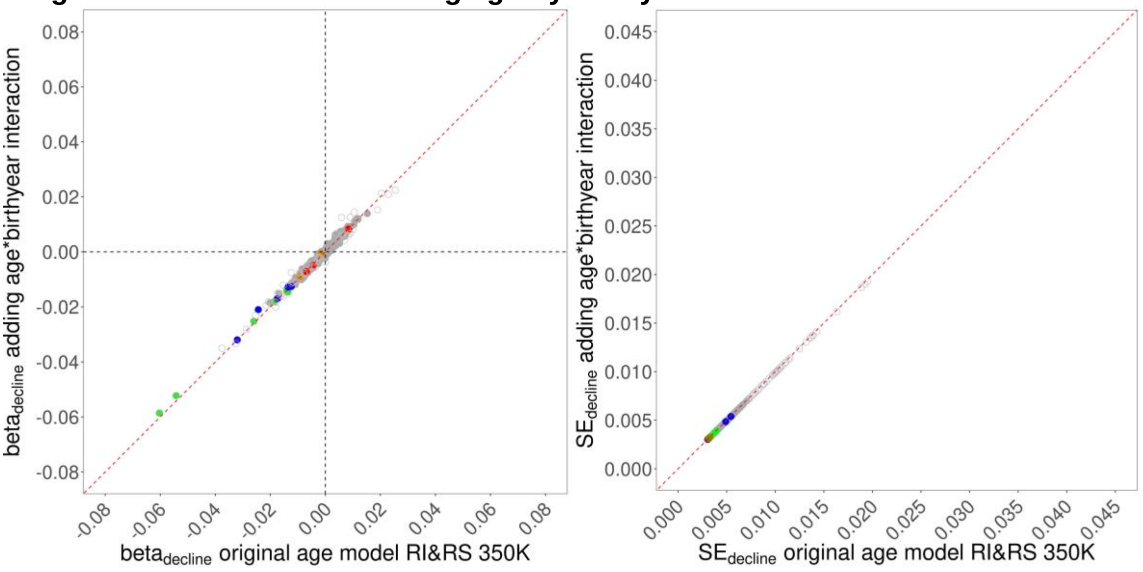

c Age model RI&RS 350K adding source of serum creatinine value

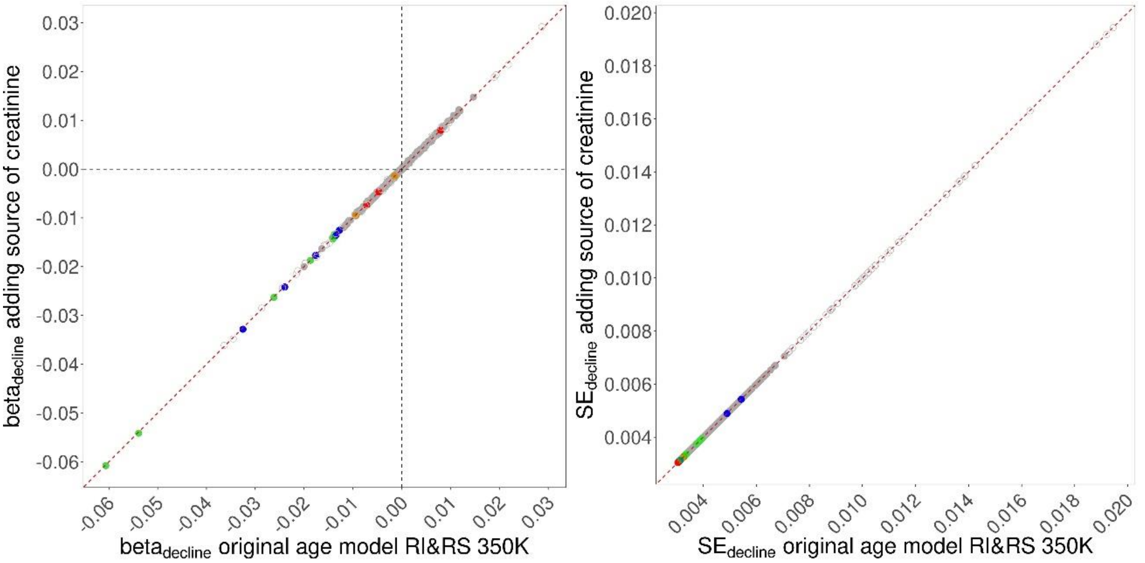

d Age model RI&RS 350K excluding extreme observations

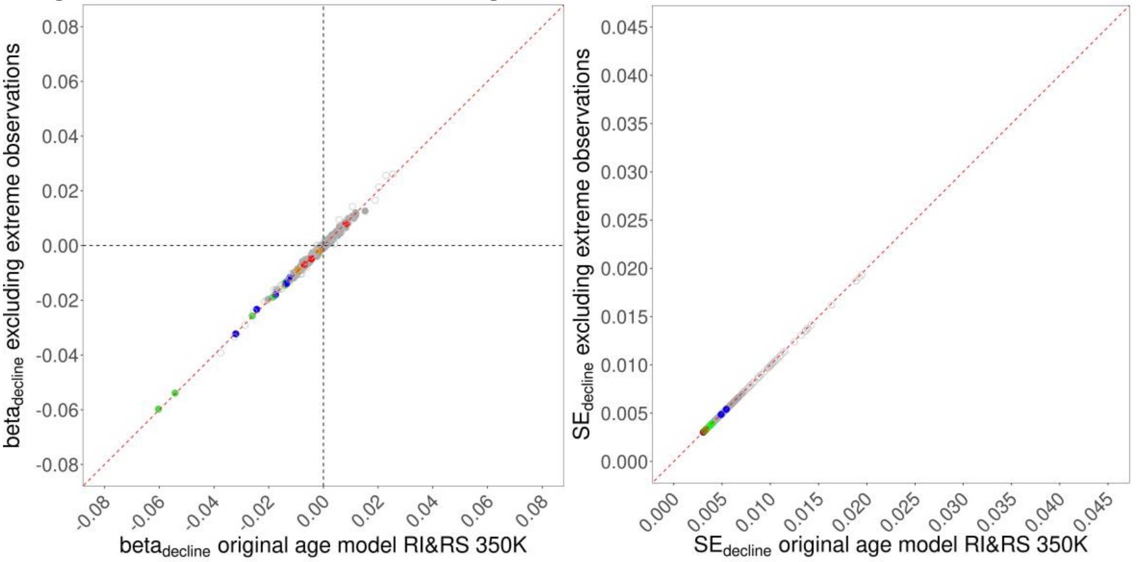

e Age model RI&RS 350K excluding extreme slopes

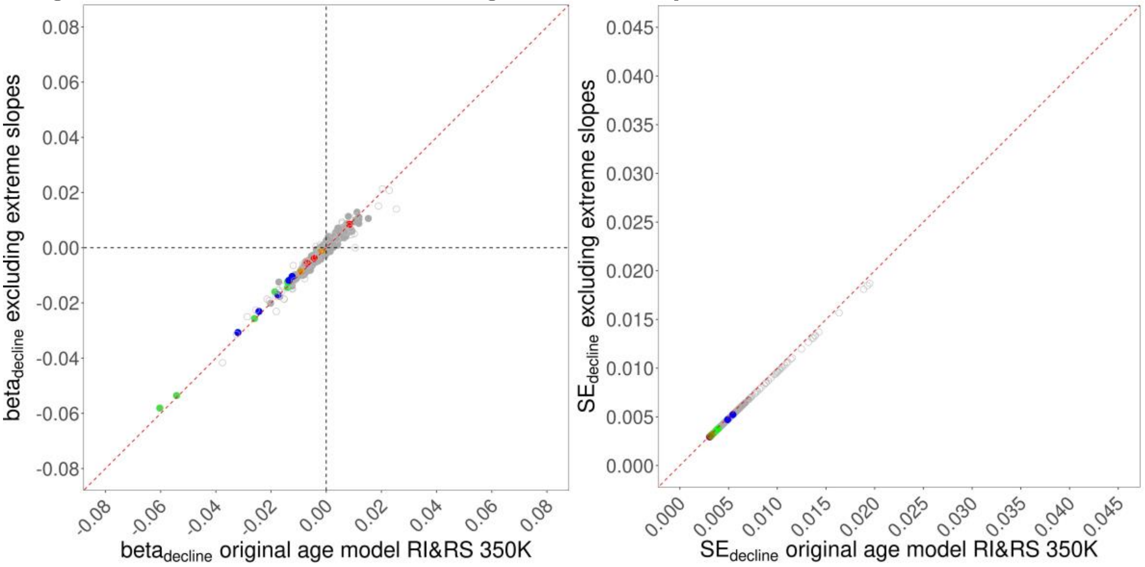

f Age model RI&RS 350K excluding long trajectories

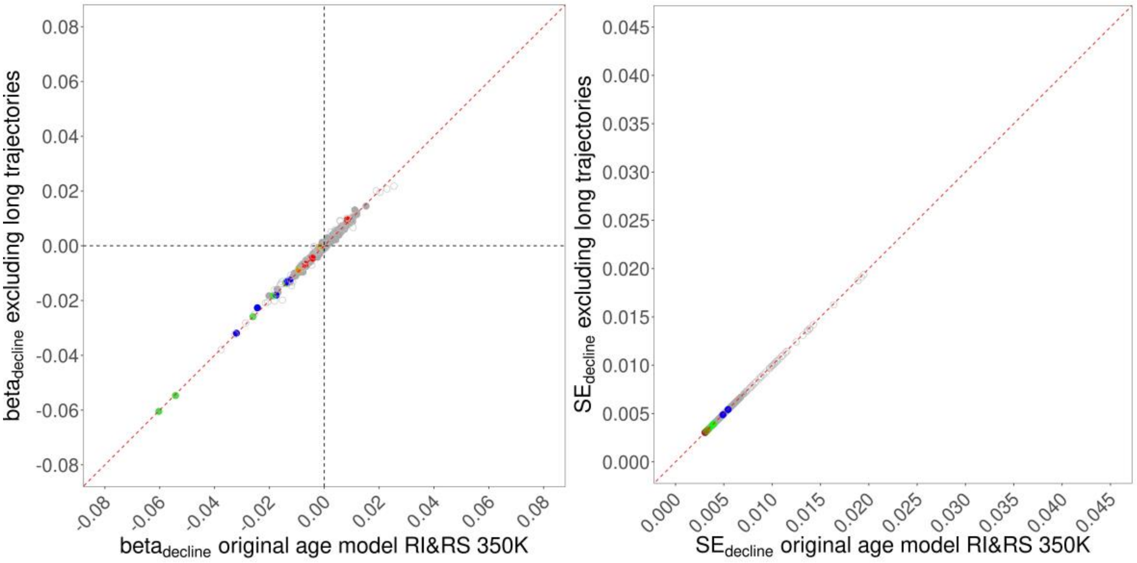

g Age model RI&RS 350K excluding individuals with any record of dialysis, kidney transplant or ESKD

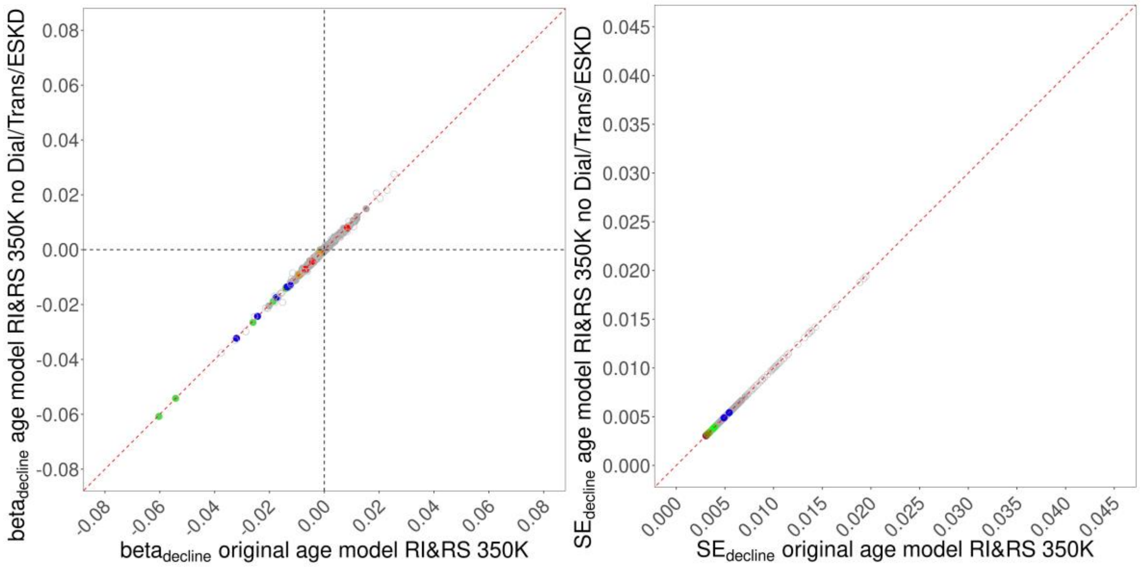

Supplementary Figure 6: SNP-association for eGFR-decline under non-linear modeling of age effects.

For the 12 decline-associated and 11 stable-effect variants, we evaluated the impact of non-linear modeling of age effects on SNP-associations for eGFR-decline. Each row shows pairwise comparisons of SNP effect estimates for eGFR-decline ($beta_{SNPxage}$; left panels) and standard errors (SEs; right panels) from the original analysis (LMM *age model RI&RS 350K*; $eGFR_t$~age, sex, SNPxage, RI, RS, 20PCs) against the same approach adding quadratic terms (**Methods**): **a** adding age², **b** adding age² and expanding the random effect term by age² (beyond RI and linear RS), **c** adding age² and SNPxage^2 (centered at 59 years here to minimize correlation between age and age^2). Units for betas and SEs are mL/min/1.73m^2 per allele and year. Color code refers to the 12 variants for eGFR-decline (blue: novel for eGFR-decline; green: known for eGFR-decline) or the 11 stable-effect variants (black). Source data are provided as a Source Data file.

a Age model RI&RS 350K adding age²

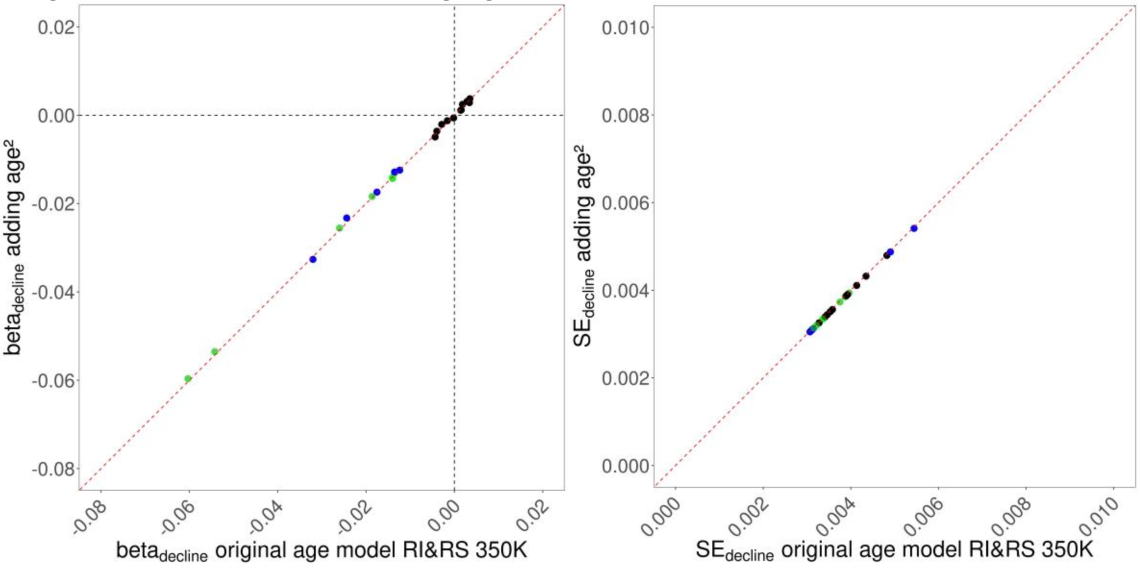

b Age model RI&RS 350K adding age² and including age² into the random effect

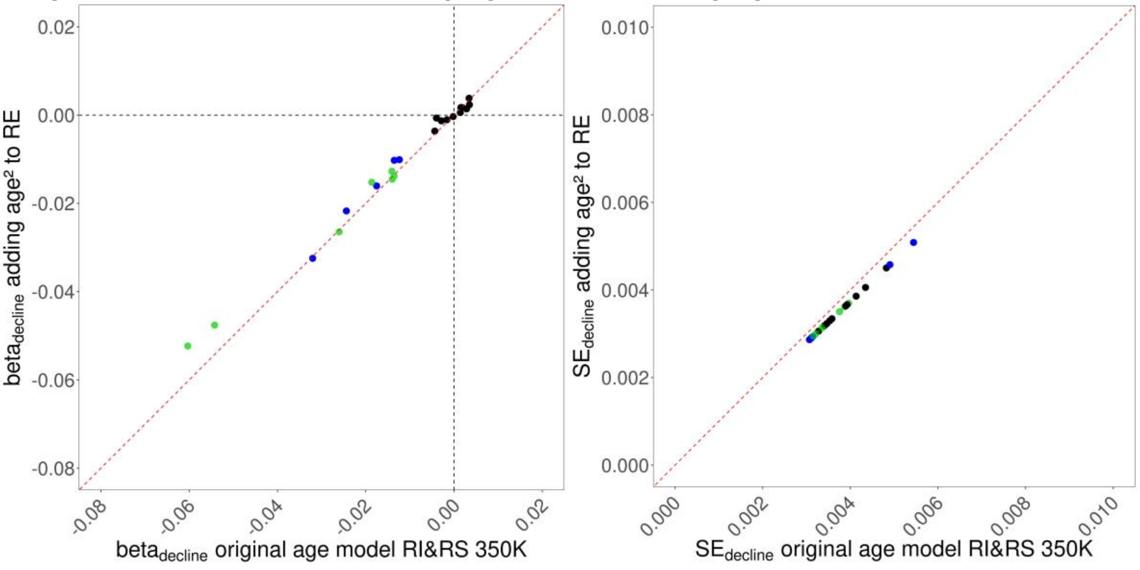

c Age model RI&RS 350K adding age² and SNP*age²

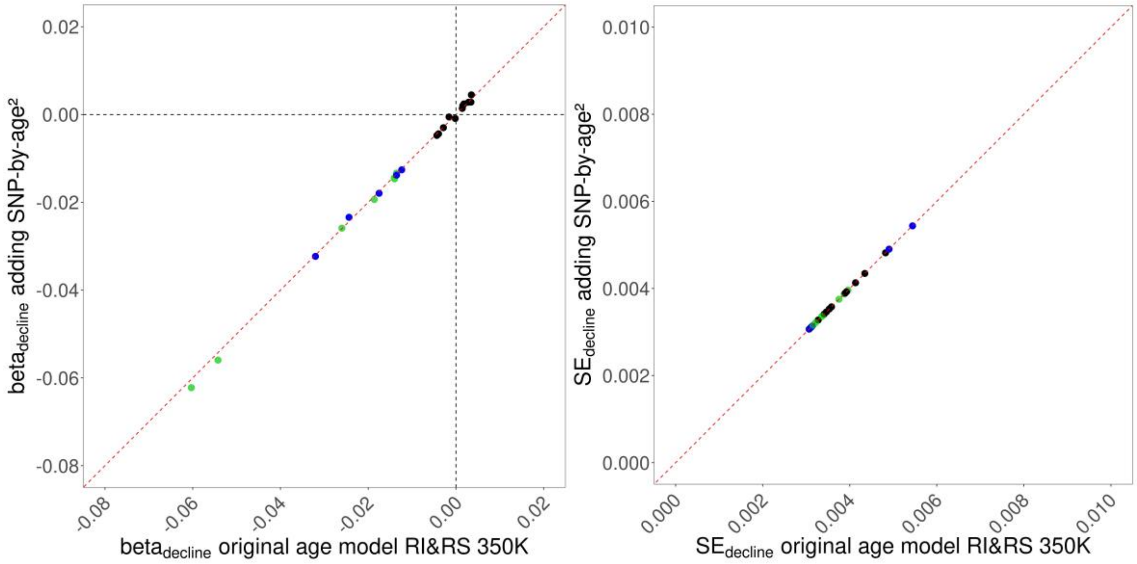

Supplementary Figure 7: The age-dependency of SNP effects on eGFR is approximately linear even under non-linear modeling.

We evaluated potential non-linearity in the age-dependency of SNP effects on eGFR (i.e. SNP effects on eGFR-decline allowing for non-linear decline). We extended the LMM *age model RI&RS 350K* with a global quadratic age effect and a SNPxage^2 interaction. We show SNP effects on eGFR estimated for 40-, 50-, 60-, 70-year-old individuals (beta derived as $beta_{main}$ + (age-50)*$beta_{linear\_decline}$ + (age-50)^2*$beta_{quadratic\_decline}$) for the 12 decline-associated variants (**left**; blue: novel for eGFR-decline, green: known for eGFR-decline) and the 11 stable-effect variants (**right**; black). Only two variants (rs77924615 and rs13334589, both in/near *UMOD/PDILT*) showed a significant SNPxage^2 interaction ($P_{quadratic\_decline}$<0.05/23), but this over-linear effect was small. Effect allele was the cross-sectionally eGFR-lowering allele[1]. Source data are provided as part of **Supplementary Data 4**.

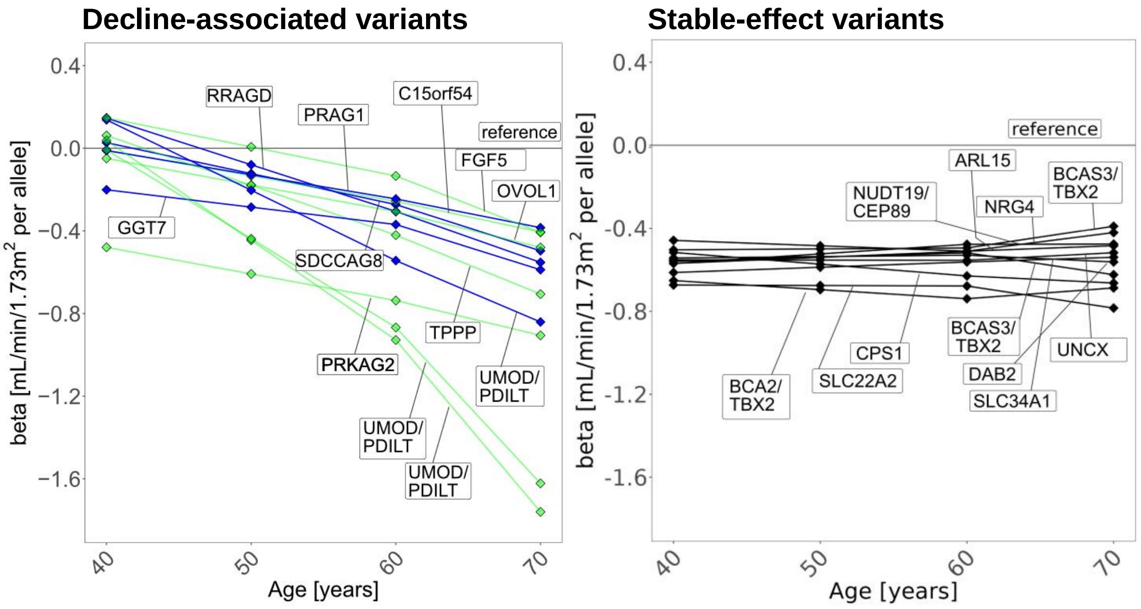

Supplementary Figure 8: SNP effects on eGFR-decline versus SNP effects on eGFR-variability for the 12 decline-associated and 11 stable-effect variants.

We illustrate the relationship between SNP effects on eGFR-decline and SNP effects on eGFR-variability for the 12 decline-associated (novel: blue; known: green) and 11 stable-effect variants (black) in the UKB 350K dataset. Shown are SNP effects on eGFR-decline estimated by LMM *age model RI&RS 350K* (per allele and year) against SNP effects on eGFR-variability (per allele; estimated via a GAMLSS[14]). All 12 decline-associated variants showed significant effects on eGFR-variability ($P_{var}<0.05/23=0.002$). However, 7 of the 11 stable-effect variants also showed significant effects on eGFR-variability ($P_{var}<0.05/23$), implying that genetic effects on eGFR-variability are not specific to eGFR-decline variants. Solid dots indicate $P_{var}<0.05/23$, stars indicate $P_{var}\geq 0.05/23$. Source data are provided as part of **Supplementary Data 4**.

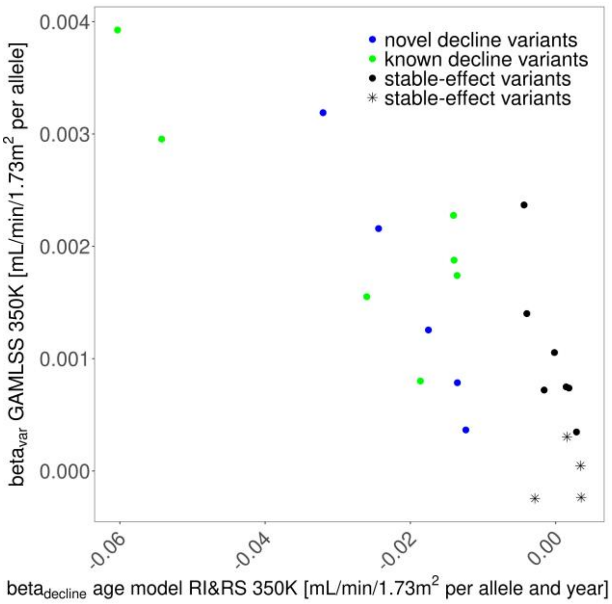

Supplementary Figure 9: Independence of SNP-by-age interaction on eGFR in cross-sectional data upon adjusting for SNP-interaction with diabetes or hypertension.
We conducted SNP-association for eGFR in cross-sectional UKB data (using age and eGFR from baseline study center visits for individuals with available information on diabetes, DM, HbA1c, hypertension, HT, and systolic blood pressure, SBP; n=338,435; linear regression adjusted for sex and 20 PCs). We tested the 12 decline-associated and 11 stable-effect variants for SNPxage interaction on eGFR without and with accounting for SNP interaction with DM, HbA1c, HT, or SBP. DM was defined as antidiabetic medication intake or HbA1c≥6.5%; HT was defined as antihypertensive medication intake or SBP≥140 or diastolic blood pressure ≥90 mmHG. We show SNPxage interaction effect estimates ($beta_{SNPxage}$, x-axis) without versus with adjustment (y-axis) for **a** SNPxDM, **b** SNPxHbA1c, **c** SNPxHT, **d** SNPxSBP (**Supplementary Data 5**). Color codes the 12 decline-associated variants (blue and light blue) and the 11 stable-effect variants (black and gray) with the shade indicating $P_{SNPxage}$<0.05 (blue or black) or ≥0.05 (light blue or gray).

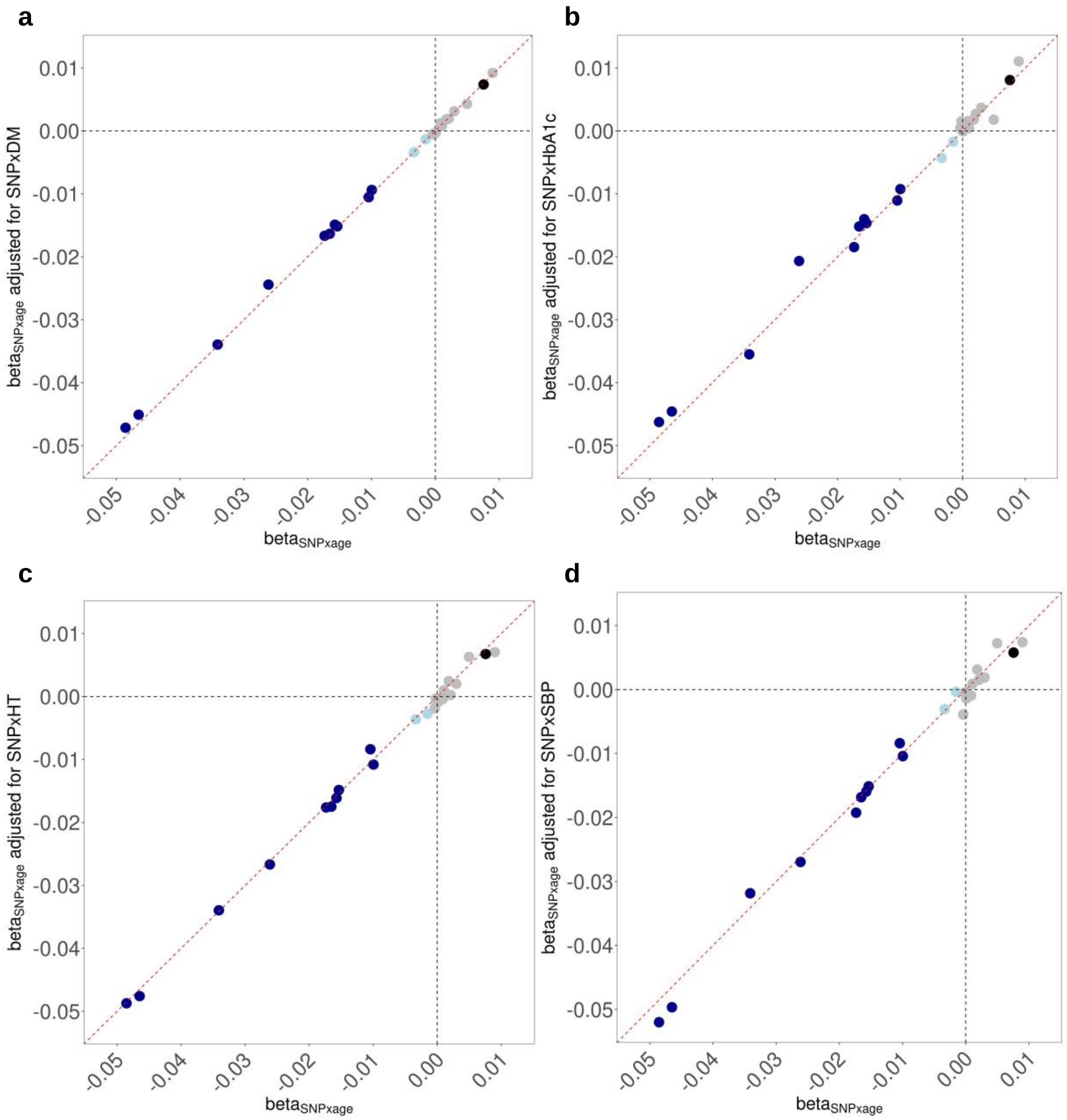

Supplementary Figure 10: Comparison of GMMAT/MAGEE and lme4 implementation of LMM *age model RI&RS 350K* for the 595 SNP-associations with eGFR-decline.

For the 595 SNPs, we show results of their association with eGFR-decline derived from the LMM *age model RI&RS 350K* (UKB 350K; n=348,275, m=1,520,382) using GMMAT/MAGEE[15,16] versus lme4[17]. **a** Genetic effect estimates for eGFR-decline ($beta_{decline}$), **b** corresponding standard errors ($SE_{decline}$), and **c** P-values ($P_{decline}$). $beta_{decline}$, $SE_{decline}$, and $P_{decline}$ are identical for the two implementations, as expected (see also **Supplementary Data 7**).

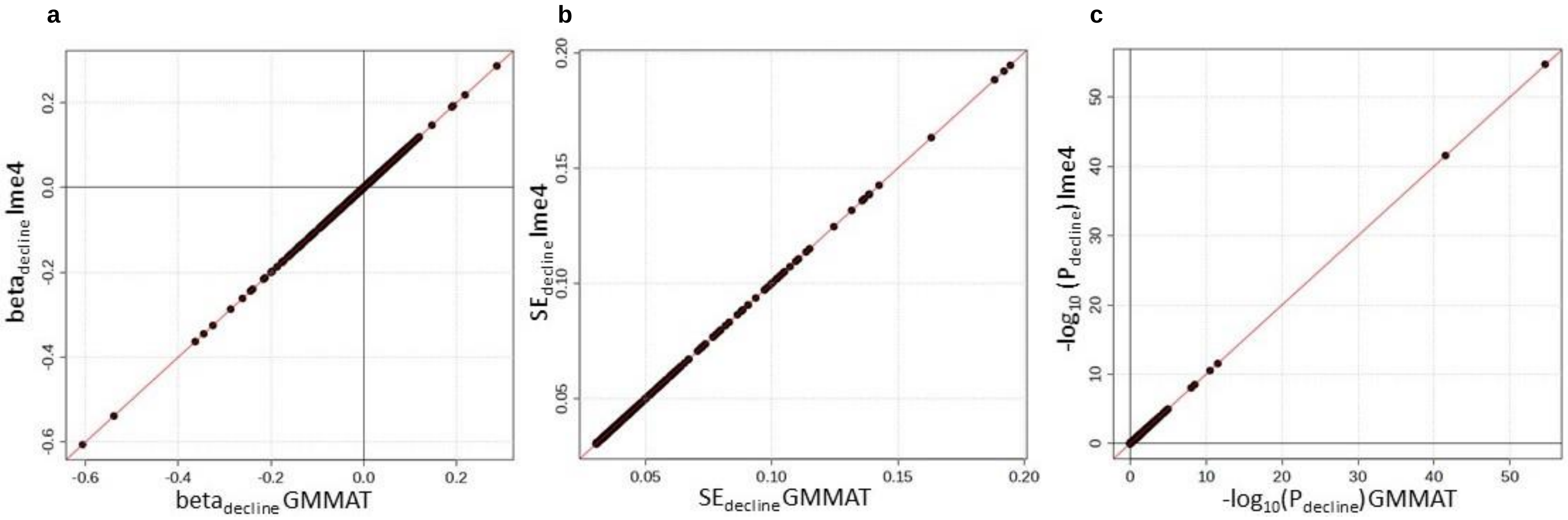

Supplementary Figure 11: Summary of results for the *MTX1/MUC1* locus.

The *MTX1/MUC1* locus was identified for association with eGFR-decline by longGWAS using LMM *age model RI&RS 350K* (UKB 350K; n=348,275, m=1,520,382). The lead variant (rs2075570, $P_{decline}$=1.1x10^{-8}) was not among or correlated with the 595 variants. Among the 595 variants were two variants of that locus not associated with eGFR-decline (rs180921974 and rs4971092; $P_{decline}$=1.49x10-3 and 0.15, respectively), yielding the locus undetected by our 595-search. rs2075570 was not associated with eGFR cross-sectionally ($P_{cross\text{-}sectional}$=0.80, using eGFR from UKB baseline study center visits, n=341,073). We found a similar pattern in models including all three variants (conditional $P_{cross\text{-}sectional}$=2.49x$10^{-12}$, 1.98x$10^{-6}$, 0.18 and conditional $P_{decline}$=0.023, 0.218 and 1.14x10^{-7}, for rs180921974, rs4971092, rs2075570, respectively). **a** We illustrate regional associations ($P_{decline}$, top, and $P_{cross\text{-}sectional}$, bottom). Between-variant r² is colored to rs2075570 (based on 20,000 unrelated UKB individuals of European ancestry). **b** We illustrate linkage disequilibrium (r² and D', using LDlink[18] with European 1000 Genomes reference data) between rs2075570 (EAF=0.51) and rs180921974 and rs4971092 (EAF=0.88 and 0.02, respectively). **c** The rs2075570 association with eGFR shows a complex dependency on age (breakpoint analysis, modeling stepwise linear decline, 95% CIs whiskers).

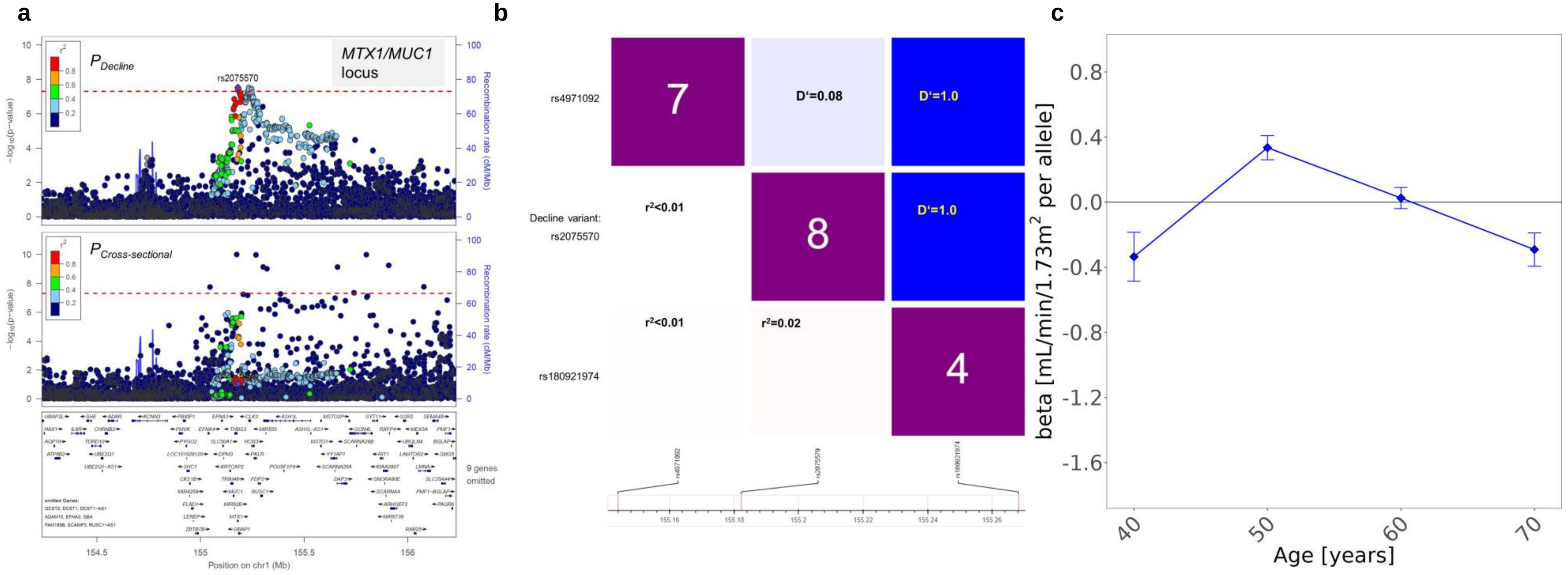

Supplementary Figure 12: Regional association for loci of 12 decline-associated variants.
For the 12 variants in 10 loci identified for association with eGFR-decline, we illustrate regional P-values for eGFR-decline ($P_{decline}$) derived from LMM *age model RI&RS 350K* (UKB 350K; n=348,275, m=1,520,382). We also show P-values for association with eGFR in cross-sectional UKB data analyses ($P_{Cross\text{-}sectional}$; using eGFR from baseline study center visits, n=341,073) versus chromosomal position[19]. Between-variant LD is colored to the lead variant (i.e., most significant decline-associated variant) in this region and computed based on 20,000 unrelated UKB individuals of European ancestry. Regional association plots are shown for: five known loci (**a** *PRKAG2*, **b** *TPPP*, **c** *C15orf54*, **d** *FGF5*, and **e** *OVOL1*); the well-known *UMOD/PDILT* locus (**f&g** two known signals for eGFR-decline; **h** novel signal for eGFR-decline); and four novel loci (**i** *SDCCAG8*, **j** *RRAGD*, **k** *PRAG1*, and **l** *GGT7*). For the *C15orf54* locus in **c** and the *RRAGD* locus in **j**, the decline-associated variants captured a 2nd signal from the cross-sectional analyses[20].

a

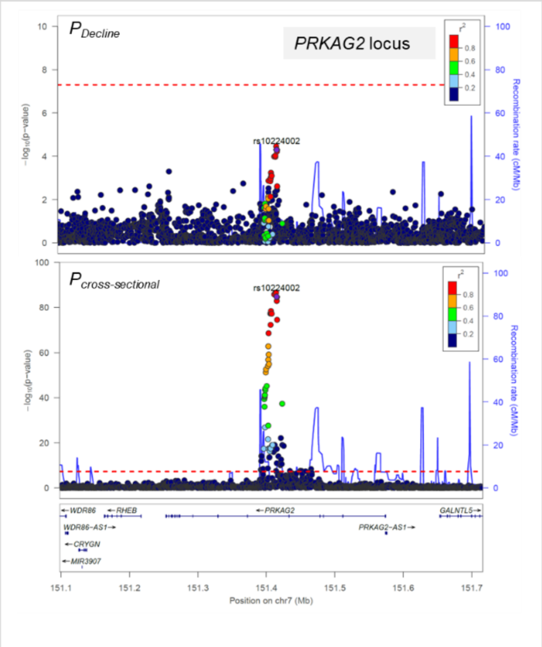

b

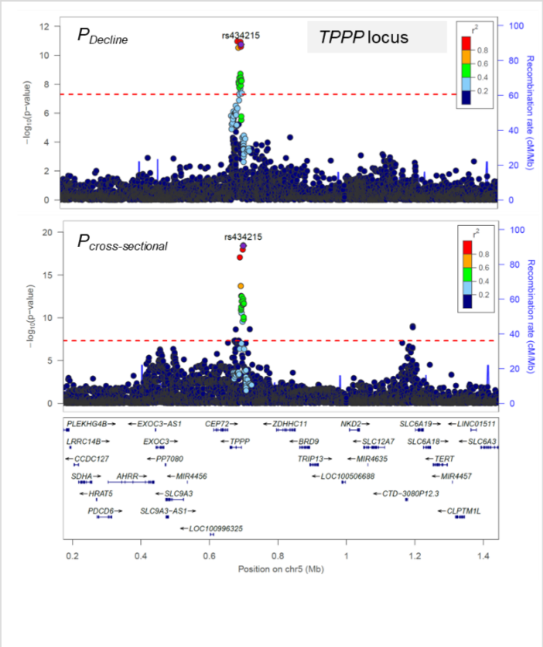

c

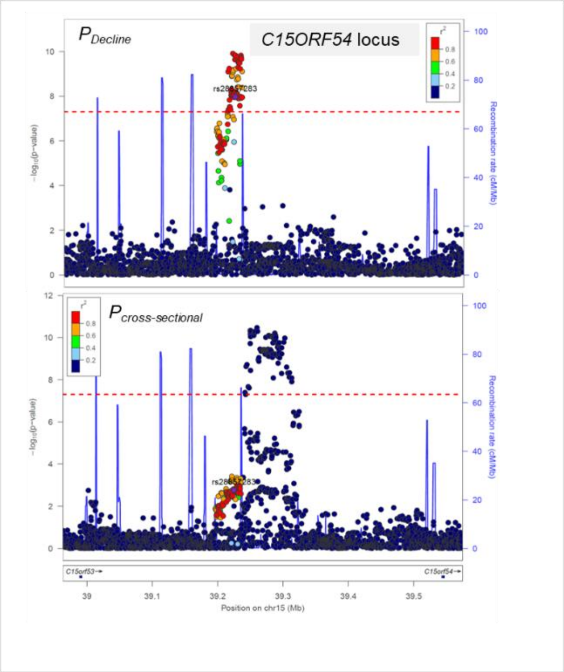

d

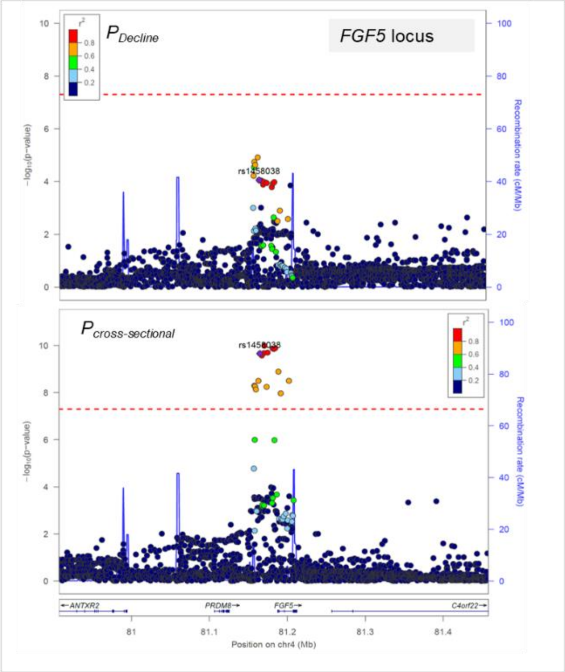

Supplementary Figure 12 (continued)

e

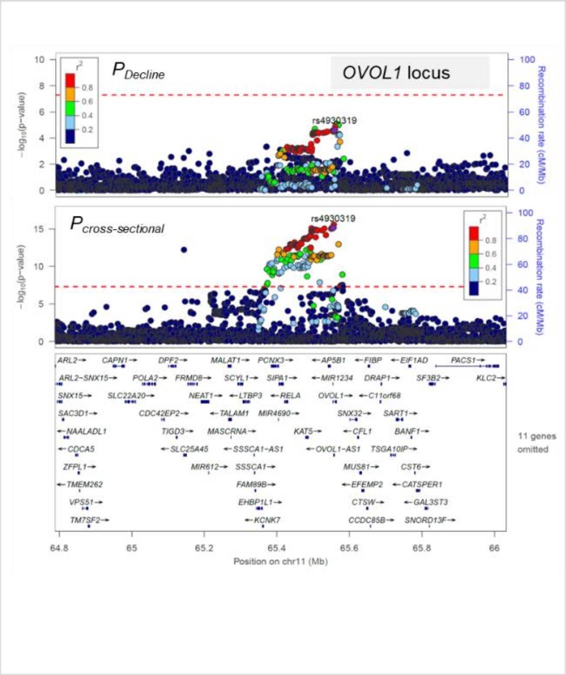

f

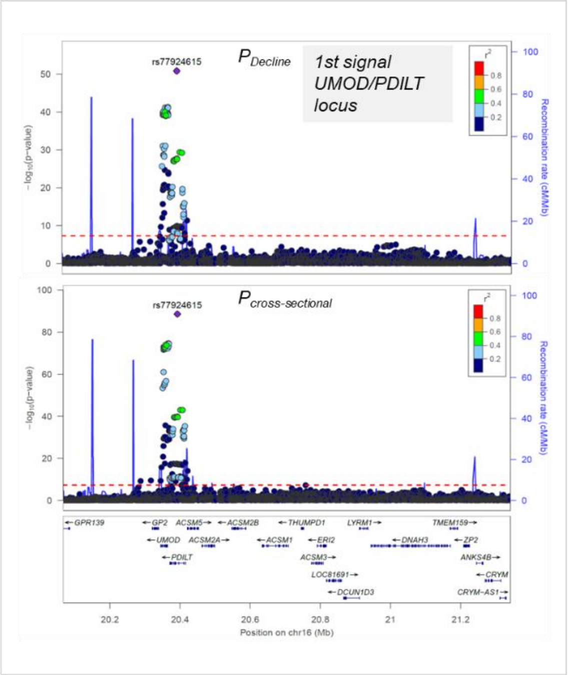

g

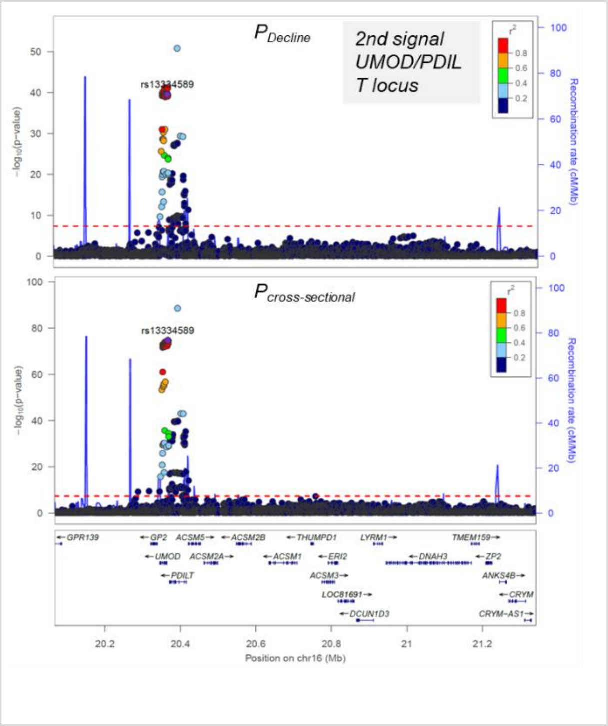

h

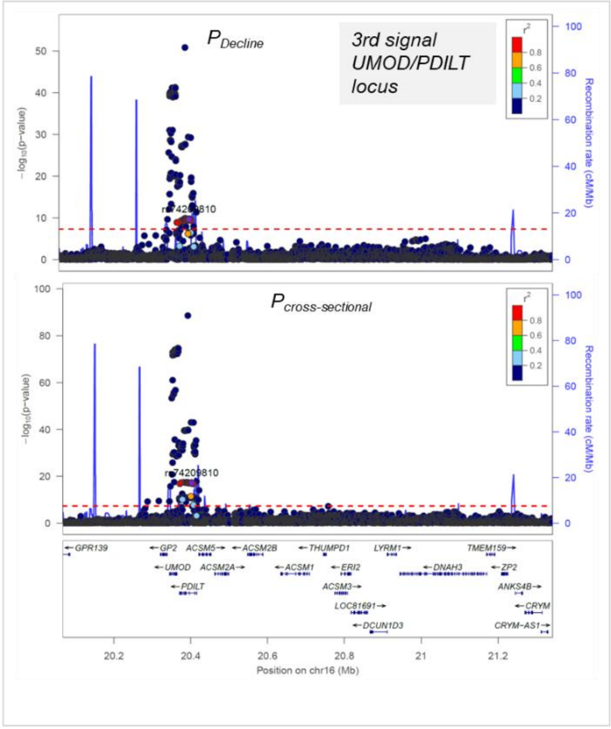

Supplementary Figure 12 (continued)

i

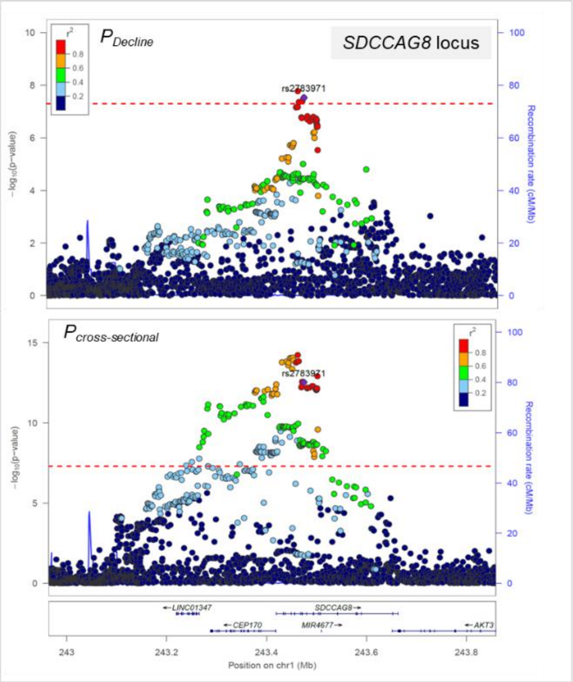

j

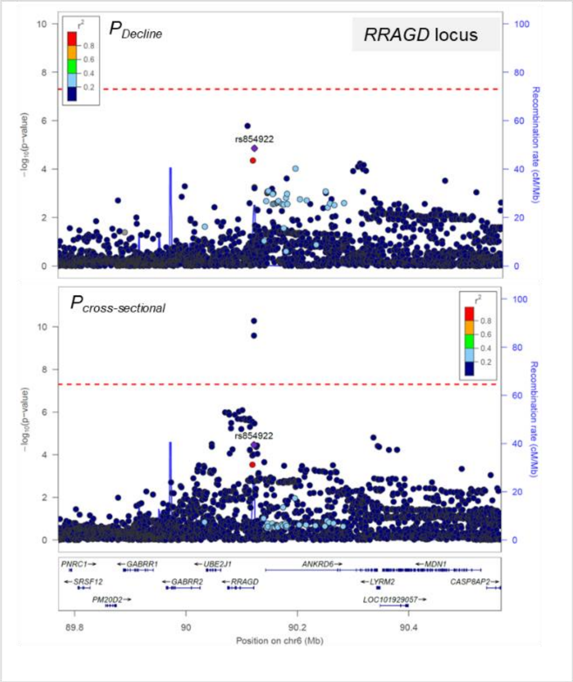

k

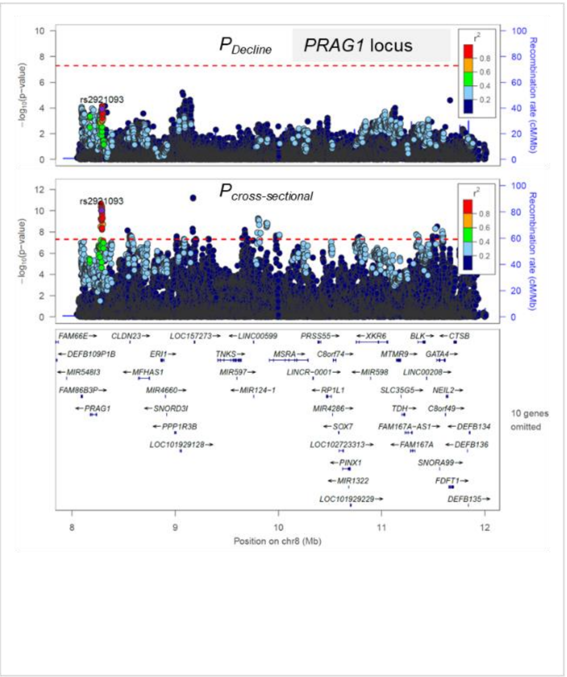

l

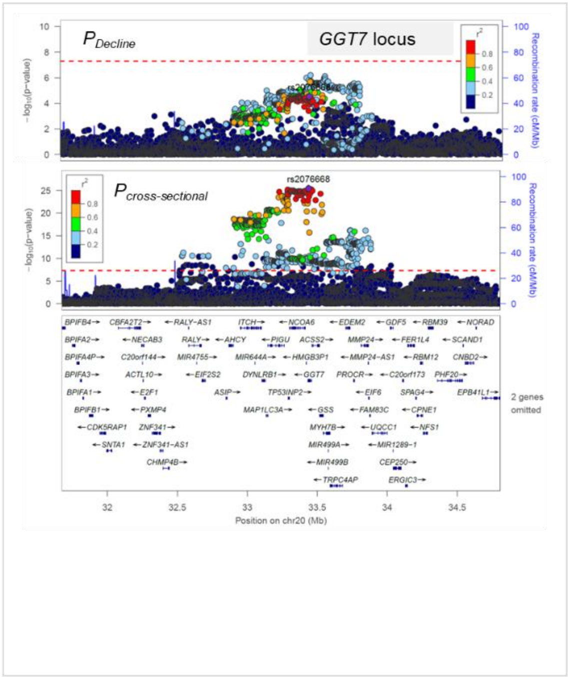

Supplementary Tables

Supplementary Table 1: Annual eGFR-decline estimates across approaches without genetics.

We applied LMMs modeling eGFR (in mL/min/1.73m^2) over age or time adjusted for sex without SNP as covariate ("phenotypic model"), with random intercepts, random slopes, and their correlation (*RI&RS*) if not indicated otherwise: *time model RI&*RS (eGFR as function of time-since-baseline, additionally adjusted for age-at-baseline), *age model RI&RS* (eGFR as function of age-at-exam), *age model RI&RS uncorrelated* (assuming uncorrelated RI and RS), *age model RI-only* (without RS term). We used UKB 150K (n=149,263; m=1,321,370) and additionally, for *age model RI&RS*, UKB 350K (*age model RI&RS 350K*; n=348,275; m=1,520,382). We show covariate effect estimates and 95%-CIs, as well as SD of RS where applicable. "Intercept" estimates average eGFR for individuals at age 50 years, the coefficients for "time" or "age-at-exam" estimate the global annual eGFR-decline.

**Phenotypic model**		**beta**	**SE**	**CI**
Difference model	Does not model eGFR over age or time			
Time model RI&RS (RS SD: 0.951)	Intercept	99.00	0.04	(98.92; 99. 07)
	Age-at-baseline	-0.71	0.003	(-0.72; -0.70)
	Time	-1.08	0.003	(-1.08; -1.07)
	Sex	1.95	0.05	(1.85; 2.05)
Age model RI&RS (RS SD: 0.783)	Intercept	99.94	0.04	(99.86; 100.03)
	Age-at-exam	-0.97	0.003	(-0.97; -0.96)
	Sex	2.27	0.06	(2.16; 2.38)
Age model RI&RS uncorrelated (RS SD: 0.663)	Intercept	99.58	0.04	(99.50; 99.67)
	Age-at-exam	-0.97	0.003	(-0.98; -0.97)
	Sex	2.62	0.06	(2.50; 2.73)
Age model RI-only	Intercept	100.35	0.04	(100.27; 100.43)
	Age-at-exam	-1.02	0.002	(-1.02; -1.01)
	Sex	1.78	0.05	(1.68; 1.89)
BLUPs&LinReg (first stage)	Identical to age model RI&RS			
Age model RI&RS 350K (RS SD: 0.722)	Intercept	99.81	0.03	(99.75; 99.87)
	Age-at-exam	-0.88	0.002	(-0.89; -0.88)
	Sex	1.44	0.04	(1.37; 1.52)

Beta=Effect estimate of association, **SE**=Standard error, **CI**=95% confidence interval, **RI**=Random intercept, **RS**=Random slope, **SD**=Standard deviation, **age-at-baseline**=age of individual at timepoint of 1st eGFR assessment (centered at 50 years), **time**=time-since-baseline, **age-at-exam**=age of individual at timepoint of eGFR assessment (centered at 50 years). Sex is coded as 0=women, 1=men.

Supplementary Table 2: Model specifications of seven approaches for genetic association analysis with trait trajectories.
We employed seven approaches to derive genetic association with trait change: in data of individuals with ≥2 assessments over time, we applied (i) *difference model*, (ii-v) four one-stage LMM approaches, (vi) one two-stage LMM approach, and, (vii) in data adding “singletons” (i.e. individuals with =1 assessment), we repeated the LMM *age model RI&RS*. Shown are modelled outcome, covariate to estimate the SNP-association with trait change, other covariates, statistical model, stage number, and whether singletons can be and were incorporated. LMMs included random intercepts and random slopes and their correlation (RI&RS), except noted otherwise. Time refers to time-since-baseline, age to age-at-exam (age_t), age_0 to age-at-baseline (1st trait assessment). Outcome is stated for the example of eGFR.

**Approach**	**Outcome**	**Genetic effect on trait change**	**Other covariates**	**Stage**	**Statistical model**	**Incorporation of singletons (NA/no/yes)[#]**
**Individuals with ≥2 trait assessments**						
Difference model	Difference	SNP	PCs	1	LinReg	NA
Time model RI&RS	$eGFR_t$	SNPxtime	age_0, time, sex, PCs	1	LMM	no
Age model						
RI&RS	$eGFR_t$	SNPxage	age_t, sex, PCs	1	LMM	
*RI&RS uncorrelated**	$eGFR_t$	SNPxage	age_t, sex, PCs	1	LMM	no
RI-only[§]	$eGFR_t$	SNPxage	age_t, sex, PCs	1	LMM	no
BLUPs&LinReg[$]						NA
Age model RI&RS	$eGFR_t$	-	age_t, sex, PCs	1	LMM	
LinReg	RS	SNP	PCs	2	LinReg	
**Adding singletons**						
Age model RI&RS	$eGFR_t$	SNPxage	age_t, sex, PCs	1	LMM	yes

[$]: RS were extracted (via BLUPs) from LMM *age model RI&RS* without SNP as covariate (1st stage) and then used as outcome for SNP-association via linear regression (2nd stage). [#]: Incorporation of singletons is not applicable (NA), possible but not done here (no), done here (yes).

Supplementary Table 3: **Parameter specifications for simulation scenarios.**

We simulated datasets (genotypes and trait trajectories) for three scenarios, mimicking UKB and external data for trajectories of eGFR and another trait (BMI). Shown are simulation parameter values for each scenario. For the UKB scenario, we used observed age-at-exam for randomly sampled UKB 350K individuals (~50% singletons). For scenarios based on KORA-4[21], a typical cohort study with study center baseline and follow-up, we simulated 5 trait assessments 5 years apart each, with 20% attrition at each follow-up (~20% singletons). Genotypes were sampled from Binomial distribution *Bin(2, EAF),* random effects from Bivariate Normal $N(0,\Sigma)$ (diagonal elements of Σ: RI and RS variances $\sigma^2_{\gamma_0}$ and $\sigma^2_{\gamma_1}$; off-diagonal elements: RI&RS covariance $\sigma_{y_0\gamma_1} = \sigma_{y_0} * \sigma_{y_1} * \rho_{y_0\gamma_1}$, where $\rho_{y_0\gamma_1}$ is the RI&RS correlation) and residual errors from $N(0,\sigma^2)$. Phenotypes were then generated using equation (3) (**Methods**) without sex as covariate, assuming a true genetic effect on change β_{change}=0 (for T1E) or β_{change}≠0 (for power and bias).

Parameter	UKB scenario for eGFR-trajectories	KORA-4 scenario for eGFR-trajectories	KORA-4 scenario for BMI-trajectories
# Simulations	10,000	10,000	10,000
# Individuals	20,000	20,000	20,000
# Singletons (%)	≈11,000 (55%)	4,000 (20%)	4,000 (20%)
# Trait assessments per person (min-max)	6 (2-289)	4 (1-5)	4 (1-5)
Follow-up time [years] without singletons	8.4	15.8	15.8
Mean age at 1st trait assessment [years]	58	40	40
Trait unit	mL/min/1.73m²	mL/min/1.73m²	kg/m²
Global intercept* [trait units]	100	110	27
Global age effect [trait units per year]	-1.00	-1.00	0.05
Genotype EAF	0.30	0.30	0.30
True genetic main effect [per trait unit and allele]	-0.25	-0.10	0.04
True genetic change effect β_{change} [per trait unit, allele and year]#,	0.0 (T1E) -0.025 (power/bias)	0.0 (T1E) -0.010 (power/bias)	0.0 (T1E) 0.004 (power/bias)
RI variance, $\sigma^2_{\gamma_0}$	98.1	82.5	19.0
RS variance, $\sigma^2_{\gamma_1}$	52.1	10.4	1.4
RI&RS correlation, $\rho_{y_0\gamma_1}$	-0.46	0.07	0.23
Residual variance, σ^2	49.0	10.0	1.3

Singletons=individuals with =1 trait assessment, **Global intercept**=mean trait value at 1st assessment, **Global age effect**=mean trait change per year, **EAF**=effect allele frequency, **BMI**=body-mass-index, **T1E**=type 1 error, **RI**=random intercepts, **RS**=random slopes.

#: β_{change} was chosen for each simulation scenario such that power was approximately 50% for most approaches, for maximum discrimation between approaches.

Supplementary Table 4: Performance of seven approaches to genetic association analyses for trait change in independent simulated longitudinal data.

This table complements **Table 2** by adding simulations based on independent, non-UKB data. We again compared seven approaches (**Methods**, **Supplementary Table 2**) regarding type I error, power, and bias: six approaches analyze individuals with ≥2 trait assessments over age/time (no singletons, i.e. individuals with =1 trait assessment), the 7th approach repeats *age model RI&RS* including singletons. Simulations were based on distributions of age, global/random trait effects, and random error under two scenarios (as in KORA-4 for eGFR or body-mass-index, BMI, respectively) and simulated genotypes (EAF=30%; 10,000 simulation runs; **Methods**, **Supplementary Table 3**). These scenarios cover settings as in an external cohort study, KORA-4[21] (20% attrition between baseline and follow-up, thus 20% singletons) for trajectories of a trait like eGFR (prounouced age effect on trait) or another trait, BMI (less pronounced age effect). We show estimates of type 1 error (T1E), power, and bias from 10,000 simulation runs.

	**KORA-4 scenario for eGFR-trajectories**			**KORA-4 scenario for BMI-trajectories**		
**Approaches**	**T1E [%] (CI)**	**Power [%]**	**Bias [%]**	**T1E [%] (CI)**	**Power [%]**	**Bias [%]**
Without singletons						
Difference model	5.2 (4.8, 5.7)	29.5 (28.6, 30.4)	0.1	4.6 (4.2, 5.0)	31.2 (30.3, 32.1)	0.3
Time model RI&RS	5.2 (4.8, 5.7)	47.2 (46.2, 48.2)	-0.1	4.9 (4.4, 5.3)	51.4 (50.4, 52.4)	-0.4
Age model RI&RS	5.1 (4.7, 5.5)	48.6 (47.6, 49.6)	-0.3	4.9 (4.5, 5.4)	52.1 (51.1, 53.1)	-0.4
Age model RI&RS uncorr.	4.7 (4.3, 5.1)	47.3 (46.3, 48.3)	-0.3	4.1 (3.7, 4.5)	48.1 (47.1, 49.1)	-0.4
Age model RI-only	19.5 (18.7, 20.3)	68.0 (67.1, 69.0)	0.3	17.0 (16.3, 17.8)	69.9 (69.0, 70.8)	-0.4
BLUPs&LinReg	6.1 (5.7, 6.6)	71.8 (70.9, 72.7)	-11.1	5.7 (5.2, 6.2)	69.2 (68.2, 70.1)	-22.1
Including singletons						
Age model RI&RS	5.2 (4.8, 5.6)	51.2 (50.2, 52.2)	-0.3	4.8 (4.4, 5.2)	54.8 (53.8, 55.8)	-0.2

T1E=Proportion of SNPs with P_{change}<0.05 across 10,000 simulated SNPs given zero true effect on change, β_{change}=0 (95%-CI using SEs from exact binomial test); **Power**=Proportion of SNPs with P_{change}<0.05 across 10,000 simulated SNPs given true effect on change, β_{change}=-0.025, -0.01, 0.04, respectively (95%-CIs derived from SEs using exact binomial test); **Bias**=Relative bias of effect estimates given true effect on change, β_{change}=-0.025, -0.01, 0.04, respectively, derived as average (across 10,000 simulation runs) of $(\hat{\beta}_{change}-\beta_{change})/\beta_{change}$.

Supplementary Table 5: Replication of eight out of nine known variants for eGFR-decline.

We show effect estimates and P-values for eGFR-decline derived by LMM *age model RI&RS 350K* (UKB 350K; n=348,275, m=1,520,382) for the 9 variants (from 8 loci) associated with eGFR-decline previously[13]. We denote variants with directionally consistent $P_{decline}$<0.05 as replicated. In addition, we show three variants previously found to be associated with cross-sectional eGFR, but not with eGFR-decline[13]. The dataset by Gorski and colleagues[13] (n≈350,000; CKDGen) was almost independent from our UKB 150K and UKB 350K datasets (overlap: 12,840 individuals). Of note, the 9 variants previously identified for eGFR-decline in CKDGen[13] (*difference model*) consisted of 2 variants genome-wide significant ($P_{decline}<5x10^{-8}$; one locus, *UMOD/PDILT*), 2 further variants by searching the then 263 variants known for cross-sectional eGFR outside of *UMOD/PDILT* (judged at $P_{decline}<0.05/263=1.9x10^{-4}$), and 5 further variants among other pre-selected variants (judged at $P_{decline}<0.05/12=4.2x10^{-3}$).

SNPID	Locus Name	Chr	Pos	EA/OA	EAF	$beta_{decline}$	$P_{decline}$
**Replicated variants (directionally consistent, $P_{decline}$<0.05)**							
rs77924615	*UMOD/PDILT*	16	20,392,332	G/A	0.802	-0.060	1.06E-54
rs34882080	UMOD/PDILT	16	20,361,441	A/G	0.817	-0.054	4.29E-42
rs434215	*TPPP*	5	699,046	A/G	0.277	-0.026	4.26E-12
rs28857283	*C15orf54*	15	39,224,711	G/A	0.626	-0.019	3.78E-09
rs4930319	*OVOL1*	11	65,555,458	C/G	0.343	-0.014	1.35E-05
rs10254101	*PRKAG2*	7	151,415,536	T/C	0.287	-0.014	3.52E-05
rs1458038	*FGF5*	4	81,164,723	C/T	0.707	-0.014	5.33E-05
rs1028455	*SPATA7*	14	88,829,975	T/A	0.672	-0.009	5.09E-03
**Not replicated variants ($P_{decline}$>0.05)**							
rs13095391	*ACVR2B*	3	38,447,232	A/C	0.559	-0.001	0.70
**Variants confirmed as not associated with eGFR-decline ($P_{decline}$>0.05)**							
rs2453533	*SPATA5L1/GATM*	15	45,641,225	A/C	0.370	0.008	0.07
rs9998485	*SHROOM3*	4	77,362,445	A/G	0.518	-0.006	0.06
rs1047891	*CPS1*	2	211,540507	A/C	0.316	-0.004	0.18

SNPID=Variant identifier on GRCh37, **Locus name**=Nearest Gene, **Chr** and **Pos**=Chromosome and Position on genome build GRCh37, **EA/OA**=Effect allele / other allele (EA: cross-sectionally eGFR-lowering allele based on unconditioned analyses in EUR[1]), **EAF**=Effect allele frequency, $\mathbf{beta_{decline}}$ and $\mathbf{P_{decline}}$=Genetic effect estimate and P-value for eGFR-decline. For the following SNPs identified for eGFR-decline by Gorski and colleagues[13], a proxy variant was among the 595 SNPs: rs34882080 (proxy rs13334589 with r^2=0.99), rs10254101 (proxy rs10224002 with r^2=0.99), rs13095391 (proxy rs13064938 with r^2=0.84), rs1028455 (proxy rs60503594 with r^2=0.94), rs2453533 (proxy rs1145084 with r^2=0.99), and rs9998485 (proxy rs28817415 with r^2=0.49).

Supplementary Table 6: **Association of decline-associated and stable-effect variants with clinical traits**

We tested the 12+11 SNPs for association with rapid decline in a case-control comparison derived from UKB 150K individuals (annual eGFR-decline based on BLUPs < -3 for cases and -1 to +1 mL/min/1.73m² for controls; n_{cases}=1,211, $n_{controls}$=63,392; logistic regression). We also tested association with eGFR-decline in the subset of UKB 150K individuals with CKD (eGFR<60 mL/min/1.73m² at any timepoint, removing eGFR-trajectory before this timepoint; n_{CKD}=13,116, m_{CKD}=116,944); for this, we applied LMM *time model RI&RS*. Finally, we tested for association with being in the subset of CKD (cases=CKD at any timepoint, controls=no CKD at any timepoint; n_{cases}=16,147, $n_{controls}$=332,128; logistic regression). Shown are odds ratios (OR) or beta-estimates and P-values for the SNP-associations. All analyses were adjusted for age at 1st assessment, sex, and 20 PCs.

						**Rapid decline cases/controls**		**Decline in CKD**		**CKD cases/controls**	
**SNPID**	**Locus Name**	**Chr**	**Pos**	**EA/OA**	**EAF**	**OR**	**P**	**beta**	**P**	**OR**	**P**
Identified variants for eGFR-decline, known											
rs77924615	*UMOD/PDILT*	16	20392332	G/A	0.83	1.318	**7.6E-07**	-0.135	**1.3E-04**	1.252	**8.7E-44**
rs13334589	*UMOD/PDILT*	16	20366459	A/T	0.84	1.279	**1.5E-05**	-0.113	**1.4E-03**	1.206	**4.1E-30**
rs434215	*TPPP*	5	699046	A/G	0.29	1.115	**0.029**	-0.038	0.22	1.103	**1.8E-11**
rs28857283	*C15orf54*	15	39224711	G/A	0.64	1.134	**3.5E-03**	-0.034	0.21	1.051	**7.2E-05**
rs4930319	*OVOL1*	11	65555458	C/G	0.36	1.089	**0.047**	-0.040	0.14	1.070	**7.5E-08**
rs10224002	*PRKAG2*	7	151415041	G/A	0.31	1.101	**0.032**	-0.065	**0.020**	1.133	**7.4E-22**
rs1458038	*FGF5*	4	81164723	C/T	0.72	1.079	0.095	-0.020	0.049	1.055	**6.0E-05**
Identified variants for eGFR-decline, novel											
rs74209810	*UMOD/PDILT*	16	20402466	T/C	0.12	1.158	**0.020**	-0.034	0.4	1.110	**3.3E-08**
rs2783971	*SDCCAG8*	1	243474536	A/C	0.49	1.070	0.10	-0.008	0.75	1.089	**1.9E-12**
rs854922	*RRAGD*	6	90121950	A/G	0.10	1.268	**3.8E-04**	-0.108	**0.014**	1.092	**2.8E-05**
rs2076668	*GGT7*	20	33437621	A/G	0.40	1.042	0.33	-0.037	0.16	1.064	**4.4E-07**
rs2921093	*PRAG1*	8	8287383	T/G	0.59	1.063	0.15	-0.030	0.26	1.032	**0.010**
Identified variants for stable-effect on eGFR											
rs1047891	*CPS1*	2	211540507	A/C	0.33	1.059	0.19	-0.082	**2.9E-03**	1.084	**3.0E-10**
rs35969577	*DAB2*	5	39401384	T/G	0.43	0.983	0.69	0.025	0.33	1.074	**5.1E-09**
rs79760705	*ARL15*	5	53298716	G/T	0.89	1.020	0.76	-0.030	0.47	1.025	0.19
rs3812036	*SLC34A1*	5	176813404	T/C	0.26	0.992	0.86	0.008	0.79	1.083	**1.2E-08**
rs2279463	*SLC22A2*	6	160668389	G/A	0.15	1.044	0.46	-0.010	0.79	1.088	**4.8E-07**
rs13230509	*UNCX*	7	1286192	C/G	0.70	0.969	0.49	0.034	0.24	1.061	**1.4E-05**
rs10851885	*NRG4*	15	76304503	G/A	0.27	0.978	0.65	0.036	0.22	1.062	**1.4E-05**
rs9905761	*BCAS3/TBX2*	17	59239259	C/T	0.82	0.948	0.31	0.024	0.48	1.060	**2.2E-04**
rs11657044	*BCAS3/TBX2*	17	59450105	T/C	0.17	0.968	0.56	-0.048	0.17	1.049	**3.2E-03**
rs34446110	*BCAS3/TBX2*	17	59478354	G/C	0.27	0.925	0.099	0.039	0.18	1.033	**0.016**
rs35709439	*NUDT19/CEP89*	19	33190263	C/T	0.81	1.030	0.58	-0.020	0.54	1.012	0.45

SNPID=Variant identifier on GRCh37, **Locus name**=Nearest Gene, **Chr** and **Pos**=Chromosome and Position on GRCh37, **EA/OA**=Effect allele / other allele (EA: cross-sectionally eGFR-lowering allele based on unconditioned analyses in EUR[1]), **EAF**=Effect allele frequency among CKD patients in UKB 150K, **OR** and **P** (rapid decline, CKD)=Odds ratio and P-value from logistic regression, **beta** and **P** (decline in CKD)=Genetic effect estimate and P-value of association with eGFR-decline among eGFR-trajectories of CKD individuals.

Supplementary Table 7: 13 variants across 11 loci identified for association with eGFR-decline via longGWAS and/or 595-search.
Based on the LMM *age model RI&RS 350K* (UKB 350K: n=348,275, m=1,520,382), we show the 7 variants (5 loci) identified at genome-wide significance ($P_{decline}<5x10^{-8}$): 4 locus lead variants (distance>500kb, $r^2<0.01$) via longGWAS (GC-corrected, lambda=1.06); 2 additional variants at $P_{decline}<5x10^{-8}$ via the 595-search. We also show the 6 variants (6 loci) identified by the 595-search at Bonferroni-corrected significance ($P_{decline}<0.05/595$). In addition, we state whether the locus/signal was novel for eGFR-decline (compared to Gorski and colleagues[13]) at a genome-wide significance level ($P_{decline}<5x10^{-8}$), whether it was novel for eGFR-decline identified here at $P_{decline}<0.05/595$, and whether the locus/signal was known for cross-sectional eGFR-association (compared to Stanzick and colleagues[1]). Of note, *UMOD/PDILT* locus had 3 independent signals (2 known from Gorski et al., 1 novel here), while there were no multiple signals for eGFR-decline in any of the other loci.

										Locus / signal		
**Locus name**	**SNPID**	**Chr**	**Pos**	**EA/OA**	**EAF**	**$beta_{decline}$**	**$SE_{decline}$**	**$P_{decline}$**	**Source of signal**	**Novel for genome-wide significant eGFR-decline**	**Novel for eGFR-decline**	**Known from cross-sectional eGFR**
Variants associated with eGFR-decline at genome-wide significance ($P_{decline}<5x10^{-8}$)												
*UMOD/PDILT**	rs77924615	16	20,392,332	G/A	0.80	-0.060	0.004	**1.2E-54**	longGWAS + 595-search	no/no	no/no	yes/yes
*UMOD/PDILT**	rs13334589	16	20,366,459	A/T	0.82	-0.054	0.004	**1.1E-42**	595-search	no/no	no/no	yes/yes
*UMOD/PDILT**	rs74209810	16	20,402,466	T/C	0.11	-0.032	0.005	**6.6E-11**	595-search	no/yes	no/yes	yes/yes
TPPP	rs2455357[$]	5	688,900	A/G	0.30	-0.026	0.004	**2.5E-12**	longGWAS + 595-search	yes/yes	no/no	yes/yes
C15orf54	rs8036186[§]	15	39,221,020	A/C	0.59	-0.021	0.003	**3.3E-11**	longGWAS + 595-search	yes/yes	no/no	yes/yes
SDCCAG8	rs2484640[#]	1	243,462,417	G/T	0.49	-0.018	0.003	**6.1E-09**	longGWAS + 595-search	yes/yes	no/no	yes/yes
MTX1/MUC1	rs2075570	1	155,182,164	T/C	0.49	-0.017	0.003	**1.1E-08**	longGWAS	yes/yes	yes/yes	yes/no
Additional variants associated with eGFR-decline at Bonferroni-corrected significance ($P_{decline}<0.05/595$)												
OVOL1	rs4930319	11	65,555,458	C/G	0.34	-0.014	0.003	**1.3E-05**	595-search	-	no/no	yes/yes
PRKAG2	rs10224002	7	151,415,041	G/A	0.29	-0.014	0.003	**2.9E-05**	595-search	-	no/no	yes/yes
FGF5	rs1458038	4	81,164,723	C/T	0.71	-0.014	0.003	**5.3E-05**	595-search	-	no/no	yes/yes
RRAGD	rs854922	6	90,121,950	A/G	0.09	-0.024	0.005	**7.5E-06**	595-search	-	yes/yes	yes/yes
GGT7	rs2076668	20	33,437,621	A/G	0.38	-0.014	0.003	**1.6E-05**	595-search	-	yes/yes	yes/yes
PRAG1	rs2921093	8	8,287,383	T/G	0.58	-0.012	0.003	**7.3E-05**	595-search	-	yes/yes	yes/yes

SNPID=Variant identifier on GRCh37, **Chr** and **Pos**=Chromosome and Position on GRCh37, **EA/OA**=Effect allele / other allele (EA: cross-sectionally eGFR-lowering allele based on unconditioned analyses in EUR[1]), **Locus name**=Nearest gene (at *PDILT* loci, *UMOD* was added to the naming because this is a well-established kidney function gene), **Distance**=distance to nearest gene (in bp), **EAF**=Effect allele frequency, **$beta_{decline}$**, **$SE_{decline}$** and **$P_{decline}$**=Genetic effect estimate, standard error, and P-value of association for eGFR decline, **Source of signal**=Source of identification (either longGWAS or 595-search) of the signal, with signal lead variant defined to be the one from cross-sectional GWAS[1].
*: rs77924615, rs13334589, and rs74209810 are independently associated with eGFR-decline: fully conditioned $P_{decline}=1.8x10^{-17}$, $8.4x10^{-8}$, and $8.7x10^{-5}$, respectively. Highly correlated to the 12 variants identified via the 595-search: [$]: rs434215 ($r^2=0.7$); [§]: rs28857283 ($r^2=0.8$); [#]: rs2783971 ($r^2=0.9$).

Supplementary References

1. Stanzick KJ, Stark KJ, Gorski M, et al. KidneyGPS: a user-friendly web application to help prioritize kidney function genes and variants based on evidence from genome-wide association studies. *BMC Bioinformatics*. 2023;24(1):355. doi:10.1186/s12859-023-05472-0
2. Barr DJ, Levy R, Scheepers C, Tily HJ. Random effects structure for confirmatory hypothesis testing: Keep it maximal. *J Mem Lang*. 2013;68(3). doi:10.1016/j.jml.2012.11.001
3. Hoarau J-Y, Dumont T, Wei X, Jackson P, D'Hont A. Applications of Quantitative Genetics and Statistical Analyses in Sugarcane Breeding. *Sugar Tech*. 2022;24(1):320-340. doi:10.1007/s12355-021-01012-3
4. Gelman A, Hill J. *Data Analysis Using Regression and Multilevel/hierarchical Models*. Cambridge university press; 2006.
5. Hastie T, Tibshirani R, Friedman JH. *The Elements of Statistical Learning: Data Mining, Inference, and Prediction*. Springer; 2009.
6. Mountjoy E, Schmidt EM, Carmona M, et al. An open approach to systematically prioritize causal variants and genes at all published human GWAS trait-associated loci. *Nat Genet*. 2021;53(11):1527-1533. doi:10.1038/s41588-021-00945-5
7. Braissant O, Cagnon L, Monnet-Tschudi F, et al. Ammonium alters creatine transport and synthesis in a 3D culture of developing brain cells, resulting in secondary cerebral creatine deficiency. *Eur J Neurosci*. 2008;27(7):1673-1685. doi:10.1111/j.1460-9568.2008.06126.x
8. Urakami Y, Kimura N, Okuda M, Inui K. Creatinine transport by basolateral organic cation transporter hOCT2 in the human kidney. *Pharm Res*. 2004;21(6):976-981. doi:10.1023/B:PHAM.0000029286.45788.ad
9. Mi H, Muruganujan A, Thomas PD. PANTHER in 2013: modeling the evolution of gene function, and other gene attributes, in the context of phylogenetic trees. *Nucleic Acids Res*. 2013;41(Database issue):D377-86. doi:10.1093/nar/gks1118
10. Thomas PD, Ebert D, Muruganujan A, Mushayahama T, Albou L-P, Mi H. PANTHER: Making genome-scale phylogenetics accessible to all. *Protein Sci*. 2022;31(1):8-22. doi:10.1002/pro.4218
11. Gorski M, Wiegrebe S, Burkhardt R, et al. Bias-corrected serum creatinine from UK Biobank electronic medical records generates an important data resource for kidney function trajectories. *medRxiv*. 2023:1-22. doi:10.1101/2023.12.13.23299901
12. Wood S, Wood MS. Package 'mgcv'. *R package version 1.9.0*. 2015.
13. Gorski M, Rasheed H, Teumer A, et al. Genetic loci and prioritization of genes for kidney function decline derived from a meta-analysis of 62 longitudinal genome-wide association studies. *Kidney Int*. 2022;102(3):624-639. doi:10.1016/j.kint.2022.05.021
14. Rigby RA, Stasinopoulos DM. Generalized Additive Models for Location, Scale and Shape. *J R Stat Soc Ser C Appl Stat*. 2005;54(3):507-554. doi:10.1111/j.1467-9876.2005.00510.x
15. Chen H, Wang C, Conomos MP, et al. Control for Population Structure and Relatedness for Binary Traits in Genetic Association Studies via Logistic Mixed Models. *Am J Hum Genet*. 2016;98(4):653-666. doi:10.1016/j.ajhg.2016.02.012
16. Wang X, Lim E, Liu C-T, et al. Efficient gene-environment interaction tests for large biobank-scale sequencing studies. *Genet Epidemiol*. 2020;44(8):908-923. doi:10.1002/gepi.22351
17. Bates D, Martin Maechler, Ben Bolker, et al. Package 'lme4'. *URL http://lme4. r-forge. r-project. org*. 2009.
18. Machiela MJ, Chanock SJ. LDlink: a web-based application for exploring population-specific haplotype structure and linking correlated alleles of possible functional variants. *Bioinformatics*. 2015;31(21):3555-3557. doi:10.1093/bioinformatics/btv402

19. Pruim RJ, Welch RP, Sanna S, et al. LocusZoom: regional visualization of genome-wide association scan results. *Bioinformatics*. 2010;26(18):2336-2337. doi:10.1093/bioinformatics/btq419
20. Stanzick KJ, Li Y, Schlosser P, et al. Discovery and prioritization of variants and genes for kidney function in 1.2 million individuals. *Nat Commun*. 2021;12(1):4350. doi:10.1038/s41467-021-24491-0
21. Herold JM, Wiegrebe S, Nano J, et al. Population-based reference values for kidney function and kidney function decline in 25- to 95-year-old Germans without and with diabetes. *Kidney Int*. 2024. doi:10.1016/j.kint.2024.06.024
