# Supplementary material for: Analyzing longitudinal trait trajectories using GWAS identifies genetic variants for kidney function decline
Source: Nat Commun. 2024 Nov 20;15:10061. doi: 10.1038/s41467-024-54483-9 (PMC11579025; doi:10.1038/s41467-024-54483-9)
Supplement: Supplementary file 2 — Description of Additional Supplementary Files [file 41467_2024_54483_MOESM2_ESM.pdf]

Description of Additional Supplementary Files

Supplementary Data 1: Genetic association for eGFR-decline for the 595 variants across approaches.
We show results for genetic association with eGFR decline analyses for the seven approaches and the 595 SNPs. All analyses were conducted on the UKB 150K dataset (n=149,263; m=1,321,370), except for the LMM age model RI&RS 350K (UKB 350K; n=348,275, m=1,520,382); we additionally show results from the difference model by Gorski et al. (2022) on CKDGen data.
SNPID=Variant identifier on GRCh37, Type=decline/stable/neither (whether the SNP has been found to be a decline-associated or stable-effect SNP in our analyses), Chr and Pos=Chromosome and Position on GRCh37, Locus name=Nearest Gene, known SNP=yes/no (whether the SNP is one of the 9 known decline SNPs identified previously by Gorski and colleagues), EA/OA=Effect allele / other allele, EAF=Effect allele frequency, beta_decline and SE_decline and P_decline=Genetic effect estimate, standard error and P-value of decline (=SNP*age or SNP*time effect for one-stage LMMs), beta_main and SE_main and P_main=Genetic effect estimate, standard error and P-value of SNP main effect.

Supplementary Data 2: Genetic association for eGFR-decline and cross-sectional eGFR for the 595 variants.
We show summary statistics for eGFR decline analysis (LMM age model RI&RS; UKB 350K, n=348,275, m=1,520,382) as well as for cross-sectional analysis (UKB eGFR assessments from baseline study center visit only, n=341,073; linear regression adjusting for age, sex, and 20 PCs).
SNPID=Variant identifier on GRCh37, Type=decline/stable/neither (whether the SNP has been found to be a decline-associated or stable-effect SNP in our analyses), Chr and Pos=Chromosome and Position on GRCh37, Locus name=Nearest Gene, known SNP=yes/no (whether the SNP is one of the 9 known decline SNPs identified previously by Gorski and colleagues), EA/OA=Effect allele / other allele, EAF=Effect allele frequency, beta_main and SE_main and P_main=Genetic effect estimate and standard error and P-value of SNP main effect, beta_decline and SE_decline and P_decline=Genetic effect estimate and standard error and P-value of SNP*age interaction effect (decline effect), beta_cross and SE_cross and P_cross=Genetic effect estimate and standard error and P-value of cross-sectional SNP effect.

Supplementary Data 3: **Genetic association for eGFR-decline and cross-sectional eGFR as well as genetic effects at age 40&70 years for the 12 decline-associated and 11 stable-effect variants.**
We show results for eGFR-decline and average genetic effects at 40 and 70 years of age, based on the LMM age model RI&RS 350K (UKB 350K; n=348,275, m=1,520,382), among the 12 decline-associated and the 11 stable-effect variants; and results from cross-sectional eGFR analysis (UKB eGFR assessments from baseline study center visit only, n=341,073; linear regression adjusting for age, sex, and 20 PCs).
SNPID=Variant identifier on GRCh37, Type=decline/stable (whether the SNP has been found to be a decline-associated or stable-effect SNP in our analyses), Chr and Pos=Chromosome and Position on GRCh37, Locus name=Nearest Gene, known SNP=yes/no (whether the SNP is one of the 9 known decline SNPs identified previously by Gorski and colleagues), EA/OA=Effect allele / other allele, EAF=Effect allele frequency, beta_main and P_main=Genetic effect estimate and P-value of SNP main effect, beta_decline and P_decline=Genetic effect estimate and P-value of SNP*age interaction effect (decline effect), beta (40y) and beta (70y)=Total genetic effect at 40 or 70 years of age (beta_main + beta_decline*(age-50)), beta_cross and P_cross=Genetic effect estimate and P-value of SNP effect in cross-sectional analysis.

Supplementary Data 4: **Robustness of findings regarding non-linear age effects and eGFR-variability for the 12 decline-associated and 11 stable-effect variants.**

We show summary statistics of eGFR decline analyses for the LMM age model RI&RS 350K (UKB 350K; n=348,275, m=1,520,382) among the 12 decline-associated and the 11 stable-effect variants upon inclusion of a global age^2 effect and a SNP-by-age^2 effect (centered at 59 years of age). In addition, this table shows results from a GAMLSS model where a SNP main effect is tested for assocation with both eGFR-level (mean, mu) and eGFR-variability (scale, sigma), without SNP-by-age interaction.

SNPID=Variant identifier on GRCh37, Type=decline/stable/neither (whether the SNP has been found to be a decline-associated or stable-effect SNP in our analyses), Chr and Pos=Chromosome and Position on GRCh37, Locus name=Nearest Gene, known SNP=yes/no (whether the SNP is one of the 9 known decline SNPs identified previously by Gorski and colleagues), EA/OA=Effect allele / other allele, EAF=Effect allele frequency, beta_main and SE_main and P_main=Genetic effect estimate and standard error and P-value of SNP main effect from LMM age model RI&RS 350K, beta_decline and SE_decline and P_decline=Genetic effect estimate and standard error and P-value of SNP*age interaction effect (decline effect) from LMM age model RI&RS 350K, beta_decline_age2 and SE_decline_age2 and P_decline_age2=Genetic effect estimate and standard error and P-value of SNP*age^2 interaction effect (quadratic decline effect) from LMM age model RI&RS 350K, P_decline_joint=P-value for joint significance of beta_decline and beta_decline_age2 from LMM age model RI&RS 350K, beta_main_mu and SE_main_mu and P_main_mu=Genetic effect estimate and standard error and P-value of SNP main effect on eGFR-level from GAMLSS, beta_main_sigma and SE_main_sigma and P_main_sigma=Genetic effect estimate and standard error and P-value of SNP main effect on eGFR-variability from GAMLSS.

Supplementary Data 5: **Interaction analysis of 12 decline-associated and 11 stable-effect variants with diabetes and hypertension in cross-sectional data.**

We tested the 12 decline-associated and 11 stable-effect variants for SNPxage interaction in cross-sectional UKB data (using age and eGFR from baseline study center visits for individuals with available information on diabetes (DM), HbA1c, hypertension (HT), or systolic blood pressure (SBP), n=338,435) by performing linear regression of SNPxage interaction on eGFR, adjusted for sex and 20 PCs (C). We also tested for SNPxage interaction when additionally adjusting for SNP-interaction with four interaction covariates (E): diabetes (DM), hypertension (HT), HbA1c, or SBP. Finally, we also tested the four SNPxE interactions without including SNPxage. DM was defined as HbA1c≥6.5% or antidiabetic medication intake; HT was defined as antihypertensive medication intake or SBP≥140 or DBP≥90 mmHG. We show effect estimates (beta_SNPxage or beta_SNPxE), standard errors (SE_SNPxage or SE_SNPxE), and P-values (P_SNPxage or P_SNPxE). Nominally significant P-values are in bold.

Supplementary Data 6: **Annotation of genes and variants around 12 decline-associated and 11 stable-effect variants.**

The table shows the mapping of genes to the 12 decline (light green) and 11 stable (dark green) variants based on: (i) genomic position (i.e., nearest gene), (ii) functional relevance of the variant for the gene (i.e., variant is located within the gene and has a known functional consequence; based on Variant Effect Predictor annotations and CADD score >=15), and (iii) regulatory relevance of the variant for the gene in kidney tissues (i.e., variant is a significant eQTL for the gene in kidney tissue from GTEx, NEPTUNE or the Susztak lab; FDR<5%). The table further states genes that are located nearby the 12 or 11 variants (i.e., overlapping the respective cross-sectional eGFRcrea locus from Stanzick et al., NatCommun, 2021) when the nearby gene has a (iv) known kidney phenotype in mice (based on Mouse Genome Informatics data bases, MGI), (v) known kidney phenotype in human (based on OMIM, Groopman et al., N Engl J Med., 2019; or Wooperer et al., Kidney Int., 2022), (vi) known kidney drug target (based on the Thearepeutic Target Database, Zhou et al., Nucleic Acids Res., 2024) or (vii) known kidney developmental gene (based on GO terms in Panther). Any annotation except the developmental genes was extracted from the KidneyGPS online tool that contains annotations of credible set variants from a cross-sectional GWAS on eGFRcrea (https://kidneygps.ur.de/gps/). The variant rs28857283 was not contained in KidneyGPS but manually looked up in the respective data bases. OMIM entry for RRAGD was not available at the initial release of KidneyGPS and was manually added. Pathway enrichment analysis was performed using the PANTHER Overrepresentation Test (Released 20231017) with Reactome version 85 Released 2023-05-25 (Mi et al., Nucleic Acids Res., 2013; Thomas et al., Protein Sci., 2022). Aspects used for highlighting genes are marked in yellow. Note: The PRAG1 gene was named as SGK233 in KidneyGPS.

Supplementary Data 7: **Genetic association with eGFR-decline for the GMMAT/MAGEE and lme4 implementation of LMM age model RI&RS 350K among the 595 variants.**

We show results for genetic association with eGFR decline for the LMM age model RI&RS 350K (UKB 350K; n=348,275, m=1,520,382) implemented via lme4 and via GMMAT/MAGEE.

SNPID=Variant identifier on GRCh37, Type=decline/stable/neither (whether the SNP has been found to be a decline-associated or stable-effect SNP in our analyses), Chr and Pos=Chromosome and Position on GRCh37, Locus name=Nearest Gene, known SNP=yes/no (whether the SNP is one of the 9 known decline SNPs identified previously by Gorski and colleagues), EA/OA=Effect allele / other allele, EAF=Effect allele frequency, beta_decline and SE_decline and P_decline=Genetic effect estimate and standard error and P-value of SNP*age interaction effect (decline effect)
