# Supplementary material for: Analyzing longitudinal trait trajectories using GWAS identifies genetic variants for kidney function decline
Source: Nat Commun. 2024 Nov 20;15:10061. doi: 10.1038/s41467-024-54483-9 (PMC11579025; doi:10.1038/s41467-024-54483-9)
Supplement: Supplementary file 10 — Reporting Summary [file 41467_2024_54483_MOESM10_ESM.pdf]

nature portfolio

Corresponding author(s): Iris M. Heid

Last updated by author(s): Oct 2, 2024

Reporting Summary

Nature Portfolio wishes to improve the reproducibility of the work that we publish. This form provides structure for consistency and transparency in reporting. For further information on Nature Portfolio policies, see our Editorial Policies and the Editorial Policy Checklist.

Statistics

For all statistical analyses, confirm that the following items are present in the figure legend, table legend, main text, or Methods section.

n/a	Confirmed	
☐	☒	The exact sample size (*n*) for each experimental group/condition, given as a discrete number and unit of measurement
☐	☒	A statement on whether measurements were taken from distinct samples or whether the same sample was measured repeatedly
☐	☒	The statistical test(s) used AND whether they are one- or two-sided<br>*Only common tests should be described solely by name; describe more complex techniques in the Methods section.*
☐	☒	A description of all covariates tested
☐	☒	A description of any assumptions or corrections, such as tests of normality and adjustment for multiple comparisons
☐	☒	A full description of the statistical parameters including central tendency (e.g. means) or other basic estimates (e.g. regression coefficient) AND variation (e.g. standard deviation) or associated estimates of uncertainty (e.g. confidence intervals)
☐	☒	For null hypothesis testing, the test statistic (e.g. *F*, *t*, *r*) with confidence intervals, effect sizes, degrees of freedom and *P* value noted<br>*Give P values as exact values whenever suitable.*
☒	☐	For Bayesian analysis, information on the choice of priors and Markov chain Monte Carlo settings
☐	☒	For hierarchical and complex designs, identification of the appropriate level for tests and full reporting of outcomes
☐	☒	Estimates of effect sizes (e.g. Cohen's *d*, Pearson's *r*), indicating how they were calculated

Our web collection on statistics for biologists contains articles on many of the points above.

Software and code

Policy information about availability of computer code

Data collection	*Provide a description of all commercial, open source and custom code used to collect the data in this study, specifying the version used OR state that no software was used.*
Data analysis	Data processing and statistical analyses were performed using R-Software v4.0.4. LMMs were fitted using lmer() (R-package lme4 v1.1.34. For GWAS, we used GMMAT (v1.4.2) and MAGEE (v1.4.1).<br><br>The code to run the seven approaches, the GMMAT/MAGEE analysis, and the simulations is available on GitHub (www.github.com/genepi-regensburg/UKB_KidneyFunctionDecline).

For manuscripts utilizing custom algorithms or software that are central to the research but not yet described in published literature, software must be made available to editors and reviewers. We strongly encourage code deposition in a community repository (e.g. GitHub). See the Nature Portfolio guidelines for submitting code & software for further information.

Data

Policy information about availability of data

All manuscripts must include a data availability statement. This statement should provide the following information, where applicable:

- Accession codes, unique identifiers, or web links for publicly available datasets
- A description of any restrictions on data availability
- For clinical datasets or third party data, please ensure that the statement adheres to our policy

This UK Biobank project was conducted under the application number 20272. UK Biobank is a publicly accessible database. Individual participant data from UKB are available via the UK Biobank resource. Individual participant data from KORA-3, KORA-4, and AugUR are not publicly available due to data protection regulations and restrictions imposed by the Ethics Committee of the Bavarian Chamber of Physicians to protect participant privacy. However, data can be accessed upon request through project agreements with KORA (https://helmholtz-muenchen.managed-otrs.com/external) or AugUR (augur@ukr.de). For reproducibility of our results, we provide the source code for the various statistical approaches applied here (see Code Availability). We also provide the source code for the simulation studies and for the real data analysis with GMMAT/MAGEE. We provide genetic variant association summary statistics (see Supplementary Data). Source data are provided with this paper.

Research involving human participants, their data, or biological material

Policy information about studies with human participants or human data. See also policy information about sex, gender (identity/presentation), and sexual orientation and race, ethnicity and racism.

Reporting on sex and gender

Sex within the UK Biobank data was acquired from central registry at recruitment, but in some cases updated by the participant. Hence this field may contain a mixture of the sex the NHS had recorded for the participant and self-reported sex.
As stated in Table 1, 53.7% of participants were women.
All of our analyses accounted for sex as covariate.
In addition, we investigated sex-dependency of eGFR trajectories over age and robustness of results to inclusion of age-by-sex interaction effects.

Reporting on race, ethnicity, or other socially relevant groupings

We analyzed only individuals of European ancestry, owing to the data source (UK Biobank).

Population characteristics

Population characteristics are reported in Table 1.
In each of the below rows, the first entry refers to the UKB 150K dataset with >=2 eGFR assessments per individual (n=149,263, m=1,321,370), while the second entry refers to the UKB 350K dataset also including individuals with =1 eGFR assessment (n=348,275; m=1,520,382):
% (n) of women: 53.7 (80,091) 53.7 (187,129)
Number of eGFR assessments per person: 6 (2-289) 1 (1-289)
Follow-up time [years]: 8.4 (1.0-27.1) 0.0 (0.0-27.1)
Age at 1st assessment [years]: 55.9 (35.0-76.4) 57.1 (35.0-78.2)
Age at last assessment [years]: 65.1 (37.0-79.7) 60.9 (36.0-79.7)
eGFR at 1st assessment [mL/min/1.73m²]: 98.0 (15.2-192.1) 97.4 (15.0-192.1)
eGFR at last assessment [mL/min/1.73m²]: 89.4 (15.0-198.6) 94.0 (15.0-198.6)
% (n) with CKD at 1st assessment: 0.7 (1,038) 1.2 (4,069)
% (n) with CKD at any assessment: 3.8 (13,116) 4.6 (16,147)

Recruitment

The UK Biobank is a well-known, previously published data source. Brief information on recruitment is given in the legend of Supplementary Figure 1:
We started with the UKB data for eGFR trajectories integrating creatinine values from study center and eHR from “GP-clinical” as described previously (Gorski, M., Wiegrebe, S., Burkhardt, R., Behr, M., Kuechenhoff, H., Stark, K. J., ... & Heid, I. M. (2023). Bias-corrected serum creatinine from UK Biobank electronic medical records generates an important data resource for kidney function trajectories. medRxiv, 2023-12.) (2,102,174 serum creatinine measurements from 454,907 individuals after exclusion of implausible values and duplicates).

Ethics oversight

UK Biobank was approved by an ethics advisory committee. All participants have signed informed consent.

Note that full information on the approval of the study protocol must also be provided in the manuscript.

Field-specific reporting

Please select the one below that is the best fit for your research. If you are not sure, read the appropriate sections before making your selection.

☒ Life sciences ☐ Behavioural & social sciences ☐ Ecological, evolutionary & environmental sciences

For a reference copy of the document with all sections, see nature.com/documents/nr-reporting-summary-flat.pdf

Life sciences study design

All studies must disclose on these points even when the disclosure is negative.

Sample size	Detailed information on sample size is available in the legend of Supplementary Figure 1: We started with the UKB data for eGFR trajectories integrating creatinine values from study center and eHR from "GP-clinical" as described previously (2,102,174 serum creatinine measurements from 454,907 individuals after exclusion of implausible values and duplicates). We included unrelated UKB individuals of European ancestry without recorded acute kidney injury (AKI) or nephrectomy at any timepoint. We excluded eGFR-values at and after onset of renal replacement therapy (dialysis, kidney transplantation) or otherwise recorded end-stage kidney disease (ESKD), or after an observed eGFR-value <15 mL/min/1.73m². Our final UKB 350K data yielded n=348,275 individuals and m=1,520,382 eGFR assessments (199,012 individuals with =1 eGFR assessment; 149,263 individuals with ≥2eGFR assessment). Since some statistical approaches require ≥2 assessments, we also derived the UKB 150K data (n=149,263, m=1,321,370).
Data exclusions	Detailed information on sample size is available in Methods: We included unrelated UKB participants of European ancestry51 without any eHR-record of AKI or nephrectomy and without eHR-record of dialysis, kidney transplant or ESKD prior to their first eGFR assessment. We excluded eGFR assessments (i) before age of 35 years or January 1st, 1990, (ii) at or after eHR-record of dialysis, (iii) <6 months prior to, at or after eHR-record of kidney transplant or ESKD, (iv) after prior eGFR<15 mL/min/1.73m², and (v) extreme values (excluding absolute value >10 residual SDs using LMM age model RI&RS in UKB 350K; winsorizing remaining eGFR values <15 and >200 mL/min/1.73m²). We analyzed individuals with ≥2 eGFR assessments ≥1 year apart (UKB 150K), and, where applicable, added individuals with =1 eGFR assessment (UKB 350K).
Replication	Several sensitivity analyses have been conducted. See Supplementary Figure 5.
Randomization	We controlled for age, sex and 20 principal components in all eGFR-level analyses.
Blinding	Blinding was not relevant to our study because our data is observational and not experimental.

Reporting for specific materials, systems and methods

We require information from authors about some types of materials, experimental systems and methods used in many studies. Here, indicate whether each material, system or method listed is relevant to your study. If you are not sure if a list item applies to your research, read the appropriate section before selecting a response.

Materials & experimental systems

n/a	Involved in the study	
☒	☐	Antibodies
☒	☐	Eukaryotic cell lines
☒	☐	Palaeontology and archaeology
☒	☐	Animals and other organisms
☒	☐	Clinical data
☒	☐	Dual use research of concern
☒	☐	Plants

Methods

n/a	Involved in the study	
☒	☐	ChIP-seq
☒	☐	Flow cytometry
☒	☐	MRI-based neuroimaging

Plants

Seed stocks	n/a
Novel plant genotypes	n/a
Authentication	n/a
